# Genome and transcriptome of *Papaver somniferum* Chinese landrace CHM indicates that massive genome expansion contributes to high benzylisoquinoline alkaloid biosynthesis

**DOI:** 10.1038/s41438-020-00435-5

**Published:** 2021-01-01

**Authors:** Li Pei, Baishi Wang, Jian Ye, Xiaodi Hu, Lihong Fu, Kui Li, Zhiyu Ni, Zhenlong Wang, Yujie Wei, Luye Shi, Ying Zhang, Xue Bai, Mengwan Jiang, Shuhui Wang, Chunling Ma, Shujin Li, Kaihui Liu, Wanshui Li, Bin Cong

**Affiliations:** 1 Institute of Forensic Science, Ministry of Public Security, No. 17 South Muxidi Lane, Xicheng District, 100038 Beijing, People’s Republic of China; 2 College of Forensic Medicine, Hebei Medical University, Hebei Key Laboratory of Forensic Medicine, Innovation Center of Forensic Medical Molecular Identification, No. 361 Zhongshan East Road, 050017 Shijiazhuang, Hebei People’s Republic of China; 3 Novogene Bioinformatics Institute, Beijing, People’s Republic of China; 4 Hebei University, No. 180 Wusidong Road, Baoding, Hebei Province People’s Republic of China; 5 School of Life Sciences, Zhengzhou University, No. 100 Science Road, 450001 Zhengzhou, Henan People’s Republic of China; 6 Gansu Academy of Agri-Engineering Technology, No. 234 Xinzhen Road, Huangyang Town, Liangzhou District, 733006 Wuwei, Gansu People’s Republic of China; 7 Wuhan Botanical Garden, Chinese Academy of Sciences, 430074 Wuhan, People’s Republic of China

**Keywords:** Comparative genomics, Plant evolution

## Abstract

Opium poppy (*Papaver somniferum*) is a source of morphine, codeine, and semisynthetic derivatives, including oxycodone and naltrexone. Here, we report the de novo assembly and genomic analysis of *P. somniferum* traditional landrace ‘Chinese Herbal Medicine’. Variations between the 2.62 Gb CHM genome and that of the previously sequenced high noscapine 1 (HN1) variety were also explored. Among 79,668 protein-coding genes, we functionally annotated 88.9%, compared to 68.8% reported in the HN1 genome. Gene family and 4DTv comparative analyses with three other Papaveraceae species revealed that opium poppy underwent two whole-genome duplication (WGD) events. The first of these, in ancestral Ranunculales, expanded gene families related to characteristic secondary metabolite production and disease resistance. The more recent species-specific WGD mediated by transposable elements resulted in massive genome expansion. Genes carrying structural variations and large-effect variants associated with agronomically different phenotypes between CHM and HN1 that were identified through our transcriptomic comparison of multiple organs and developmental stages can enable the development of new varieties. These genomic and transcriptomic analyses will provide a valuable resource that informs future basic and agricultural studies of the opium poppy.

## Introduction

Opium poppy (*Papaver somniferum* L.), as one of the longest utilized medicinal plants in human history, has produced both great benefits and great challenges for human civilization^1^. In particular, it has been cultivated and used in traditional Chinese herbal medicine for ~1400 years^1^. The worldwide distribution of *P. somniferum* results from its long history of cultivation, and this species continues to serve as the major agricultural source for extractable pharmaceutical alkaloids used as narcotics, analgesics, and relaxants^2^. There are five main alkaloids that accumulate in the capsular latex of *P. somniferum*: morphine, codeine, thebaine, papaverine, and noscapine^2^. The opium poppy is mostly grown to extract thebaine, the first pentacyclic morphinan alkaloid^3^, which is then used as a substrate to create natural (codeine and morphine) and semisynthetic opioid alkaloids (naltrexone and hydrocodone). Morphine is the dominant alkaloid and the strongest naturally occurring analgesic. Industrial synthesis of morphine is possible, but only at low yields. Codeine is used as a cough suppressant, and papaverine, another metabolite of this pathway, is a smooth-muscle relaxant.

There are a wide variety of methods used to obtain morphine equivalents for pharmacotherapeutic treatment of health-related suffering. Impoverished populations in some developing countries lack access to pain relievers or palliative care. For example, an estimated 84% of the need for morphine equivalents in China is not met. Addressing this major global health inequality is a key priority for the World Health Organization^4^. Neither synthetic chemical nor recombinant biotechnological approaches are currently commercially viable for any of the molecules of the morphinan subclass of benzylisoquinoline alkaloids (BIAs), making opium poppy the only commercial source of such products^2,5,6^. As a result, opium poppy is a major cash crop contributing to the economies of many poppy-growing countries, such as Turkey and India^7,8^. However, due to the addictive properties of morphine, the cultivation of opium poppy without strict regulation is outlawed in China and other countries. The development of novel, high-yielding varieties through molecular marker-assisted breeding is therefore urgently needed to meet the global demand.

To date, the genomes of three species within the Papaveraceae have been published, including those of *Macleaya cordata*^9^, *Eschscholzia californica*^10^, and the high noscapine variety of *Papaver somniferum* (HN1)^11^. *Macleaya cordata* (five-seeded plume poppy) was the first to have its whole genome sequenced among BIA-producing (e.g., sanguinarine, protopine, allocryptopine, and chelerythrine) members of Papaveraceae^9^. However, the genome size of *P. somniferum* (HN1, ~2.72 Gb) is much larger than that of *M. cordata* (~378 Mb) and *E. californica* (~502 Mb). High-BIA-producing cultivars have lost substantial genetic diversity through successive bottlenecks owing to domestication and long-term selective breeding for traits that increase yield^12^. Here, we report the draft genome of the traditional CHM opium poppy and compare its gene family composition and transposable elements with those of other members of Papaveraceae to better understand the evolutionary history leading to its massive genome expansion. We also conducted in-depth transcriptomic analysis across multiple tissues and developmental stages to characterize the spatiotemporal and genetic basis of BIA synthesis in CHM compared to NH1. We further identified specific genetic variations (SNPs and InDels) that differ between these two accessions, which can lay the groundwork for the identification of allelic variants and candidate genes for introgression and germplasm improvement of commercial poppy cultivars.

## Results

### CHM genome assembly and feature annotation

**Fig. 1:**
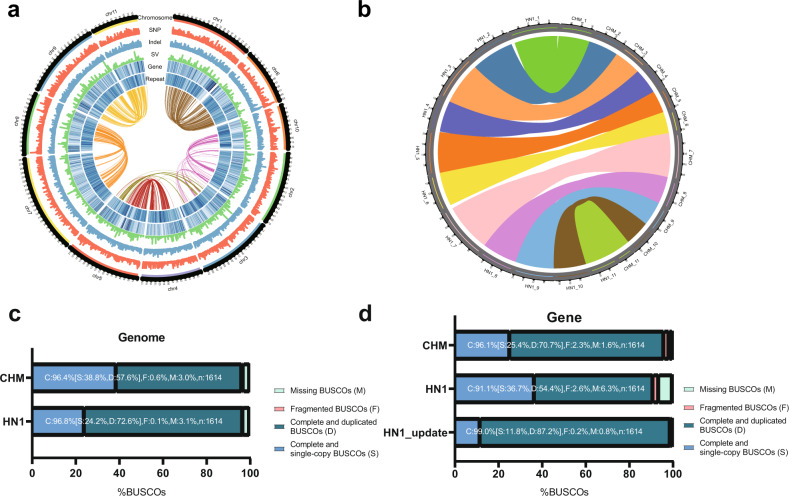
Genome comparison between the CHM assembly and published HN1 assemblies. **a** Distribution of mutations between CHM and HN1, from outer to inner: chromosome, single-nucleotide polymorphism (SNP) density, insertion or deletion density, structure variation (SV) density, CHM gene density, CHM TE density, CHM self-collinearity. Circos software (version 0.64)^50^ was used to generate Fig. 1a. **b** Genomic collinearity analysis between CHM and HN1 assemblies. **c** BUSCO analysis of CHM and HN1 assemblies. **d** BUSCO analysis of CHM and HN1 annotation sets. HN1_update refers to the updated poppy genome annotation

Greater than 956.96 Gb (~279.10 × genome coverage) of sequence data were generated from opium poppy plants (Supplementary Fig. S1) using a HiSeq X Ten instrument with read sizes ranging from 250 bp to 20 kb (Supplementary Table S1 and Supplementary Materials and Methods). Using 17-mer analysis, the genome size was estimated to be 3.37 Gb (Supplementary Fig. S2 and Supplementary Table S2). For each library, we confirmed that the raw data were not biased by measuring the distribution of insert sizes (Supplementary Table S1). After filtering, the genome was assembled into 2.62 Gb (77.74% of the estimated genome size) with a scaffold N50 of 6.86 Mb determined using Platanus^13^ and other scaffolding software described in the “Materials and methods” section; 90% of the genome assembly was contained in 2303 scaffolds. Through three-dimensional proximity information generated by chromosome conformation capture sequencing^14^, we linked the scaffolds into superscaffolds using SALSA. Then, nucmer (version 3.23) was used to anchor superscaffolds to the HN1 genome^11^. The chromosomal locations of blocks mapped to the HN1 genome were retrieved for anchoring and orienting superscaffolds to the corresponding chromosome (Supplementary Tables S3 and S4). The final assembly comprised 11 pseudochromosomes (87.6% of the genome) (Supplementary Table S5), with the longest scaffold (chromosome) of 287.96 Mb and superscaffolds N50 of 227.38 Mb (L50 = 5) (Fig. 1a, Table 1 and Supplementary Table S4).

**Table 1:** Summary of genome assembly and annotation

*Assembly*	
Genome-sequencing depth (*x*)	279.10
Estimated genome size (Gb)	3.37
Number of scaffolds	358,674
Total length of scaffolds (bp)	2,622,282,926
N50 of scaffolds (bp)	6,863,289
Longest scaffolds (bp)	31,363,288
Number of contigs	473,482
Total length of contigs (bp)	2,540,151,652
N50 of contigs (bp)	86,036
Longest contigs (bp)	626,332
Predicted coverage of the assembled sequences (%)	77.74
GC content of the genome (%)	37.29
*Annotation*	
Number of predicted protein-coding genes	79,668
Average gene length (bp)	2440.70
Percentage of gene length in the genome (%)	7.42
Mean exon length (bp)	234.91
Average exon per gene	4.21
Mean intron length (bp)	452.65
tRNAs	5226
rRNAs	1129
snRNAs	2076
miRNAs	1404
Masked repeat sequence length (Mb)	1723.84
Percentage of repeat sequences (%)	65.79

The completeness of gene regions was evaluated using Benchmarking Universal Single-Copy Orthologs (BUSCO, version 4.0.5). Of the 1614 single-copy orthologs identified in embryophytes, 96.4% were more complete in our assembly than in that of HN1 (93.1%) (Fig. 1c). CEGMA assessment^15^ of CHM showed that this assembly captured 96.77% (240 of 248) of the core eukaryotic genes and that 94.35% (234/248) were complete (Supplementary Table S6). To further verify the accuracy of the assembly, we used nucmer (version 3.23) and MCScan to analyze collinearity with HN1 and found a high degree of synteny across the whole genome (Fig. 1b and Supplementary Fig. S3).

We identified 1.69 Gb (65.79%) of the assembled CHM genomes as transposable elements (TEs) (Table 1 and Supplementary Table S7). The predominant type of TE was long terminal repeat (LTR) elements, which represented ~54.4% (1.43 Gb) of the total genome of TEs. Most LTRs were Ty3/*Gypsy* elements, which accounted for 64.5% (25,021/38,803) of TEs in CHM. A large number of Caulimoviridae elements were also unique to opium poppy (Fig. 1a, Supplementary Fig. S4 and Supplementary Table S8). However, in HN1, ~71% of the genome was identified as repetitive, with LTRs, in particular, comprising 45.85% of the genome^11^.

A total of 79,668 protein-coding genes (PCGs) were predicted in the CHM genome, with a mean coding sequence size of 988.54 bp and an average of 4.21 exons per gene (Supplementary Fig. S5 and Supplementary Table S9). This analysis identified a comparable number of PCGS in CHM to that of the updated HN1 genome (82,963) but a higher number than the original^11^.

Among these PCGs, 59.6% were also observed in the RNA-seq data (RPKM > 1), and 69% had protein homologs in *Vitis vinifera*, *Arabidopsis thaliana*, *Oryza sativa*, *Nelumbo nucifera*, *Aquilegia coerulea*, and *Amborella trichopoda* (Supplementary Fig. S6 and Supplementary Table S10). In total, ~85.3% of the genes were supported by at least two lines of evidence (i.e., RNA-seq data, homology, and de novo prediction). BUSCO analysis indicated that PCG annotations in CHM were similar to that of the updated HN1 annotation, both of which had a higher degree of completeness than the original HN1^11^ (Fig. 1d). Among these PCGs, 88.9% could be functionally classified based on information from the NR (non-redundant database in NCBI), SwissProt, InterPro, Pfam, and KEGG databases (Supplementary Table S11). In addition, 5226 transfer RNA genes, 1404 miRNA genes, 2076 small nuclear RNA genes, and 1129 ribosomal RNA genes were also predicted (see supplemental methods) in the genome (Table 1 and Supplementary Table S12).

### The LTR-retrotransposon families are drivers of the expanded opium poppy genome

**Fig. 2:**
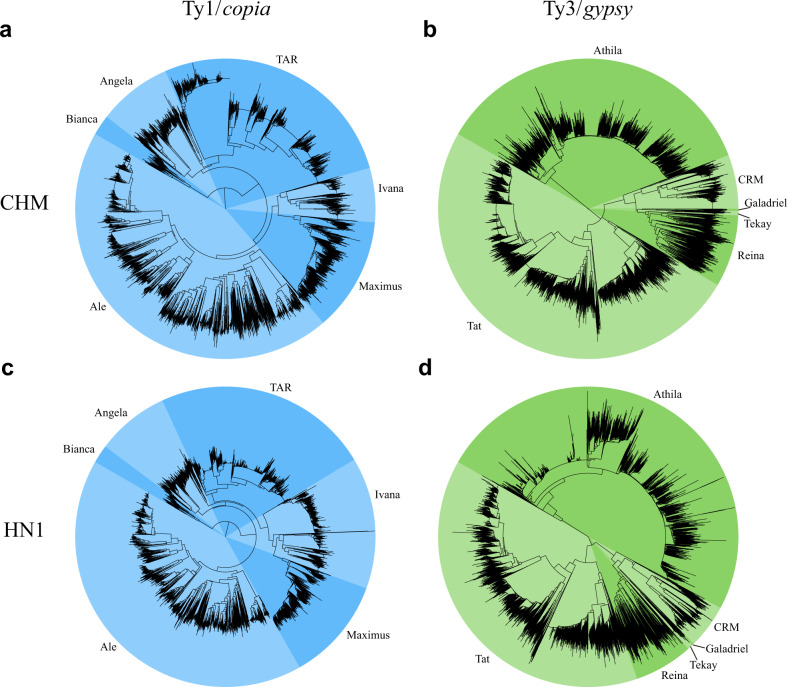
Phylogenetic analysis of the LTR-retrotransposon sequences in the CHM and HN1 genomes. The neighbor-joining and unrooted phylogenetic trees were constructed on the basis of 3107 CHM Ty1/*copia* (**a**), 6278 CHM Ty3/*gypsy* (**b**), 3372 HN1 Ty1/*copia* (**c**), and 7502 HN1 Ty3/*gypsy* (**d**) aligned sequences

Transposable elements are essential for the formation of genome structure, especially for LTRs, which are the most prevalent repeats in plant genomes, and the proliferation of these repeats reportedly leads to genome bloating. Using our chromosome-based genome assembly and previously published chromosome-level genome^11^, we investigated the evolution of LTR retrotransposons and their potential contribution to the growth of the opium poppy genome. The *P. somniferum* CHM genome was approximately six times larger than that of *M. cordata* primarily^9,10^ owing to the accumulation of more repetitive sequences (~1.72 Gb)^9^ (Supplementary Tables S13 and S14). While LTRs comprised the most abundant repeat type in *P. somniferum*, their composition differs from that described in other plant genomes of the Papaveraceae family. We estimated the total number of LTR retrotransposons by counting the number of reverse transcriptase (RT) domains encoded by the *P. somniferum* (CHM and HN1) (Fig. 2), *M. cordata*, and *E. californica* genomes (Supplementary Fig. S7). Of the RT domains, the numbers of the Gypsy and Copia families were ~15- and ~7-fold higher in CHM than in *M. cordata*, respectively (Fig. 2 and Supplementary Fig. S7). This finding led us to conclude that the substantial proliferation of the Gypsy and Copia families was the most likely main factor driving the expansion of the opium poppy genome.

To systematically investigate the potential genome expansion event in CHM, we used divergence analysis to examine the insertion time of all TEs^16^. As shown in Supplementary Fig. S4, the high peak representing the point of most frequent insertion activity showed a substitution rate of ~41%, suggesting that all of the TEs were amplified nearly simultaneously. To verify the insertion time and clarify the details of this genomic expansion through TE proliferation, we further dated each class of TEs in CHM and *M. cordata*. We found that all TE components in both CHM and *M. cordata* underwent simultaneous amplification in the ancient past (Supplementary Fig. S8). In addition, CHM underwent a second, more recent burst of TE proliferation, which potentially resulted in the genome bloat currently observed in modern opium poppy.

Unequal intraelement homologous recombination (UIHR), which produces solo LTRs, is considered one of the major processes leading to the removal of LTR-RT DNA in plants^17–19^. Over time, with an increasing number of UIHR events, the ratio of solo LTRs to intact elements (S/I ratios) should increase. To check the rate of LTR disappearance in the CHM genome, we compared the solo LTRs between the *M. cordata* and CHM genomes. We found that the S/I ratio in the CHM genome (31,308/76,380 = 0.4) was less than that in the *M. cordata* genome (6370/5207 = 1.2) (Supplementary Table S16), suggesting a higher frequency of recent LTR insertion activity in CHM.

### Gene family analysis and whole-genome duplication (WGD)

**Fig. 3:**
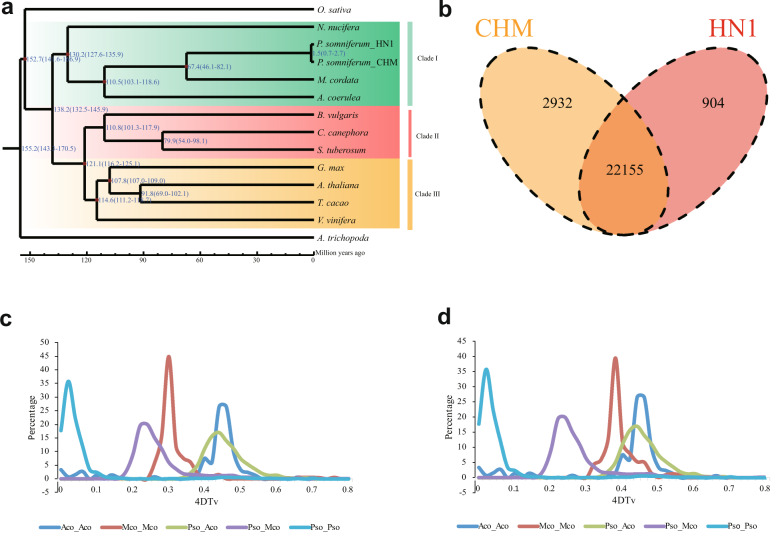
The landscape of opium poppy gene families. **a** Divergence time of 14 species. A phylogenetic tree was constructed based on 35 high-quality 1:1 single-copy orthologous genes with *A. trichopoda* serving as an outgroup. **b** Venn diagram shows the shared and unique gene families between the CHM and HN1 genomes. **c** 4DTv analysis showing a whole-genome duplication event in CHM (Pso), *Macleaya cordata* (Mco), and *Aquilegia coerulea* (Aco). The rate of evolution of Mco was not corrected. **d** The same analysis as **c** but with a corrected rate of evolution for Mco

To better understand the impact of genomic expansion on the evolution of *P. somniferum* through the examination of its repertoire of gene families, we compared the sequence similarity of the predicted proteomes of CHM, HN1, and 12 other representative angiosperm species using OrthoMCL^20^ (Fig. 3a). In total, we found 39,926 gene families, among which 6696 were shared among the 14 species and 35 single-copy orthologous gene families in each species (Supplementary Fig. S9). Furthermore, a comparison between the two *P. somniferum* genomes revealed that 2932 and 904 gene families were unique in the CHM and HN1 genomes, respectively (Fig. 3b). Kyoto Encyclopedia of Genes and Genomes (KEGG) pathway analyses (FDR < 0.05) showed that the unique genes in CHM were mainly enriched in functional categories involved in carbon fixation and metabolism, such as photosynthesis (map00195), ABC transporters (map02010), carbon metabolism (map01200), amino sugar and nucleotide sugar metabolism (map00520). They were also enriched in the metabolic pathways related to the biosynthesis of isoquinoline alkaloids (map00950) and the synthesis of tropane, piperidine, and pyridine alkaloids (map00960) (Supplementary Table S15). This result showed that CHM, as a wild accession, can provide a valuable genetic resource for germplasm improvement of the domesticated poppy. While the unique genes in HN1 were also enriched in carbon fixation and metabolism, unlike CHM, unique genes in HN1 were enriched in resistance-related metabolic pathways, such as glutathione metabolism (map00480), biosynthesis of unsaturated fatty acids (map01040), and fatty acid biosynthesis (map00061), possibly related to long-term artificial selection for secondary metabolite production (Supplementary Table S16).

Based on the 35 single-copy orthologous genes identified in the 14 plant species, we constructed a high-confidence phylogenetic tree using the MUSCLE (version 3.8.31) alignment and RAxML (version 8.0.19) package with the maximum likelihood method and estimated the divergence times using the PAML package (version 4.7a)^21,22^ (Fig. 3a). We found that *P. somniferum* (CHM and HN1), *N. nucifera*, *A. coerulea*, and *M. cordata* were clustered in the same branch, which is consistent with their previously reported phylogenetic relationship, with all belonging to a basal eudicot clade. In addition, we found that the Papaveraceae family (*P. somniferum* and *M. cordata*) diverged from the Ranunculaceae family ~110 Mya, relatively soon after the divergence from *N. nucifera* (Fig. 3a). In contrast, CHM and HN1 diverged ~1 Mya.

The expansion or contraction of gene families can be an important driver of lineage splitting and phenotypic specialization^23,24^. We found that 1616 and 961 gene families significantly expanded, and 681 and 1188 gene families exhibited contraction in CHM and HN1, respectively. For the two opium poppy accessions, 759 exhibited significant expansion, and 17 gene families exhibited contraction (Supplementary Fig. S10). Further functional characterization based on Gene Ontology (GO) (FDR < 0.05) and KEGG analyses (FDR < 0.05) revealed that genes in these expanded families were mainly enriched in functional categories associated with isoquinoline alkaloid biosynthesis (map00950), strongly suggesting that they are part of the genomic basis for the high content of isoquinoline, including papaverine and morphine, in *P. somniferum*. In addition, the results showed that these genes were enriched in the function of tyrosine metabolism (map00350), which serves as the initial substrate of many components in isoquinoline alkaloid biosynthesis^25,26^ (Supplementary Table S17). Notably, common expansion gene families were significantly enriched in a number of plant resistance-related functions. For instance, the defense response (GO: 0006952) was significantly enriched in the expanded gene families (Supplementary Table S18). Hence, we think that plant resistance was specialized in some gene families, accompanied by attenuation of other related gene families in *P. somniferum*. Some of the genes involved in isoquinoline alkaloid biosynthesis in KEGG were also presented in GO terms of plant defense, anastomosing extensively with the defensive status of isoquinoline alkaloid. Additionally, genes involved in the transport process were exceedingly enriched.

Polyploidy and whole-genome duplication (WGD) events, in particular, have been a major evolutionary force for genome evolution in angiosperms. The core eudicots are a product of genome triplication after divergence from the basal eudicots. However, it remains unclear when and how polyploidy arose in early-diverging eudicots, for example, as an ancestral feature of Ranunculales.

To explore this possibility, we used 4DTv analysis to look for evidence of WGD events in the *P. somniferum* CHM, *M*. *cordata*, and *A. coerulea* genomes. Comparison of 4DTv values of *M. cordata*–*A. coerulea* with *P. somniferum*–*A. coerulea* showed that the average value was 24.5% lower, suggesting that the evolutionary rate of *M. cordata* is much slower than that of *P. somniferum* (Supplementary Table S19). We thus adjusted the 4DTv distributions of *M. cordata*–*M. cordata* accordingly^27^ and found that the paralog peaks of *M. cordata*, *P. somniferum*, and *A. coerulea* occupied almost the same position, strongly implying that Ranunculales may have had a common WGD event. For *P. somniferum*, we observed another peak at 4DTv values of 0.0–0.06 (Fig. 3c, d), indicating that CHM likely underwent a more recent, intraspecific WGD event, which may partially explain the significant genome expansion of *P. somniferum*.

### Evolution of the benzylisoquinoline alkaloid biosynthesis pathway

BIAs are a large and structurally diverse class of metabolites that exhibit a range of biological and pharmacological properties. Among them are the narcotic analgesic morphine, the antitussive and mitotic inhibitor noscapine, the vasodilator papaverine, and the antimicrobial sanguinarine^28^. These addictive but medicinally potent metabolites occur at high concentrations in opium poppy.

**Fig. 4:**
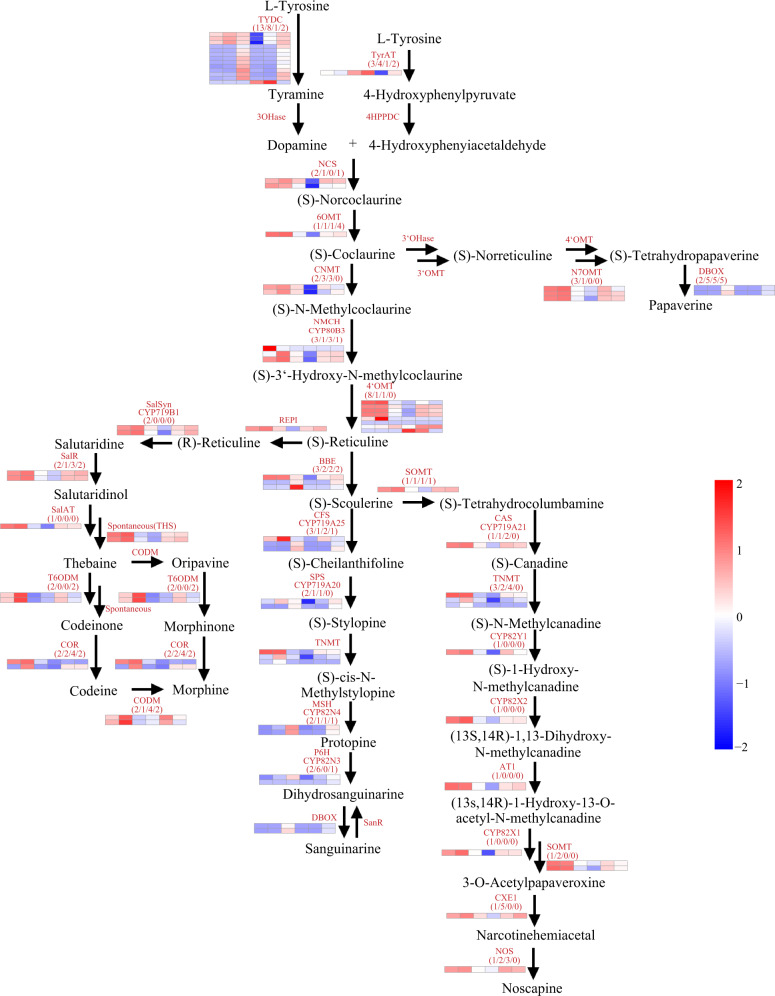
Gene expression in BIA metabolism pathways. The four numbers in parentheses indicate gene copy numbers in *Papaver somniferum* (Pso), *Macleaya cordata* (Mco), *Aquilegia coerulea* (Aco), and *Nelumbo nucifera* (Nnu). The heatmaps represent the gene expression level in two different Pso samples (HN1 and CHM). In each heatmap, the columns from left to right represent stem (stem of HN1, one day after the onset of anthesis), stem_5days (stem of HN1, five days after the onset of anthesis), stem_c (stem of CHM in fructescence), stem_cz (stem of CHM in the growth period), stem_gc (stem of CHM in early fruit development), and stem_gz (stem of CHM in the metaphase of fruit development)

We reconstructed a BIA metabolic pathway in our CHM genome based on the published sequence of cloned and validated cDNA. After homology alignment and domain identification (see “Materials and methods” section), 106 loci, including multiple copies of a total of 31 distinct genes, were detected. With the exception of unknown genes, this suite of genes encompassed the complete set of BIA metabolic pathways, including the “core” route, as well as the morphine, thebaine, noscapine, sanguinarine, and papaverine branches (Fig. 4 and Supplementary Table S20). Among them, (*S*)-norcoclaurine, the common precursor of all BIAs, is formed from dopamine and 4-hydroxyphenylacetaldehyde (4-HPAA) by (*S*)-norcoclaurine synthase (NCS). Therefore, NCS has a central role in the BIA metabolic pathway, and its catalytic activity has been reported to be particularly high in members of the Papaveraceae and Ranunculaceae that produce BIAs.

The structural diversity of BIAs resulted from the highly heterologous metabolic pathway, including several enzyme types, such as *O*-methyltransferase (OMT) and berberine bridge enzyme (BBE). We therefore carefully explored our genomic and transcriptomic data to check whether *3’OHase* and *3’OMT* were overlooked. Using the annotated gene set and protein-domain information, 58 candidate genes containing the PF08100 (dimerization): dimerization domain and PF00891 (Methyltransf_2): *O*-methyltransferase were selected for further analysis. We then filtered out 14 genes due to their previous identification as components of BIA biosynthesis pathways, and 44 genes were retained for further correlation analysis based on their expression patterns (Pearson’s correlation test, *r* ≥ 0.9)^29^. As a result, 13 genes were identified as candidate *3’OHases* or *3’OMTs*, in addition to six genes with *r* ≥ 0.95 (Supplementary Table S21 and Supplementary Fig. S11). Since berberine bridge enzyme (BBE) is significant in sanguinarine, chelerythrine, and noscapine synthesis in plants, it catalyzes the conversion of (*s*)-reticuline to (*s*)-scoulerine. Thus, we further explored potential BBE genes in CHM. Using the above methods, we identified three candidate BBE genes that harbor the PF08031 (BBE) and PF01565 (FAD_binding_4) domains (Pearson’s correlation test, *r* ≥ 0.9)^30^ (Supplementary Table S22 and Supplementary Fig. S12).

We also checked for the presence of this suite of BIA gene orthologs in related species, including *M. cordata*, *N. nucifera*, and *A. coerulea*. A comparative analysis of these genes indicated that the majority of genes encoding enzymes involved in the “core” pathways from l-tyrosine to (*s*)-reticuline^25^ could be found in all four of these plants, except *4OMT* in *N. nucifera*, although copy number varied sharply among them. Notably, the divergence of genes involved in characteristic BIA metabolite pathways was obvious between species (Supplementary Table S23). For example, in the morphine biosynthesis branch, one copy each was found for *STORR*, *SalSyn*, and *SalAT* in *P. somniferum*, but no copy was found in other species. Similar distribution patterns were also found for *N7OMT* in papaverine biosynthesis and other genes in noscapine and sanguinarine biosynthesis. These differences in BIA biosynthesis between *P. somniferum* and related species suggest a basis for phenotypic differentiation among the Papaveraceae. For example, *DBOX* proteins were significantly expanded in *M. cordata*, suggesting a likely basis of its capacity for sanguinarine biosynthesis. In addition, chromosomal distribution analysis showed that more than ten genes involved in noscapine biosynthesis belong to apparent gene clusters in the CHM genome (Supplementary Fig. S13B).

To gain insight into the molecular mechanisms and regulatory processes of BIA biosynthesis, we further examined the expression patterns of genes potentially involved in these processes through analysis of ~340 Gb of RNA-seq data generated from 45 samples from four tissues (root, stem, leaf, and fruit) collected at different developmental stages: the growth period (CZ), early fruit period (GC), metaphase period of fruit development (GZ), and frutescence period (C)^31^. We found that the BIA-related genes exhibited distinct tissue-specific expression patterns at all developmental stages. In brief, morphine is typically synthesized in roots during the CZ stage, whereas morphine, codeine, and thebaine are primarily synthesized in stem tissue in the GC and CZ stages. The genes related to noscapine biosynthesis are highly expressed in the root throughout all stages but only after fruit formation in stems. Papaverine biosynthesis gene expression was mainly observed in root tissue at all developmental stages but was highly expressed in the stem during the GC stage. Additionally, we obtained several other potentially interesting results from the differential expression analysis. For example, many multicopy genes, such as *BBE* and *4OMT*, were divergently expressed between copies, either being differentially expressed between tissues (tissue-specific expression) or individual copies were expressed at different stages (stage-specific), suggesting possible differences in their regulatory elements. Detailed results are shown in Supplementary Fig. S14. In general, BIAs are produced mainly in roots and stems and then transferred to other tissues, such as fruit^26,32^, which supports our findings of significant functional enrichment for metabolite transport among the expanded gene families.

We also compared the expression patterns between CHM and HN1 (Supplementary Figs. S15–19) and found that in HN1, transcription of noscapine synthesis pathway genes was higher than in CHM, corresponding to the higher synthesis of noscapine reported in HN1^11^. The same trend was observed for papaverine synthesis pathway-associated genes. In contrast, we found that sanguinarine synthesis pathway genes were expressed more highly in CHM than HN1. In the morphine synthesis pathway, the pattern was less clear, and some genes had higher expression in CHM, while others had higher expression in HN1. In the “core” pathway, genes in HN1 were expressed at higher levels than in CHM, which supported reports of overall higher BIA alkaloid biosynthesis in HN1^11^.

### Variation between CHM and high noscapine 1 (HN1) varieties

To identify the differences between CHM and HN1^11^, we designed a computational pipeline that takes advantage of the sequencing reads as well as assembled genomes to catalog variation, including single-nucleotide polymorphisms (SNPs) and insertions or deletions (InDels). In total, 2031.83 Mb of homologous blocks were identified in CHM and HN1, with identities of 98.76% and 98.77%, respectively. We detected a total of 12,563,129 SNPs, among which 309,236 were located within coding sequences (CDSs), while 133,154 were nonsynonymous SNPs. The number of SNPs in 2k windows was counted. Each chromosome was cut into 2k windows, and the number of SNPs in each window ranged from 1 to 2196 (Supplementary Fig. S20). Therefore, the SNP density distribution on each chromosome was uneven, with an average density of 4.8 SNP/kb. We detected 1,612,314 InDels, including 19,158 in exonic regions. A search of InDels with lengths equaling multiples of 3 bp revealed their overrepresentation in coding regions. A total of 11,470 InDels led to frameshifts, and 860 InDels resulted in gain or loss of the stop codon (Supplementary Table S25). We also detected 2193 deletions and 2204 insertions >50 bp in length (Supplementary Table S26). Genes adjacent to or flanking these variations were selected for functional characterization by GO analysis. Among these, 79 genes were inferred to be involved in oxidation–reduction processes, 42 genes were predicted to be involved in protein phosphorylation, and 25 genes were purportedly involved in transcriptional regulation (Supplementary Table S27). Figure 1 illustrates the distribution of SNPs, InDels, and structural variations between CHM and HN1 across the whole genome.

We also detected one deletion and 15 inversions with lengths >50 bp in the BIA gene cluster. (Supplementary Table S28). These findings suggest that these SNP and InDel variations may affect the coexpression of genes within this cluster. MUMmer plotting of this region between HN1 and CHM was performed, which supports the regions of CHM and HN1 as having similar genomic structures (Supplementary Fig. S13A).

## Discussion

The opium poppy is one of the earliest known and persistently used medicinal plants. A closer study of its genome and comparison with related species can clarify how narcotic analgesics such as morphine and codeine are biosynthesized, thus informing the development of semisynthetic derivatives such as oxycodone and naltrexone^11^. The draft genome and transcriptome presented in this study serve as a foundation for deeper exploration of the genetic basis of agronomically important traits and the characteristic physiological and medicinal properties of the opium poppy. The availability of Chinese opium poppy landrace genomes can also facilitate in-depth fundamental comparative studies on the biology of this species, thereby addressing a wealth of questions regarding *Papaver* genes and genome evolution. These questions may be particularly informative in the improvement of *P. somniferum* germplasm, given its role as the most economically valuable BIA-producing crop and the fact that it is horticulturally distinct from other nontoxic opium poppy varieties (*Papaver* genus).

Long terminal repeat retrotransposons (LTR-RTs) contribute to the formation and evolution of genome size^33^, in some plant species comprising > 75% of the genome and serve as a driving factor in genome expansion^34^. Opium poppy, in particular, possesses an extraordinarily large genome compared with the genomes of its related species *M. cordata* and *E. californica*. Among these repeat sequences, members of the *Gypsy* and *Copia* families in CHM and HN1 were twice as abundant as those in *M. cordata* and *E. californica*. We dated each TE element in CHM and *M. cordata* and found that CHM was subject to a relatively recent TE burst. The SI of CHM (0.40), substantially smaller than that of *M. cordata* (1.20), also indicates a recent LTR-RT amplification in CHM. We thus propose that the substantial proliferation of LTRs is a likely major cause for the large CHM genome size.

Polyploidy, particularly WGD, has been a prominent feature in the evolutionary history of angiosperms^35,36^. One study proposed that opium poppy underwent a WGD event at ~7.8 Mya, in addition to ancient segmental genome duplication or WGDs that occurred prior to the Papaveraceae–Ranunculaceae divergence at 110 Mya^11^. In our study, we found that *P. somniferum_*CHM, *M. cordata*, and *A*. coerulea have a common paralog peak, indicating that Ranunculales may share a common WGD event. Compared with related species, opium poppy also had a recent, intraspecific WGD, which provided a major contribution to the genome evolution of modern accessions. For example, our analyses suggest that such a WGD event may have facilitated the expansion of gene families related to major secondary metabolite production (e.g., isoquinoline alkaloid) as well as disease resistance, at least partially explaining the higher isoquinoline content and broad environmental adaptability of the opium poppy.

In this work, we also detected genes related to BIAs in the CHM genome. Differential expression of these genes indicated a potential genetic base underlying phenotypic divergence among Papaveraceae and related species. Specifically, some BIA-related genes were highly expressed in the root or stem at some or all developmental stages, suggesting that BIAs are mainly produced in the root and stem and then transported to other tissues such as fruit. This study thus provides a relatively comprehensive investigation into the major alkaloid-producing organs of opium poppy, thereby modifying our current understanding of the physiology of BIA metabolites in this species. Moreover, our results are consistent with those of previous studies that explored the cytology and biodynamics of opium poppy^37^.

In addition to transcriptional differences, we also identified many genetic variations (SNPs and InDels) between the CHM and HN1 varieties. In contrast to CHM, HN1 exhibits higher noscapine content in the stem, which has been used as an antitussive for more than 100 years. We found that the ten genes involved in noscapine biosynthesis are located in a 584-kb BIA gene cluster on chromosome 11 and are coexpressed in the stem. Within this BIA gene cluster, we detected 1242 SNPs and 166 InDels, among which 52 SNPs and one InDel were located in exonic regions. We detected 25 SNPs that resulted in nonsynonymous mutations and two SNPs that resulted in stop codon gain in CHM (Supplementary Tables S29 and S30). Notably, the expression levels of genes in the noscapine biosynthesis cluster were significantly lower in CHM than in HN1. We also found a large number of long-segment insertions in the CHM noscapine gene cluster, offering a preliminary explanation for the higher noscapine content in HN1. Further study will explore possible variations in the regulatory mechanisms that drive higher noscapine synthesis in NH1. Newly identified genetic variations in these genomic regions that have been fixed in CHM can potentially be used to design crosses to determine if they contribute to agriculturally valuable phenotypes and can thus serve as viable candidate genes for the development of new varieties^12^.

In summary, the genome sequencing of Chinese opium poppy landraces expands the available genetic information for this valuable medicinal species, thus enabling the systematic study of the biosynthesis, regulation, and transportation of BIAs for agronomic and pharmaceutical purposes. The genome assembly and analysis provided in this work will be a useful resource for future studies that assess the pharmacology, chemical constituents, cultivation, genetic improvement of traits, and populations of the opium poppy.

## Materials and methods

### Sample information, library construction, and genome sequencing

DNA samples were harvested from the leaf tissues of an individual opium poppy plant, which was provided by Wuhan Botanical Garden, Chinese Academy of Sciences (Supplementary Fig. S1). High-quality genomic DNA was extracted from frozen leaf samples by a modified CTAB method^38^. The quality and quantity of the isolated DNA were checked by electrophoresis on a 0.8% agarose gel and a NanoDrop D-1000 spectrophotometer (NanoDrop Technologies, Wilmington, DE) as well as a Qubit Fluorometer, respectively. With the qualified DNA, three types of short-insert paired-end libraries (250, 300, and 450 bp) were prepared using the NEBNext Ultra DNA Library Prep Kit for Illumina (NEB, USA), and four types of mate-pair libraries (2, 5, 10, and 20 kb) were prepared using Illumina’s DNA library preparation kits (TruSeq PE Cluster Kit v3, cBot, HS; and TruSeq SBS Kit v3, HS [200 cycles]) according to the manufacturer’s protocol. The Hi-C library was also constructed using fresh leaves according to the manufacturer’s instructions. Illumina paired-end sequencing libraries were generated following the manufacturer’s standard protocol (Illumina) and sequenced on the Illumina HiSeq platform (Illumina, San Diego, CA).

### Genome assembly and quality assessment

Platanus Genome Assembler (v1.2.4)^13^ was used to assemble all the high-quality reads into scaffolds with parameters “-c 15 -k 60 -t 50 -m 300”. GapCloser (http://sourceforge.net/projects/soapdenovo2/files/GapCloser) was adopted with parameters “-p 25 -l 150” to fill gaps in the assembled scaffolds using PE reads. Read mapping, BUSCO, CEGMA, and BAC alignment were used to evaluate the quality of the assembled genome.

### Repeat annotation

Two approaches were used to discover repeat elements: de novo predictions and homolog-based identifications. RepeatModeler (http://www.repeatmasker.org/RepeatModeler/,vision 1.0.5) and LTR_FINDER^39^ were used to build a de novo repeat library, followed by analysis with RepeatMasker (http://www.repeatmasker.org, version 3.3.0) to discover TEs. RepeatMasker and RepeatProteinMask were involved in homology-based identifications to detect TEs by comparing them to Repbase.

### Genome annotation

Protein-coding genes in the CHM genome were predicted using a combination of homology-based prediction, de novo prediction, and transcriptome-based prediction methods. Five ab initio gene prediction programs were used to predict genes, including Augustus (http://Augustus. gobics.de/, version 2.5.5), Genescan (http://genes.mit.edu/GENSCAN.html, version 1.0), Geneid (http://genome.crg.es/software/geneid/), GlimmerHMM (http://ccb.jhu.edu/software/glimmerhmm/, version 3.0.2) and SNAP (http://korflab.ucdavis.edu/software.html, version 2013–11–29). Protein sequences of six homologous species (*Vitis vinifera*, *Arabidopsis thaliana, Oryza sativa, Nelumbo nucifera, Aquilegia coerulea*, and *Amborella trichopoda*) were downloaded from Ensembl or NCBI. Homologous sequences were aligned against the repeat-masked CHM genome using TBLASTN^40^ (*E*-value ≤ 1E−05). Genewise (https://www.ebi.ac.uk/Tools/psa/genewise, version 2.2.0) was employed to predict gene models based on the alignment sequences. The RNA-seq data were mapped to the CHM genome using Tophat (http://ccb.jhu.edu/software/tophat/index.shtml, version 2.0.8)^41^, and cufflinks (http://cufflinks.cbcb.umd.edu/, version 2.1.1)^42^ was then used to assemble the transcripts into gene models. Trinity (version 2.0.8) was used to de novo assemble the RNA-seq data. A weighted and non-redundant gene set was generated by EVidenceModeler (EVM)^43^, which only keeps the longest model per locus. Then, PASA software (version 2.0.2) (http://pasapipeline.github.io/)^43^ improved the gene structures. Finally, gene models were filtered by removing the genes having 20% of their CDS sharing an overlap with TEs and coding region lengths <150 bp. The final gene set contained 79,668 protein-coding genes (Supplementary Tables S9, S10 and Supplementary Figs. S5, S6).

### Genome evolutionary analysis

OrthoMCL (http://orthomcl.org/orthomcl/) was used to construct orthologous gene families between *P. somniferum* (CHM and HN1) and 12 other plant species. MUSCLE^44^ was utilized to construct multiple sequence alignments of 35 single-copy orthologs among 14 species. RAxML software^45^ (version 7.2.3) was carried out to construct the maximum likelihood tree with the PROTGAMMAAUTO model by using the sequence alignments with *A. trichopoda* as an outgroup (Supplementary Fig. S9). The MCMCTree program of PAML (http://abacus.gene.ucl.ac.uk/software/paml.html) was applied to estimate divergence time using CDS alignments transformed from protein alignments. Five calibration values were chosen from the Time Tree website (http://www.timetree.org). Expansions and contractions of orthologous gene families were determined using CAFÉ 2.2 (Computational Analysis of gene Family Evolution)^46^ (Supplementary Fig. S11). For whole-genome duplication (WGD) analysis, the syntenic region between and within *P. somniferum* (Pso), *M. cordata* (Mco), and *A. coerulea* (Aco) was determined by MCscanX^47^ based on the all-to-all BLASTP results. The protein sequences of homologous gene pairs in the syntenic region were extracted and aligned using the MUSCLE program^44^. Subsequently, the protein sequence alignments were converted into CDS files, and 4DTv values were calculated based on the CDS alignments, accompanying the correction of the HKY model.

### BIA biosynthesis pathway analysis

To identify the key genes that participate in BIA biosynthetic pathways in *P. somniferum* (Pso_CHM) and other related plant species, we downloaded the protein sequences of 31 known BIA-related genes from the NCBI database (https://www.ncbi.nlm.nih.gov) as the target sequences (also see Supplemental Methods for more detail). These genes have been reportedly involved in BIA pathways and were previously cloned from Pso_HN1 with in vitro experimental validation.

We then used these genes as search queries against *P. somniferum* (Pso_CHM), *M. cordata* (Mco), *A. coerulea* (Aco), and *N. nucifera* (Nnu) using the BlastP algorithm with an *e*-value cutoff of ≤1E−10. Only blast hits with >50% identity and ≥80% coverage were retained and concatenated by Solar. The conserved domains were further identified in the retained sequences. RNA-seq reads were mapped to the CHM genome using TopHat (version 2.0.8), and DESeq was used to identify significantly differentially expressed genes.

### Correlations for BIA biosynthesis genes

Investigation of whether there were significant correlations between the expression levels of BIA pathway genes and other genes in CHM tissues was performed using Pearson’s correlation test.

### SNP and InDel identification

SNPs in CHM were detected from two methods. (1) We compared the CHM genome to the HN1 genomes using the lastz-chainnet pipeline^12^. CHM and HN1 genome alignment was performed using lastz (version 1.02.00) (https://lastz.github.io/lastz), and alignment statistics were generated with an in-house Perl script. Then, SNP sites were identified using an in-house Perl script. (2) We detected heterozygous SNPs using GATK based on alignments of short reads of HN1 onto assembled CHM genomes. Then, we located these heterozygous SNP sites on the CHM genome according to the one-to-one genome alignment results.

SV detection was carried out using lumpy (version 0.2.13, https://github.com/arq5x/lumpy-sv). First, short reads of CHM were mapped to HN1 genomes using BWA (version 0.7.8)^48^. After BWA alignment, the bam file was sorted and indexed using SAMtools (version 1.10)^49^. We then detected SV using lumpy (version 0.2.13) with default parameters.

## Supplementary information

Table S30

SUPPLEMENTAL METHODS

Figure S1

Figure S2

Figure S3

Figure S4

Figure S5

Figure S6

Figure S7

Figure S8

Figure S9

Figure S10

Figure S11

Figure S12

Figure S13

Figure S14

Figure S15

Figure S16

Figure S17

Figure S18

Figure S19

Figure S20

Table S1

Table S2

Table S3

Table S4

Table S5

Table S6

Table S7

Table S8

Table S9

Table S10

Table S11

Table S12

Table S13

Table S14

Table S15

Table S16

Table S17

Table S18

Table S19

Table S20

Table S21

Table S22

Table S23

Table S24

Table S25

Table S26

Table S27

Table S28

Table S29

## Data Availability

The raw data from our genome project were deposited in the Sequence Read Archive (SRA) database of the National Center for Biotechnology Information (NCBI) with Bioproject ID PRJNA503959. All supplementary figures and tables are provided as additional files.

## References

[1] Jacomet S (2009). Plant economy and village life in Neolithic lake dwellings at the time of the Alpine Iceman. Veg. Hist. Archaeobot..

[2] Chaturvedi N, Singh M, Shukla AK (2014). Comparative analysis of *Papaver somniferum* genotypes having contrasting latex and alkaloid profiles. Protoplasma.

[3] Facchini PJ, De Luca V (2008). Opium poppy and Madagascar periwinkle: model non‐model systems to investigate alkaloid biosynthesis in plants. Plant J..

[4] Knaul FM (2017). Alleviating the access abyss in palliative care and pain relief—an imperative of universal health coverage: the Lancet Commission report. Lancet.

[5] Beaudoin GAW, Facchini PJ (2014). Benzylisoquinoline alkaloid biosynthesis in opium poppy. Planta.

[6] Nakagawa A (2016). Total biosynthesis of opiates by stepwise fermentation using engineered Escherichia coli. Nat. Commun..

[7] Celik I (2016). Molecular genetic diversity and association mapping of morphine content and agronomic traits in Turkish opium poppy (*Papaver somniferum*) germplasm. Mol. Breed..

[8] Verma N, Jena SN, Shukla S, Yadav K (2016). Genetic diversity, population structure and marker trait associations for alkaloid content and licit opium yield in India-wide collection of poppy (*Papaver somniferum* L.). Plant Gene.

[9] Liu X (2017). The genome of medicinal plant macleaya cordata provides new insights into benzylisoquinoline alkaloids metabolism. Mol. Plant.

[10] Hori K (2018). Mining of the uncharacterized cytochrome P450 genes involved in alkaloid biosynthesis in california poppy using a draft genome sequence. Plant Cell Physiol..

[11] Guo L (2018). The opium poppy genome and morphinan production. Science.

[12] Li Y (2014). De novo assembly of soybean wild relatives for pan-genome analysis of diversity and agronomic traits. Nat. Biotechnol..

[13] Kajitani R (2014). Efficient de novo assembly of highly heterozygous genomes from whole-genome shotgun short reads. Genome Res..

[14] Burton JN (2013). Chromosome-scale scaffolding of de novo genome assemblies based on chromatin interactions. Nat. Biotechnol..

[15] Parra G, Bradnam K, Korf I (2007). CEGMA: a pipeline to accurately annotate core genes in eukaryotic genomes. Bioinformatics.

[16] Sanmiguel P, Bennetzen JL (1998). Evidence that a recent increase in maize genome size was caused by the massive amplification of intergene retrotransposons. Ann. Bot..

[17] Devos KM, Brown JKM, Bennetzen JL (2002). Genome size reduction through illegitimate recombination counteracts genome expansion in *Arabidopsis*. Genome Res..

[18] Ma J (2004). Analyses of LTR-retrotransposon structures reveal recent and rapid genomic DNA loss in rice. Genome Res..

[19] Xu YX (2014). Young but not relatively old retrotransposons are preferentially located in gene-rich euchromatic regions in tomato (*Solanum lycopersicum*) plants. Plant J..

[20] Li L, Stoeckert CJ, Roos DS (2003). OrthoMCL: identification of ortholog groups for eukaryotic genomes. Genome Res..

[21] Yang Z (1997). PAML: a program package for phylogenetic analysis by maximum likelihood. Comput. Appl. Biosci. Cabios.

[22] Yang Z (2007). PAML 4: phylogenetic analysis by maximum likelihood. Mol. Biol. Evol..

[23] Martin J (2011). The draft genome of the parasitic nematode *Trichinella spiralis*. Nat. Genet..

[24] Denoeud F (2014). The coffee genome provides insight into the convergent evolution of caffeine biosynthesis. Science.

[25] Luca VD, Pierre BS (2000). The cell and developmental biology of alkaloid biosynthesis. Trends Plant Sci..

[26] Facchini PJ, Luca VD (2010). Opium poppy and Madagascar periwinkle: model non-model systems to investigate alkaloid biosynthesis in plants. Plant J..

[27] Paterson AH (2012). Repeated polyploidization of Gossypium genomes and the evolution of spinnable cotton fibres. Nature.

[28] Morris JS (2016). Plug-and-play benzylisoquinoline alkaloid biosynthetic gene discovery in engineered yeast. Methods Enzymol..

[29] Ma C, Wang X (2012). Application of the Gini correlation coefficient to infer regulatory relationships in transcriptome analysis. Plant Physiol..

[30] Facchini PJ (1996). Molecular characterization of berberine bridge enzyme genes from opium poppy. Plant Physiol..

[31] Mortazavi A (2008). Mapping and quantifying mammalian transcriptomes by RNA-Seq. Nat. Methods.

[32] Facchini PJ, Loukanina N, Blanche V (2008). Genetic transformation via somatic embryogenesis to establish herbicide-resistant opium poppy. Plant Cell Rep..

[33] Bennetzen JL (2007). Patterns in grass genome evolution. Curr. Opin. Plant Biol..

[34] Kim S (2014). Genome sequence of the hot pepper provides insights into the evolution of pungency in Capsicum species. Nat. Genet..

[35] Bennett MD (2004). Perspectives on polyploidy in plants – ancient and neo. Biol. J. Linn. Soc..

[36] Jaillon O (2007). The grapevine genome sequence suggests ancestral hexaploidization in major angiosperm phyla. Nature.

[37] Onoyovwe A (2013). Morphine biosynthesis in opium poppy involves two cell types: sieve elements and laticifers. Plant Cell.

[38] Porebski S, Bailey LG, Baum BR (1997). Modification of a CTAB DNA extraction protocol for plants containing high polysaccharide and polyphenol components. Plant Mol. Biol. Rep..

[39] Xu Z, Wang H (2007). LTR_FINDER: an efficient tool for the prediction of full-length LTR retrotransposons. Nucleic Acids Res..

[40] Altschul SF (1990). Basic local alignment search tool. J. Mol. Biol..

[41] Trapnell C, Pachter L, Salzberg SL (2009). TopHat: discovering splice junctions with RNA-Seq. Bioinformatics.

[42] Trapnell C (2010). Transcript assembly and quantification by RNA-Seq reveals unannotated transcripts and isoform switching during cell differentiation. Nat. Biotechnol..

[43] Haas BJ (2008). Automated eukaryotic gene structure annotation using EVidenceModeler and the program to assemble spliced alignments. Genome Biol..

[44] Edgar RC (2004). MUSCLE: multiple sequence alignment with high accuracy and high throughput. Nucleic Acids Res..

[45] Stamatakis A (2006). RAxML-VI-HPC: maximum likelihood-based phylogenetic analyses with thousands of taxa and mixed models. Bioinformatics.

[46] De Bie T (2006). CAFE: a computational tool for the study of gene family evolution. Bioinformatics.

[47] Wang, Y. et al. MCScanX: a toolkit for detection and evolutionary analysis of gene synteny and collinearity. *Nucleic Acids Res.* **40**, e49 (2012). 10.1093/nar/gkr1293 PMC3326336 22217600

[48] Li H, Durbin R (2009). Fast and accurate short read alignment with Burrows–Wheeler transform. Bioinformatics.

[49] Li H (2009). The sequence alignment/map format and SAMtools. Bioinformatics.

[50] Krzywinski M (2009). Circos: an information aesthetic for comparative genomics. Genome Res..

