# Supplementary material for: Genome and transcriptome of Papaver somniferum Chinese landrace CHM indicates that massive genome expansion contributes to high benzylisoquinoline alkaloid biosynthesis
Source: Hortic Res. 2021 Jan 1;8:5. doi: 10.1038/s41438-020-00435-5 (PMC7775465; doi:10.1038/s41438-020-00435-5)
Supplement: Supplementary file 2 — SUPPLEMENTAL METHODS [file 41438_2020_435_MOESM2_ESM.doc]

**SUPPLEMENTAL METHODS**

**Genome and transcriptome of Papaver somniferum Chinese landrace CHM indicates massive genome expansion contributes to high benzylisoquinoline alkaloid biosynthesis**

Li Pei^1,†^, Baishi Wang^1,2,†^, Jian Ye^1,†^, Xiaodi Hu^3†^, Lihong Fu^2 †^, Kui Li^3,†^, Zhiyu Ni^2,4,†^ Zhenlong Wang^5^, Yujie Wei^6^, Luye Shi^5^, Ying Zhang^1^, Xue Bai^1^, Mengwan Jiang^5^, Shuhui Wang^7^, Chunling Ma^2^, Shujin Li^2^, Kaihui Liu^1^, Wanshui Li^1^, Bin Cong^2,*^

^1^ Institute of Forensic Science, Ministry of Public Security, No.17 South Muxidi Lane, Xicheng District, Beijing 100038, PR China

^2^ College of Forensic Medicine, Hebei Medical University, Hebei Key Laboratory of Forensic Medicine, Innovation Center of Forensic Medical Molecular Identification, No.361 Zhongshan East Road, Shijiazhuang, Hebei 050017, PR China

^3^ Novogene Bioinformatics Institute, Beijing, China

^4^ Hebei University, No.180 Wusidong Road, Baoding, Hebei Province, PR China

^5^ School of Life Sciences, Zhengzhou University, No.100 Science Road, Zhengzhou, Henan 450001, PR China

^6^ Gansu Academy of Agri-engineering Technology, No.234 Xinzhen Road, Huangyang town, Liangzhou District, Wuwei, Gansu 733006, PR China

^7^ Wuhan Botanical Garden, Chinese Academy of Sciences, Wuhan, 430074,

PR China

^*^Correspondence: Bin Cong, College of Forensic Medicine, Hebei Medical University, Hebei Key Laboratory of Forensic Medicine, Innovation Center of Forensic Medical Molecular Identification, No.361 Zhongshan East Road, Shijiazhuang, Hebei 050017, PR China. Email: [hbydcongbin@126.com](mailto:hbydcongbin@126.com).

^†^ These authors contributed equally to this work

**1 Genome sequencing and assembly**

## 1.1 Sample information

The DNA samples were harvested from the leaf tissues of an individual plant of *Papaver somniferum* L. (**Figure S1**), which was provided by Wuhan Botanical Garden, Chinese Academy of Sciences. High-quality genomic DNA was extracted from frozen leaf samples by a modified CTAB method ^1^. The quality and quantity of the isolated DNA were checked by electrophoresis on a 0.8% agarose gel and a NanoDrop D-1000 spectrophotometer (NanoDrop Technologies, Wilmington, DE), as well as the Qubit Fluorometer, respectively.

**1.2 Library construction and Genome sequencing**

With the qualified DNA, three types of short-insert paired-end (PE) libraries (250 bp, 300 bp, and 450 bp) were prepared using NEBNext Ultra DNA Library Prep Kit for Illumina (NEB, USA) and four types of mate-pair (MP) libraries (2 kb, 5 kb, 10 kb, and 20 kb) were prepared using Illumina’s DNA library preparation kits (TruSeq PE Cluster Kit v3, cBot, HS; and TruSeq SBS Kit v3, HS [200 cycles]) according to the manufacturer’s protocol. While the Hi-C library was also constructed using the fresh leaves according to the manufacturer’s instructions. All the libraries were sequenced on the Illumina HiSeq platform (Illumina, San Diego, CA). With the estimated genome size 3.42 Gb, The reads coverage reached over 400.10 X (**Table S1, S3**). Clean reads were obtained by removing PCR duplicate, reads containing adapters, reads containing ploy-N, and low quality reads using in-house perl scripts. For Nextera MP reads, A Nextera linker sequence (composed of the nucleotides CTGTCTCTTATACACATCTAGATGTGTATAAGAGACAG) was cut on either end of the MP.

## 1.3 Estimation of genome size

The k-mer frequency analysis is an efficient assembly-independent method for accurate estimation of genomic characteristics (genome size, repeat content and heterozygous rate, etc.).We first used about 158.20 Gb high-quality short-insert reads (350 bp fragment size) to generate 17-mer occurrence distribution using Jellyfish (version 2.1.3) and then calculated the genome size based on the formula 'Genome size=total_kmer_num/kmer_depth', where total_kmer_num is the total number of *K*-mer and kmer_depth indicates the peak position on the *K*-mer frequency distribution map(**Figure S2**). The genome size of CHM was estimated to be 3.42 Gb (**Table S2**).

## 1.4 Genome assembly and anchoring

The CHM genome was assembled using Platanus Genome Assembler (v1.2.4) ^2^. Frist, all high-quality short-insert PE reads were assembled

into contigs using Platanus assemble subprogram with parameters ‘-c 15 -k 60 -t 50 -m 300’. After obtaining contig sequences, PE reads, and MP reads were realigned onto these contigs by Platanus with scaffold subprogram (default parameters) to determine the orders of the contigs, remove bubbles and branches, and finally output scaffold sequences. GapCloser (<http://sourceforge.net/projects/>soapdenovo2/files/GapCloser) was used to close the gaps with parameters ‘-p 25 -l 150’. Finally, the 2.62 Gb genome assembly was comprised of 358,674 constructed scaffolds with the scaffold N50 size of 1.25Mbp and contig N50 size of 86.04Kbp (**Table S4**). The Hi-C clean data was aligned to the assembly using BWA software (Burrows-Wheeler Aligner, version 0.7.15, http://bio-bwa.sourceforge.net/). Only the read pairs concordantly aligned to scaffolds were used for further scaffolding to get super-scaffolds using SALSA. For super-scaffolds anchoring, nucmer (version 3.23) was used to map super-scaffolds to *P.somniferum* (HN1) genome. The chromosomal location of blocks mapped to the HN1 genome were retrieved for anchoring and orienting super-scaffolds to the corresponding chromosome. Super-scaffolds mapped to multiple chromosomes were broken at conflicted sites, and then the adjacent super-scaffolds were linked to chromosomes (87.6% of the genome) by 100 Ns (**Table 5**).

## 1.5 Genome evaluation

## 1.5.1 Reads coverage

To evaluate the integrity and accuracy of the genome assembly, all the paired

end reads were mapped to the assembly using BWA (version 0.7.15) ^3^. The mapping rate was 99.53 %, and The coverage was 99.33%. This result shows that our assembly results contain almost all of the information in reads. The GC content of CHM genome was 37.29%.

## 1.5.2 CEGMA mapping

Core Eukaryotic Genes Mapping Approach ^4^ (CEGMA, http://korflab.ucdavis.edu/dataseda/cegma/) defined a set of conserved protein families that occur in a wide range of eukaryotes, and present a mapping procedure that accurately identifies their exon-intron structures in a novel genomic sequence. Of the 248 ultra-conserved eukaryotic genes in CEGMA database, the genome assembly covered 234 complete and six partial genes (96.77%) (**Table S6**).

## 1.5.3 BUSCO mapping

Benchmarking Universal Single-Copy Orthologs (BUSCO, 4.0.5) was used to assess the completeness of gene regions of our assembly using the dataset embryophyta_odb10 (2019-11-20). BUSCO consists of single-copy orthologs derived from OrthoDB in each evolutionary lineage. Of the 1614 single-copy orthologs presented in the embryophytes, 96.4% was complete in CHM genome assemble (**Figure 1c**).

## 1.5.4 BAC evaluation

To assess the assembly base quality and genome coverage, four complete BAC sequences were downloaded from NCBI GenBank (accession numbers: JQ659012.1, MG995593.1, MG995583.1, MG995585.1. https://www.ncbi.nlm.nih.gov/) and then aligned to our assembly using LASTZ. All the BACs were entirely covered by only one scaffold.

# 2 Genome annotation

## 2.1 Annotation of repeats

Repetitive sequences consist of tandem repeats and interspersed repeats, and another name of interspersed repeats is transposable elements (TEs). The CHM TE component was annotated through a combination of *de novo* predictions and homolog-based identifications. RepeatModeler (http://www.repeatmasker.org, version1.0.5) and LTR_FINDER (version v1.05) ^5^ were used to build *de novo* repeat library, followed by RepeatMasker (http://www.repeatmasker.org,version 3.3.0) to discover TEs; TRF software (Tandem Repeats Finder, version 4.07b) ^6^ was adopted to predict tandem repeats. RepeatMasker and RepeatProteinMask were involved in homology-based identifications to detect TEs by comparing them to the Repbase. Finally, about 65.79 % of the CHM genome was identified as repetitive sequences (**Table S13**, **S14**, **S15**, and **Figure S4)**.

## 2.2 Annotation of protein-coding genes

Protein-coding genes in CHM genome were predicted using a combination of homology-based prediction, de novo prediction, and transcriptome-based prediction methods. Five *ab initio* gene prediction programs were used to predict genes, including Augustus (version 2.5.5) ^7^, Genescan (version 1.0) ^8^, Geneid ^9^, GlimmerHMM (version 3.0.2) ^10^ and SNAP (version 2013-11-29) ^11^. Protein sequences of six homologous species (*Vitis vinifera*, *Arabidopsis thaliana, Oryza sativa, Nelumbo nucifera, Aquilegia coerulea, Amborella trichopoda*) were downloaded from Ensembl or NCBI. Homologous sequences were aligned against the repeat-masked CHM genome using TBLASTN ^12^ (E-value≤ 1E-05). Genewise (version 2.2.0)^13^ was employed to predict gene models based on the alignment sequences. The RNA-seq data were mapped to the CHM genome using Tophat (version 2.0.8) ^14^ and cufflinks (version 2.1.1) ^15^ was then used to assemble the transcripts into gene models. Trinity (version 2.0.8) was used to *de novo* assemble the RNA-seq data. A weighted and non-redundant gene set was generated by EVidenceModeler (EVM) ^16^, which only keeps the longest model per locus. Then PASA software (version 2.0.2) (<http://pasapipeline.github.io/>) ^16^ improved the gene structures. Finally, gene models were filtered by removing the genes having 20% of their CDS sharing an overlap with TEs and coding region lengths less than 150 bp. The final gene set contained 79,668 protein-coding genes (**Table S9**, **S10,** and **Figure S5**, **S6**).

## 2.3 Functional annotation

Functional annotation of protein-coding genes was obtained according to best BLAST hit by BLASTP (version 2.2.26) (E-value≤ 1E-05) ^17^ against SwissProt ^18^, KEGG ^19^ (release 53) pathway and NCBI non-redundant (NR) protein databases. Gene domain annotation was carried out by using InterProScan (version 4.7) ^20^ to search against InterPro (version 29.0) ^20^ databases, including Pfam, PRINTS, PROSITE, ProDom, and SMART. A Gene Ontology (GO) ^21^ term for each gene was achieved from the corresponding InterPro descriptions. Finally, 70,857 protein-coding genes (accounting for 88.9%) owned functional annotation (**Table S11**).

## 2.4 Non-coding RNA annotation

The tRNAs were predicted by tRNAscan-SE software (version 1.3.1) ^22^ with eukaryotic parameters. The rRNAs were found by aligning to the plant rRNA database using BLASTN (version 2.2.26) (E-value ≤1E-10). The miRNA and snRNA genes were predicted using INFERNAL (version 1.1) ^23^ software by searching against the Rfam database (release 9.1) ^24^ (**Table S12**).

# 3 Genome evolutionary analysis

## 3.1 Gene family cluster

Protein sequences of *Papaver somniferum* (CHM, HN1) and other 12 species (*Atr, Amborella trichopoda; Aco, Aquilegia coerulea; Ath, Arabidopsis thaliana; Bvu, Beta vulgaris; Cca, Coffea canephora; Gma, Glycine max; Mco, Macleaya cordata; Nnu, Nelumbo nucifera; Osa, Oryza sativa; Stu, Solanum tuberosum; Tca, Theobroma cacao; Vvi, Vitis vinifera*) were filtered to select the longest sequence whose length was longer than 50 amino acids. After an all-vs-all alignments using BLASTP program (version 2.2.26) (E-value ≤ 1E-07), OrthoMCL ^25^ (http://orthomcl.org/orthomcl/) with 1.5 inflation parameter was used to construct gene families. In total, we constructed 39,926 gene families, among which 6,696 were shared among the 14 species, and 35 were single-copy orthologous gene families (**Figure S9**). Furthermore, a comparison between the two *P. somniferum* genomes revealed that 2,932 and 904 genes families were unique in the CHM and HN1 genomes, respectively (**Figure 3b**).

## 3.2 Phylogenetic tree construction and divergence time estimation

Based on the 35 single-copy orthologous gene families identified in the 14 plant species, we construct multiple sequence alignments using MUSCLE (version v3.8.31) ^26^. RAxML software (version 7.2.3) ^27^ was then carried out to construct the maximum likelihood tree with PROTGAMMAAUTO model with *A. trichopoda* as an outgroup (**Figure S9**). The mcmctree program of PAML (version 4.5) ^28^ (http://abacus.gene.ucl.ac.uk/software/paml.html) was applied to estimate divergence time. Eight calibration values were chosen from Time Tree website (http://www.timetree.org) as 107-109Mya for TMRCA of *A. thaliana-G. max*; 113-114Mya of *A. thaliana-V. vinifera*; 107-125Mya of *A. thaliana-C. canephora*; 122-134Mya of *A. thaliana-M. cordata*; 148-173Mya of *A. thaliana-O. sativa*. CHM and HN1 diverged approximately at 1.5Mya (**Figure 3a**).

## 3.3 Gene families expand and contract

Expansion and contractions of gene families were determined using CAFÉ 2.2 (Computational Analysis of gene Family Evolution) ^29^ (**Figure S10**). The program uses a birth and death process to model gene gain and loss over a phylogeny. Large changes in gene family size in a phylogeny were tested by calculating p-values on each branch using the Viterbi method with a randomly generated likelihood distribution. This method calculates exact p-values for transitions between the parent and child family sizes for all branches of the phylogenetic tree. Enrichment of Gene Ontology (FDR<0.05) terms for opium poppy expanded and contracted gene families were calculated.

## 3.4 Repetitive nature and genome expansion

Compared with the *M. cordata*, *E. californica* genomes, *P. somniferum* (CHM and HN1) have huge genomes, and Long terminal repeat (LTR) retrotransposons represent the major parts of the genomes (**Figure S7**).

Unequal intra-element homologous recombination (UIHR), which produces solo LTRs, is considered one of the major processes leading to the removal of LTR-RT DNA in plants ^30,31^. With the increasing number of UIHR events, the ratio of solo LTRs to intact elements (S/I ratios) should increase over time. The intact elements missed by the program of LTR-STRUC ^32^ were identified as solo LTRs by methods previously described ^31,33^ for *M. cordat*, *E. californica*and *P. somniferum* (CHM and HN1). The result was showed in table S16.

## 3.5 Whole genome duplication (WGD) of *P. somniferum*

The sequences alignment result was obtained by all-to-all blastP ^17^ analysis between protein sequences from CHM (Pso), *M. cordata* (Mco), *A. coerulea* (Aco), as well as within each species, employing the parameters of e-value≤1e-10, coverage ≥50% and identity≥20%. Then the syntenic region between and within these species was carried by MCscanX ^34^. The protein sequences of gene pairs in the syntenic region were extracted, and alignment using the MUSCLE program ^17^. Subsequently, the protein sequences alignments were converted into CDS alignments based on the coding sequences, and 4DTV values were then calculated, accompanying the correction of HKY model.

The evolution rate of Mco is slower than *P. somniferum* based on the branch lengths of the phylogenetic tree. Comparison of 4DTv values of *M. cordata*–*A. coerulea* with *P. somniferum*–*A. coerulea* showed that the average value is 24.5% lower, which also suggested that the evolutionary rate of *M. cordata* is much slower than that of *P. somniferum* . We thus adjusted the 4DTv distributions of *M. cordata*–*M. cordata* accordingly, and found that the paralog peaks of *M. cordata*, *P. somniferum,* and *A. coerulea* occupied almost the same position.

# 4 BIAs biosynthesis pathway analysis

## 4.1 Identification of genes involving in BIAs biosynthesis pathway analysis

To identify the key genes that participate in BIA biosynthetic pathways in *P. somniferum* (Pso_CHM) and other related plant species, we downloaded the protein sequences of 31 known BIA-related genes from NCBI database (https://www.ncbi.nlm.nih.gov) as the target sequences. These genes have been reportedly involved in BIA pathways and were previously cloned from Pso_HN1 with in vitro experimental validation. We then used these genes as search queries against *P. somniferum* (Pso_CHM), *M. cordata* (Mco), *A. coerulea* (Aco), and *N. nucifera* (Nnu) using the BlastP algorithm with an e-value cutoff of ≤ 1E-10. Only blast hits with > 50% identity and ≥ 80% coverage were retained and concatenated by Solar. The conserved domains were further identified in the retained sequences (**Table S24**). RNA-seq reads were mapped to the CHM genome using TopHat (version 2.0.8), and DESeq was used to identify significantly differentially expressed genes.

**4.2 Transcriptome sequencing**

Seeds, stems, and different developmental stages of leaves and fruits were collected from different plants of CHM in Wuhan Botanical Garden, Chinese Academy of Sciences. Total RNAs were isolated using a modified CTAB method and then treated with RNase-free DNase I (Promega, USA). RNA-Seq libraries were constructed using the NEB Next UltraTM RNA Library Prep Kit (NEB, USA) and sequenced on a HiSeqX. RNA-seq reads were mapped to the CHM genome using TopHat (version 2.0.8). The total numbers of aligned reads (read counts) for each gene were normalized to the reads per kilobase exon model per million mapped reads (RPKM). DESeq was used to identify differentially expressed genes.

# 5 The potential genes Identification

In order to predict 3'OHase and 3'OMT, 58 candidate genes containing the PF08100 (Dimerisation): Dimerisation domain and PF00891 (Methyltransf_2): O-methyltransferase, were first selected. We then filtered out 14 genes due to their previous identification as components of BIA biosynthesis pathways, and 44 genes were retained for further correlation analysis. Investigation of whether there were significant correlations between expression levels of these 44 genes and BIA pathway genes in CHM tissues was performed using the Pearson’s correlation test (**Figure S23**). Finally, we get 13 candidate genes (Pearson’s correlation test, r>=0.9). Based on the three BBE genes that have been identified (Red marked), we further explored potential BBE genes that harbor the PF08031 (BBE) and PF01565 (FAD_binding_4) domains (Pearson’s correlation test, r>=0.9) (**Figure S24**).

# 6 SNP and indel identification.

SNPs in CHM were detected from two methods. 1. We compared the CHM genome to the HN1 genome using the lastz-chainnet pipeline ^35,36^. CHM and HN1 genomes alignment was done using lastz (version 1.02.00) (https://lastz.github.io/lastz), and alignment result statistics was done with in-house perl script.T hen SNP sites were picked out by in-house perl script. 2. We detected heterozygous SNPs using GATK (v3.7) based on alignments of short reads of HN1 onto assembled CHM genomes. Then we located these heterozygous SNP sites on CHM genome according to the one-to-one genome alignment results.

SV detection was done using lumpy (version 0.2.13 https://github.com/arq5x/lumpy-sv). First, short reads of CHM were mapped to HN1 genomes using BWA (version 0.7.8) ^37^. After BWA alignment, the bam file was sorted and indexed using SAMtools (version 1.10)^38^. We then detected SV using lumpy (version 0.2.13) with default parameters.

**REFERENCES**

1 Porebski, S., Bailey, L. G. & Baum, B. R. Modification of a CTAB DNA extraction protocol for plants containing high polysaccharide and polyphenol components. *Plant Molecular Biology Reporter* **15**, 8-15 (1997).

2 Kajitani, R. *et al.* Efficient de novo assembly of highly heterozygous genomes from whole-genome shotgun short reads. *Genome Research* **24**, 1384-1395 (2014).

3 Li, H. Aligning sequence reads, clone sequences and assembly contigs with BWA-MEM. *arXiv: Genomics* (2013).

4 Parra, G., Bradnam, K. & Korf, I. F. CEGMA: a pipeline to accurately annotate core genes in eukaryotic genomes. *Bioinformatics* **23**, 1061-1067 (2007).

5 Xu, Z. & Wang, H. LTR_FINDER: an efficient tool for the prediction of full-length LTR retrotransposons. *Nucleic Acids Res* **35**, W265-268, doi:10.1093/nar/gkm286 (2007).

6 Benson, G. Tandem repeats finder: a program to analyze DNA sequences. *Nucleic Acids Research* **27**, 573-580 (1999).

7 Stanke, M., Steinkamp, R., Waack, S. & Morgenstern, B. AUGUSTUS: a web server for gene finding in eukaryotes. *Nucleic Acids Research* **32**, 309-312 (2004).

8 Burge, C. B. & Karlin, S. Prediction of Complete Gene Structures in Human Genomic DNA. *Journal of Molecular Biology* **268**, 78-94 (1997).

9 Guigo, R. Assembling genes from predicted exons in linear time with dynamic programming. *Journal of Computational Biology* **5**, 681-702 (1998).

10 Majoros, W. H., Pertea, M. & Salzberg, S. L. TigrScan and GlimmerHMM: two open source ab initio eukaryotic gene-finders. *Bioinformatics* **20**, 2878-2879 (2004).

11 Korf, I. F. Gene finding in novel genomes. *BMC Bioinformatics* **5**, 59-59 (2004).

12 Altschul, S. F., Gish, W., Miller, W., Myers, E. W. & Lipman, D. J. Basic Local Alignment Search Tool. *Journal of Molecular Biology* **215**, 403-410 (1990).

13 Birney, E., Clamp, M. & Durbin, R. GeneWise and Genomewise. *Genome Research* **14**, 988-995 (2004).

14 Trapnell, C., Pachter, L. & Salzberg, S. L. TopHat: discovering splice junctions with RNA-Seq. *Bioinformatics* **25**, 1105-1111 (2009).

15 Trapnell, C. *et al.* Transcript assembly and quantification by RNA-Seq reveals unannotated transcripts and isoform switching during cell differentiation. *Nature Biotechnology* **28**, 511-515 (2010).

16 Haas, B. J. *et al.* Automated eukaryotic gene structure annotation using EVidenceModeler and the Program to Assemble Spliced Alignments. *Genome Biology* **9**, 1-22 (2008).

17 Altschul, S. F. *et al.* Gapped BLAST and PSI-BLAST: a new generation of protein database search programs. *Nucleic Acids Research* **25**, 3389-3402 (1997).

18 Bairoch, A. M. & Apweiler, R. The SWISS-PROT protein sequence data bank and its supplement TrEMBL in 1999. *Nucleic Acids Research* **27**, 38-42 (1998).

19 Kanehisa, M. *et al.* Data, information, knowledge and principle: back to metabolism in KEGG. *Nucleic Acids Research* **42**, 199-205 (2014).

20 Hunter, S. *et al.* InterPro in 2011: new developments in the family and domain prediction database. *Nucleic Acids Research* **40**, 4725-4725 (2012).

21 Ashburner, M. *et al.* Gene ontology: tool for the unification of biology. The Gene Ontology Consortium. *Nature Genetics* **25**, 25-29 (2000).

22 Lowe, T. M. & Eddy, S. R. tRNAscan-SE: a program for improved detection of transfer RNA genes in genomic sequence. *Nucleic Acids Research* **25**, 955-964 (1997).

23 Nawrocki, E. P., Kolbe, D. L. & Eddy, S. R. Infernal 1.0: inference of RNA alignments. *Bioinformatics* **25**, 1335-1337 (2009).

24 Griffithsjones, S. *et al.* Rfam: annotating non-coding RNAs in complete genomes. *Nucleic Acids Research* **33**, 121-124 (2004).

25 Li, L., Stoeckert, C. J. & Roos, D. S. OrthoMCL: Identification of Ortholog Groups for Eukaryotic Genomes. *Genome Research* **13**, 2178-2189 (2003).

26 Edgar, R. C. MUSCLE: multiple sequence alignment with high accuracy and high throughput. *Nucleic Acids Research* **32**, 1792-1797 (2004).

27 Stamatakis, A. RAxML-VI-HPC: maximum likelihood-based phylogenetic analyses with thousands of taxa and mixed models. *Bioinformatics* **22**, 2688-2690 (2006).

28 Yang, Z. PAML 4: Phylogenetic Analysis by Maximum Likelihood. *Molecular Biology and Evolution* **24**, 1586-1591 (2007).

29 De Bie, T., Cristianini, N., Demuth, J. P. & Hahn, M. W. CAFE: a computational tool for the study of gene family evolution. *Bioinformatics* **22**, 1269-1271 (2006).

30 Devos, K. M., Brown, J. K. M. & Bennetzen, J. L. Genome Size Reduction through Illegitimate Recombination Counteracts Genome Expansion in Arabidopsis. *Genome Research* **12**, 1075-1079 (2002).

31 Ma, J., Devos, K. M. & Bennetzen, J. L. Analyses of LTR-Retrotransposon Structures Reveal Recent and Rapid Genomic DNA Loss in Rice. *Genome Research* **14**, 860-869 (2004).

32 McCarthy, E. M. & McDonald, J. F. LTR_STRUC: a novel search and identification program for LTR retrotransposons. *Bioinformatics* **19**, 362-367, doi:10.1093/bioinformatics/btf878 (2003).

33 Ma, J. & Bennetzen, J. L. Rapid recent growth and divergence of rice nuclear genomes. *Proceedings of the National Academy of Sciences of the United States of America* **101**, 12404-12410, doi:10.1073/pnas.0403715101 (2004).

34 Wang, Y. *et al.* MCScanX: a toolkit for detection and evolutionary analysis of gene synteny and collinearity. *Nucleic Acids Research* **40** (2012).

35 Li, Y. *et al.* De novo assembly of soybean wild relatives for pan-genome analysis of diversity and agronomic traits. *Nature Biotechnology* **32**, 1045-1052 (2014).

36 Harris, R. S.

37 Li, H. & Durbin, R. Fast and accurate short read alignment with Burrows–Wheeler transform. *Bioinformatics* **25**, 1754-1760 (2009).

38 Li, H. *et al.* The Sequence Alignment/Map format and SAMtools. *Bioinformatics* **25**, 2078-2079 (2009).
