# Supplementary figures and images for: Genome and transcriptome of Papaver somniferum Chinese landrace CHM indicates that massive genome expansion contributes to high benzylisoquinoline alkaloid biosynthesis
Source: Hortic Res. 2021 Jan 1;8:5. doi: 10.1038/s41438-020-00435-5 (PMC7775465; doi:10.1038/s41438-020-00435-5)

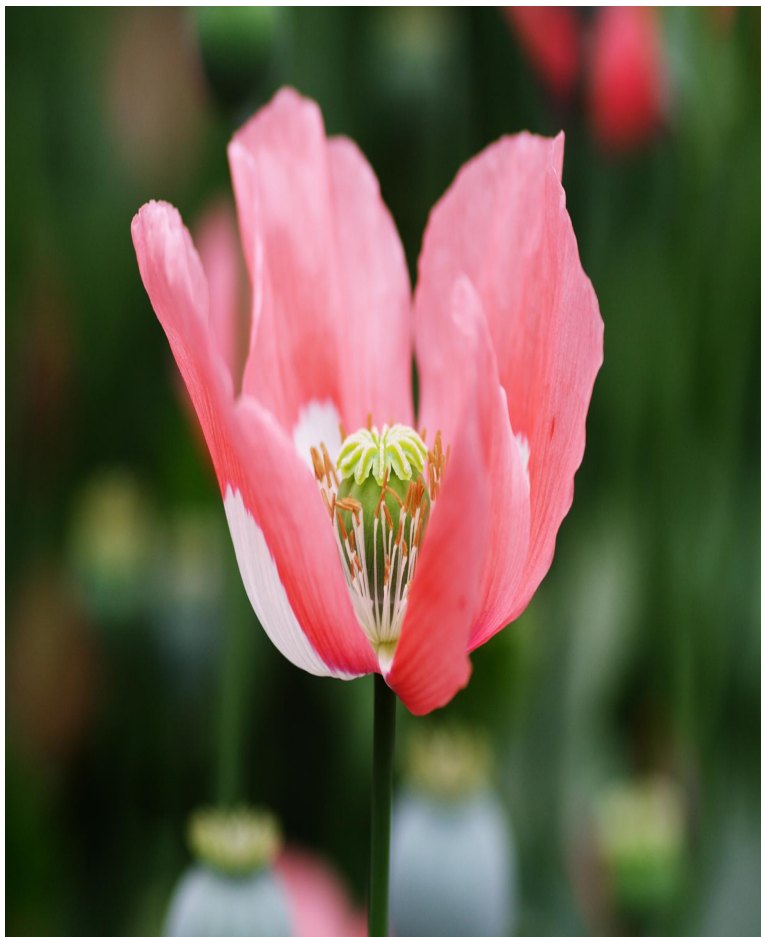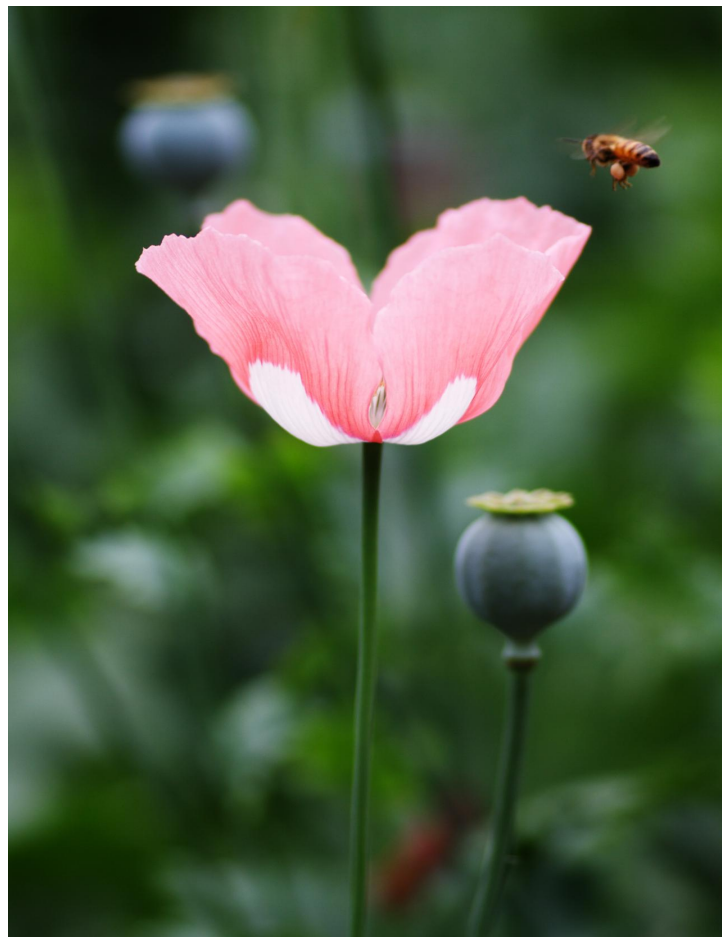

Supplement: Supplementary file 3 — Figure S1 [file 41438_2020_435_MOESM3_ESM.pdf]

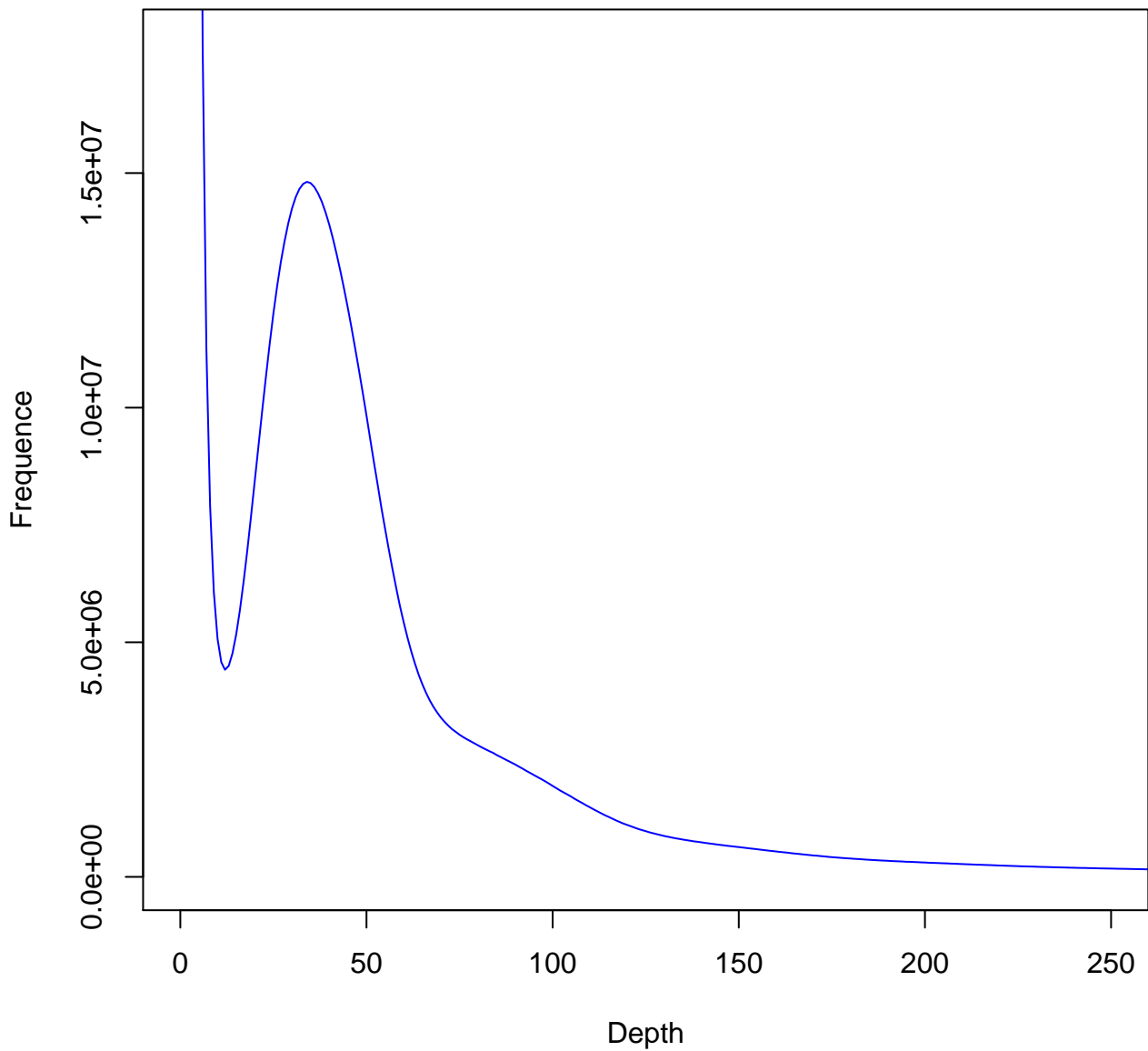

Supplement: Supplementary file 4 — Figure S2 [file 41438_2020_435_MOESM4_ESM.pdf]

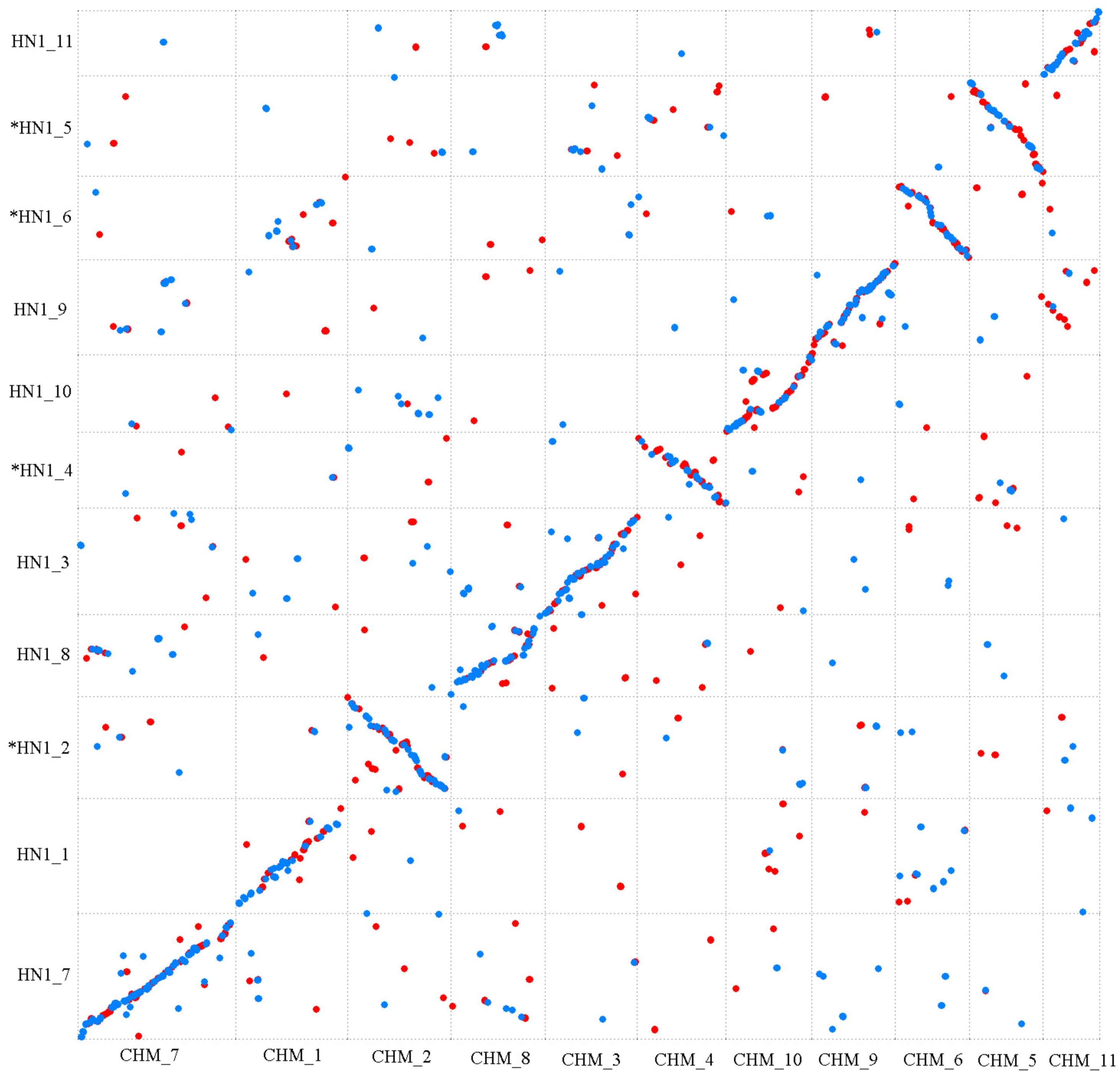

Supplement: Supplementary file 5 — Figure S3 [file 41438_2020_435_MOESM5_ESM.pdf]

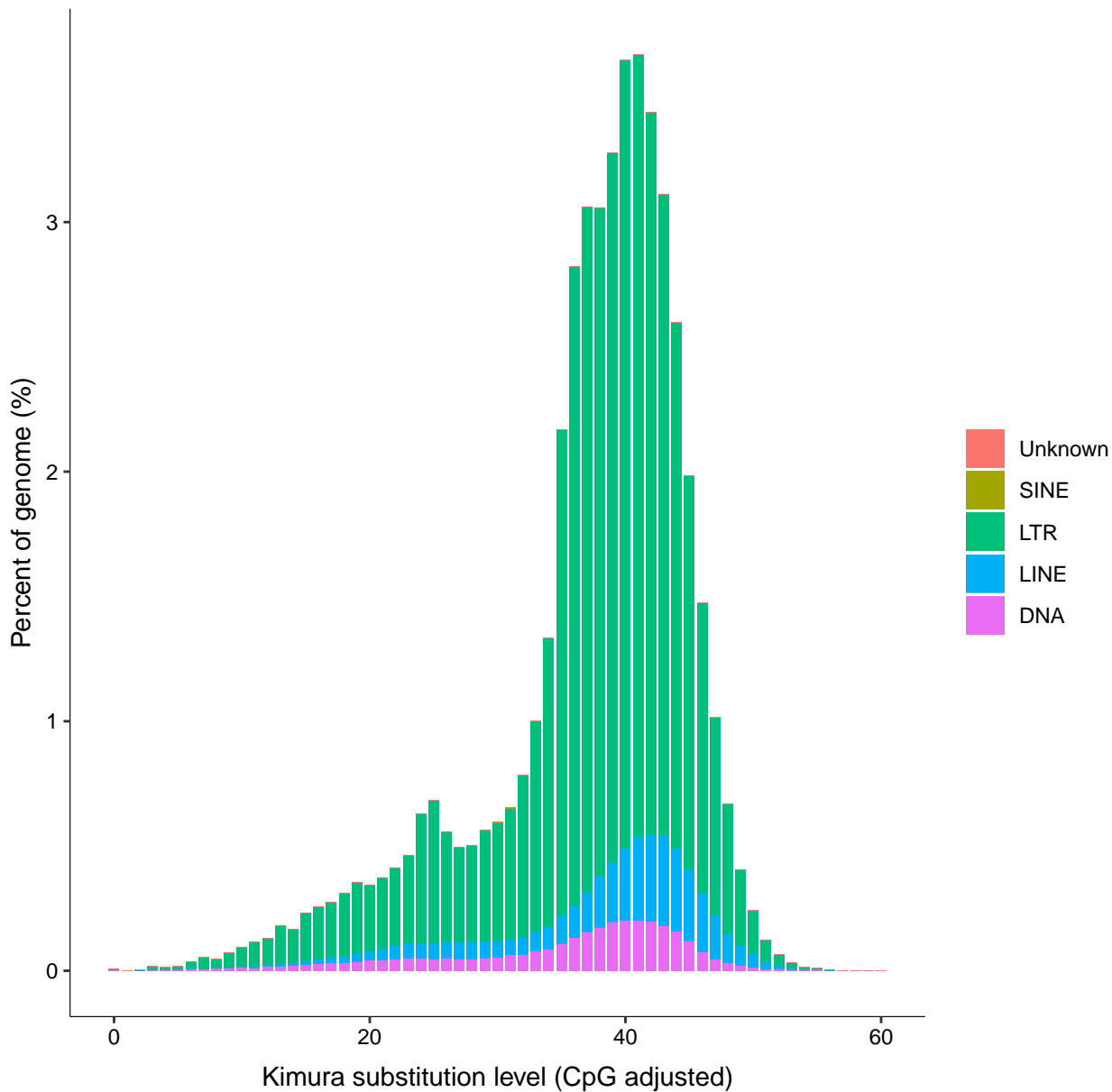

Supplement: Supplementary file 6 — Figure S4 [file 41438_2020_435_MOESM6_ESM.pdf]

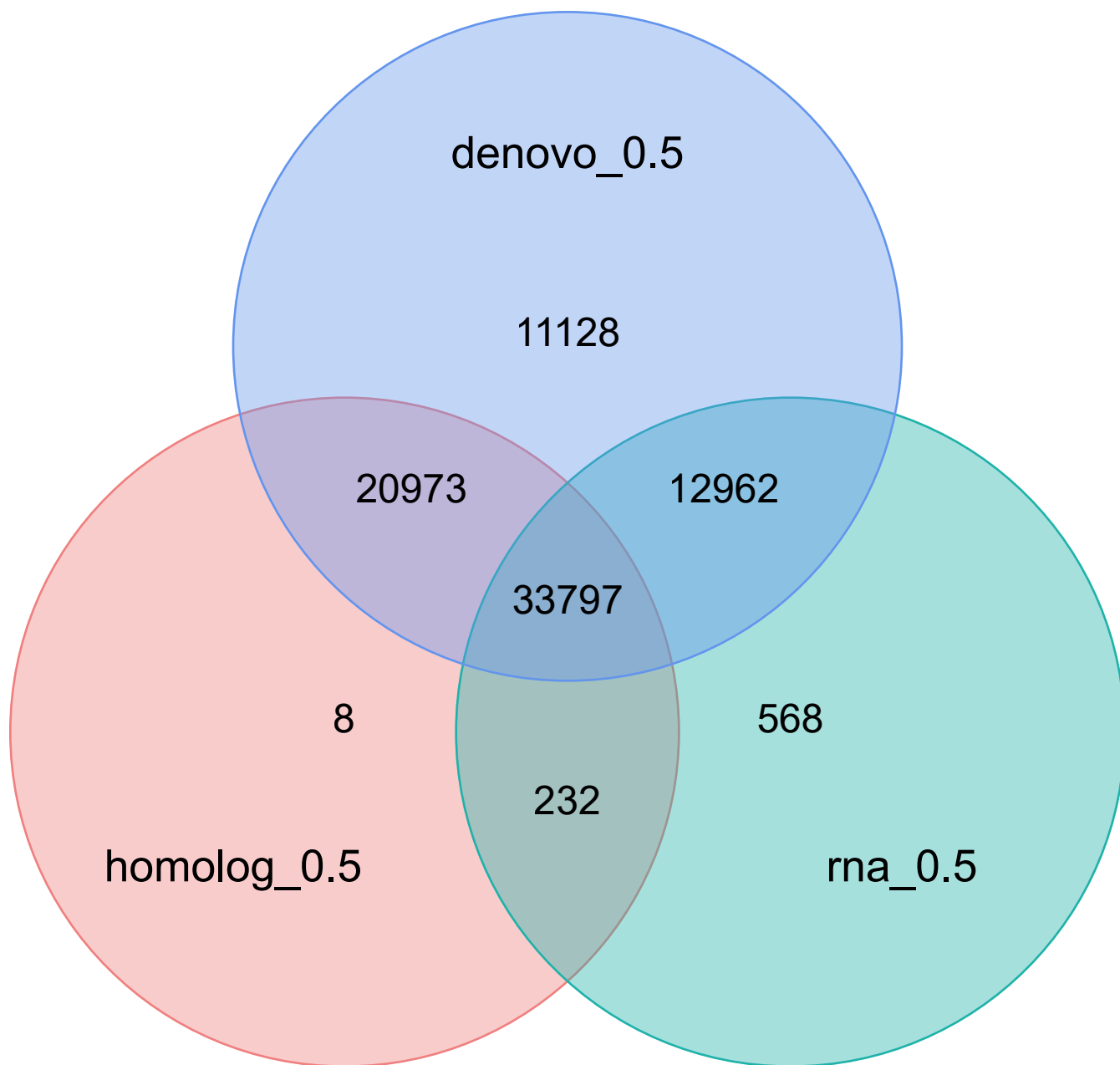

Evidence Support

Supplement: Supplementary file 8 — Figure S6 [file 41438_2020_435_MOESM8_ESM.pdf]

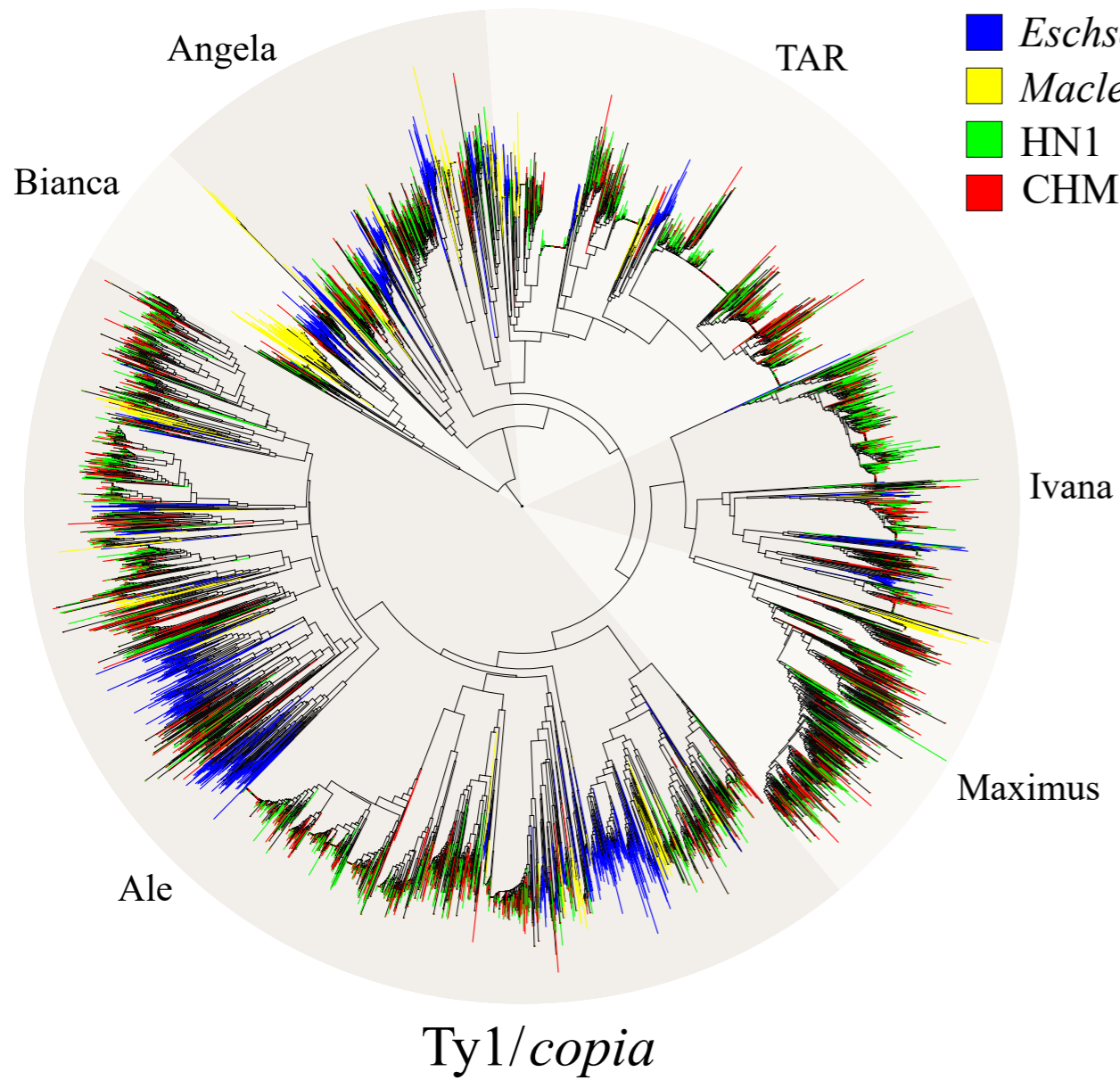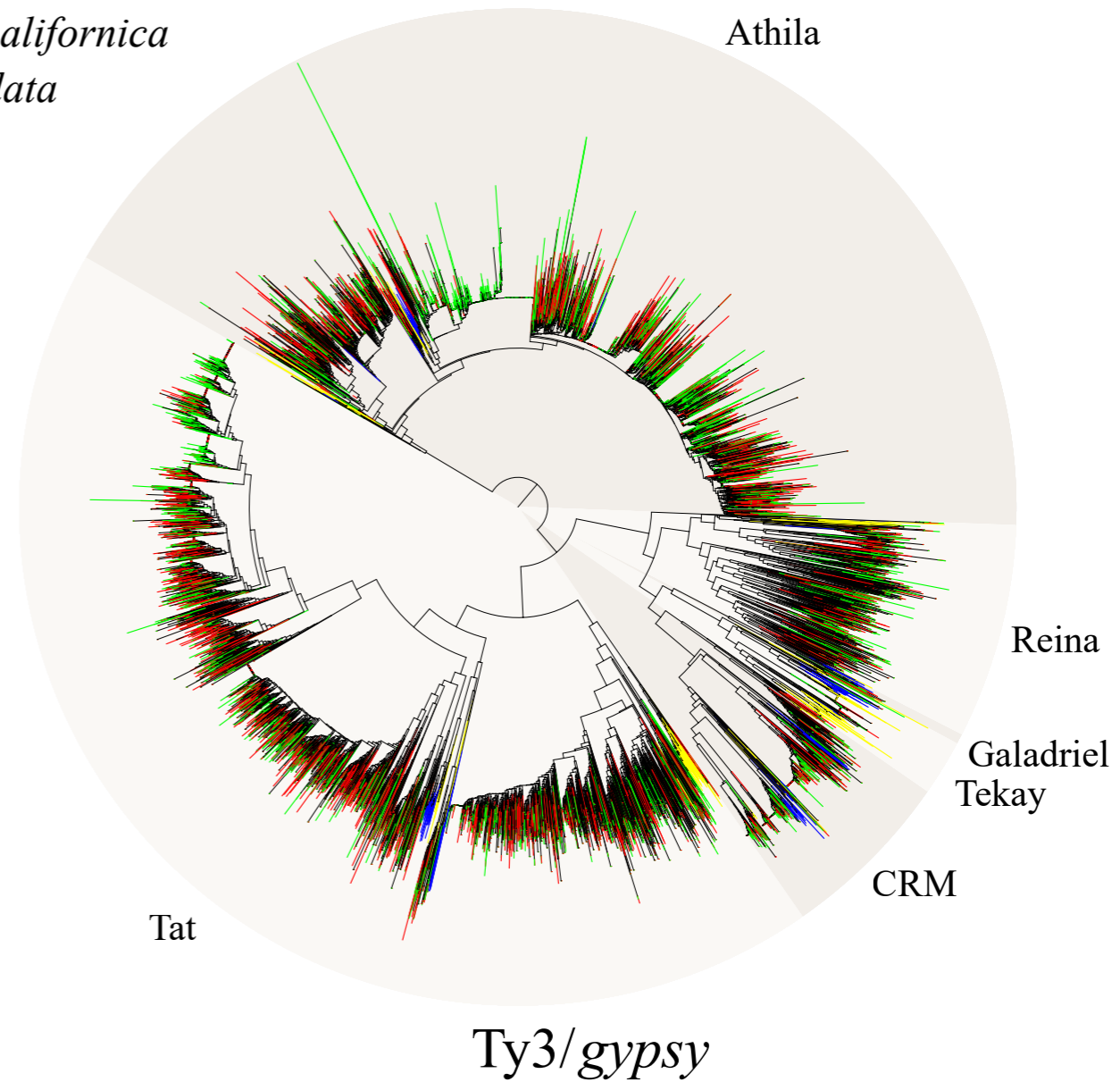

Supplement: Supplementary file 9 — Figure S7 [file 41438_2020_435_MOESM9_ESM.pdf]

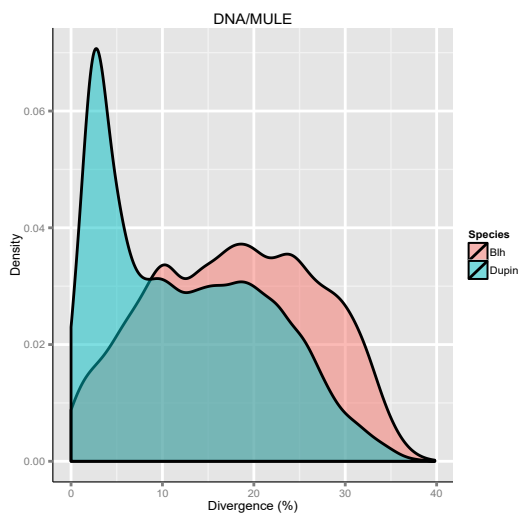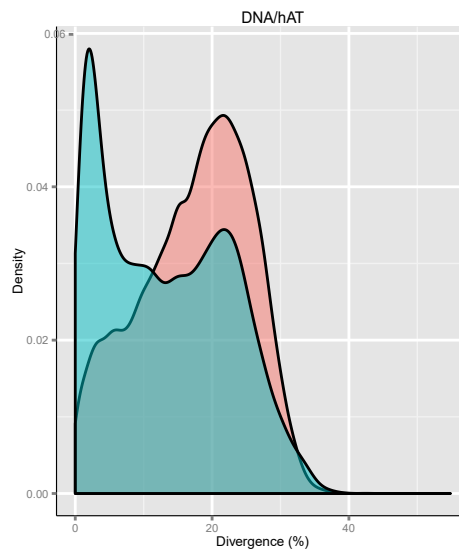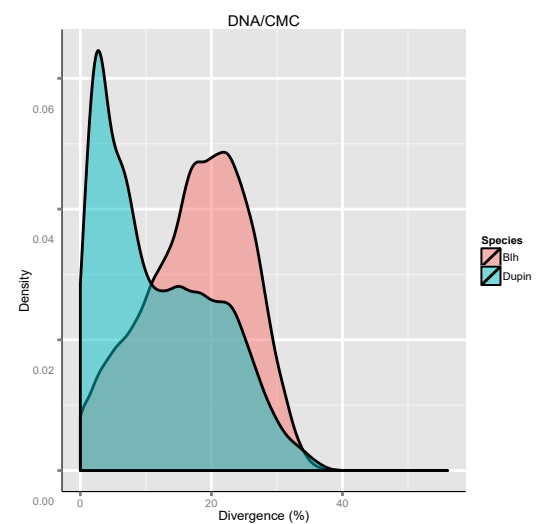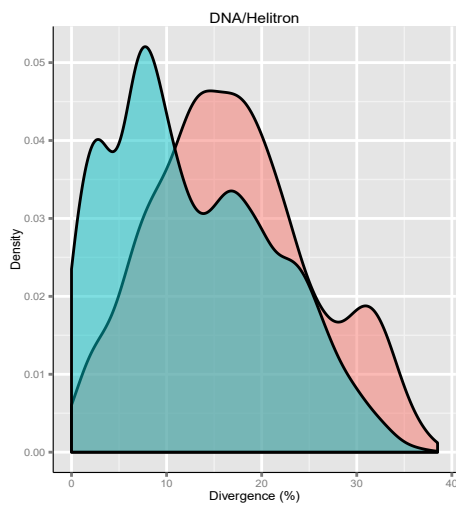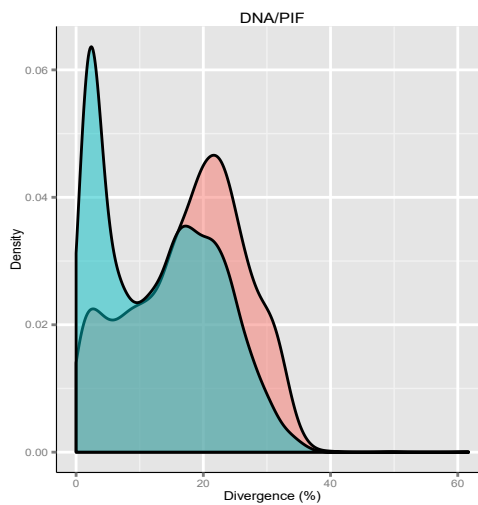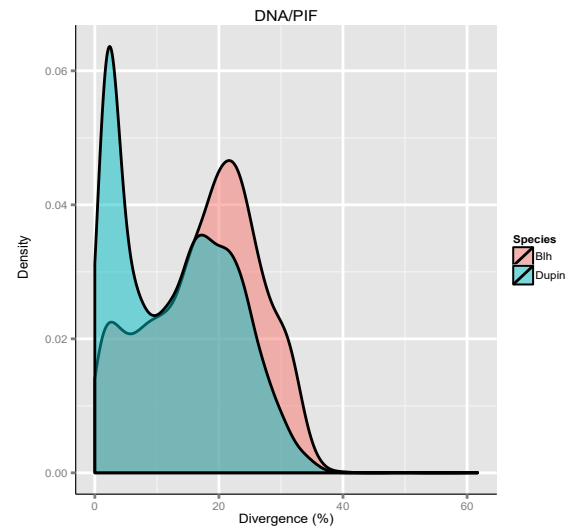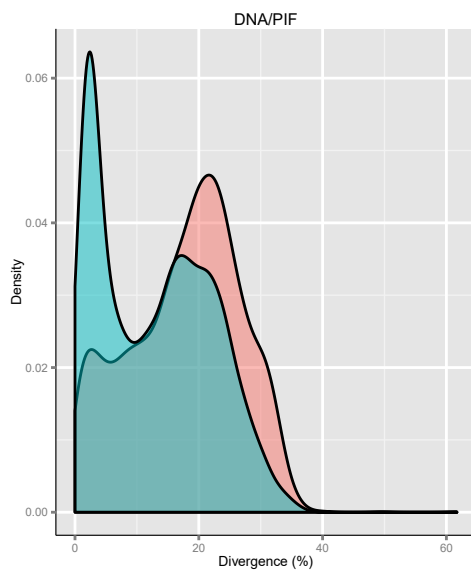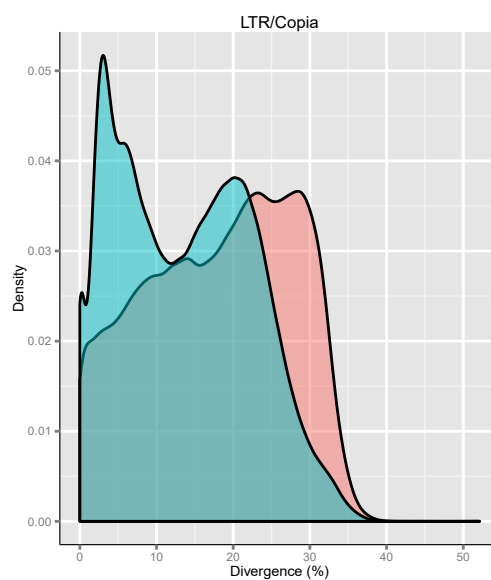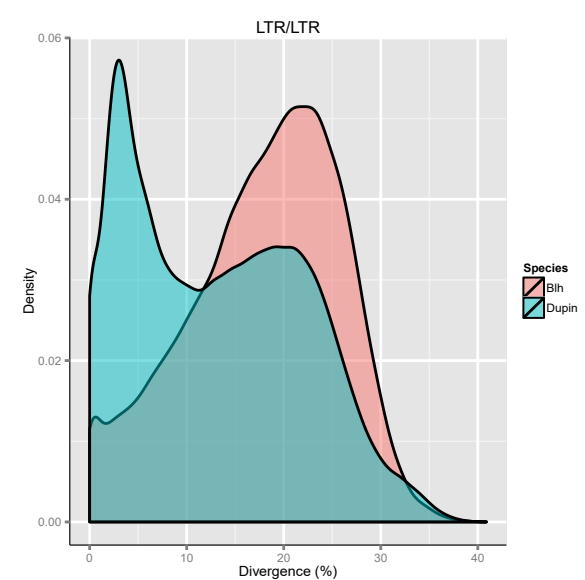

Supplement: Supplementary file 10 — Figure S8 [file 41438_2020_435_MOESM10_ESM.pdf]

A

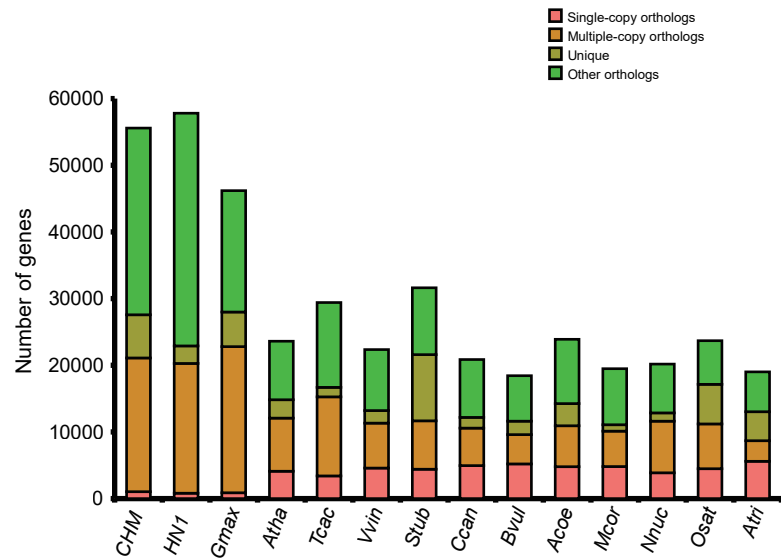

B

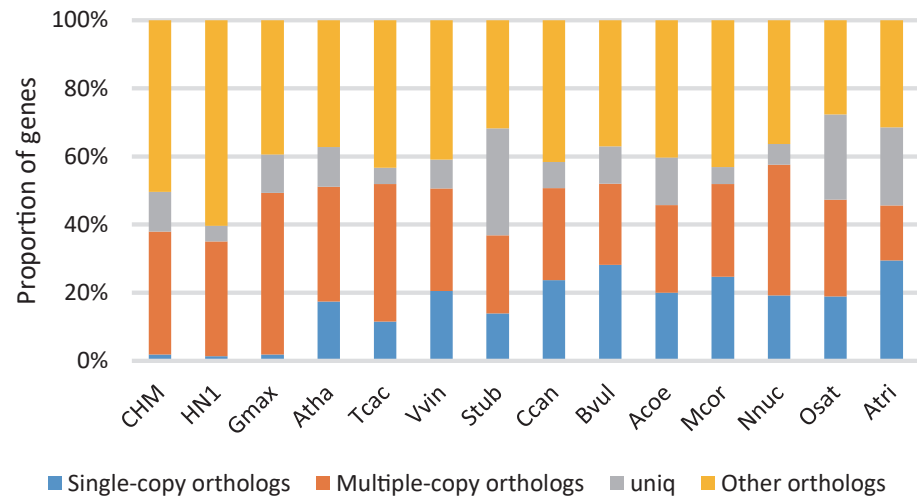

Supplement: Supplementary file 11 — Figure S9 [file 41438_2020_435_MOESM11_ESM.pdf]

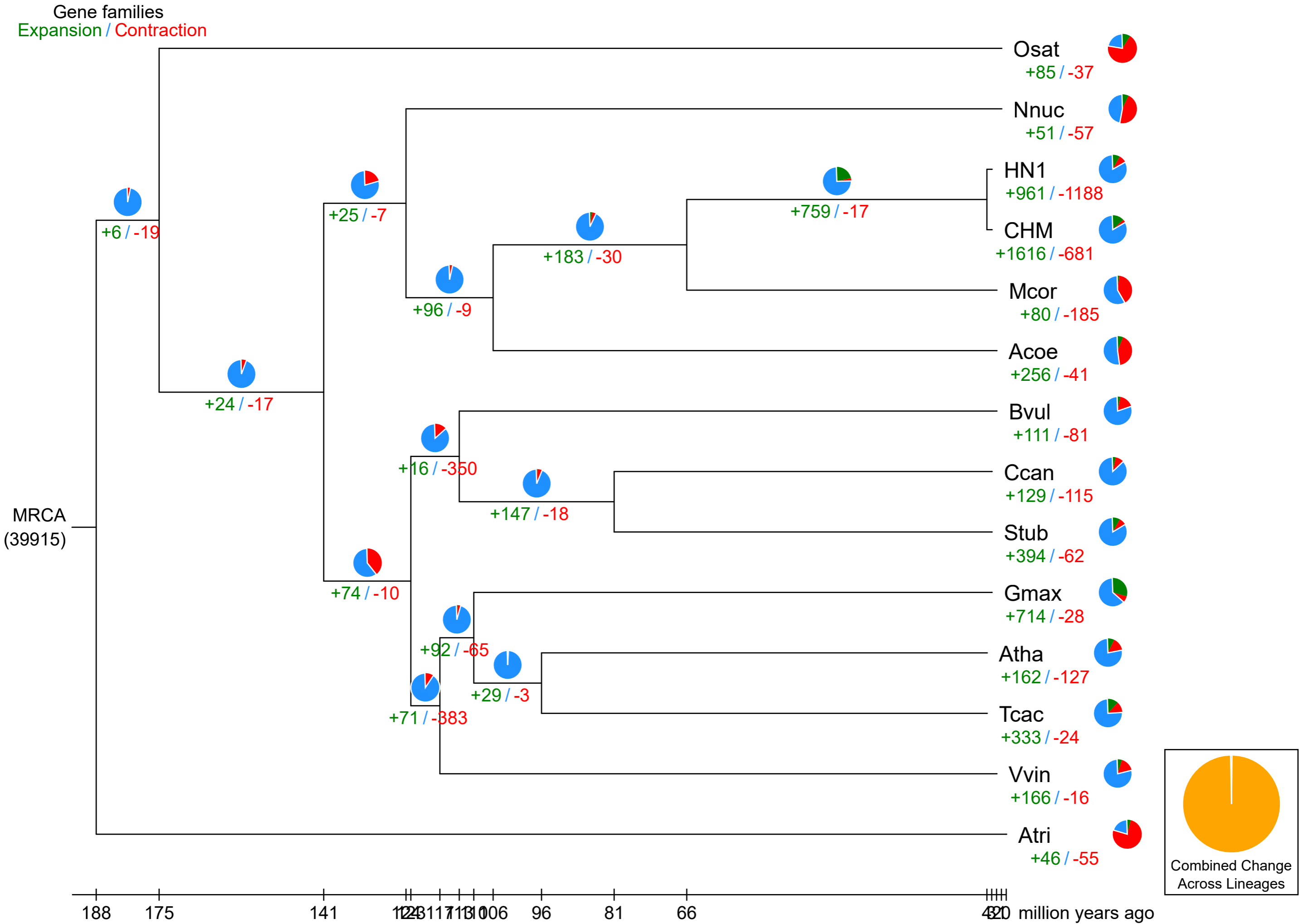

Supplement: Supplementary file 12 — Figure S10 [file 41438_2020_435_MOESM12_ESM.pdf]

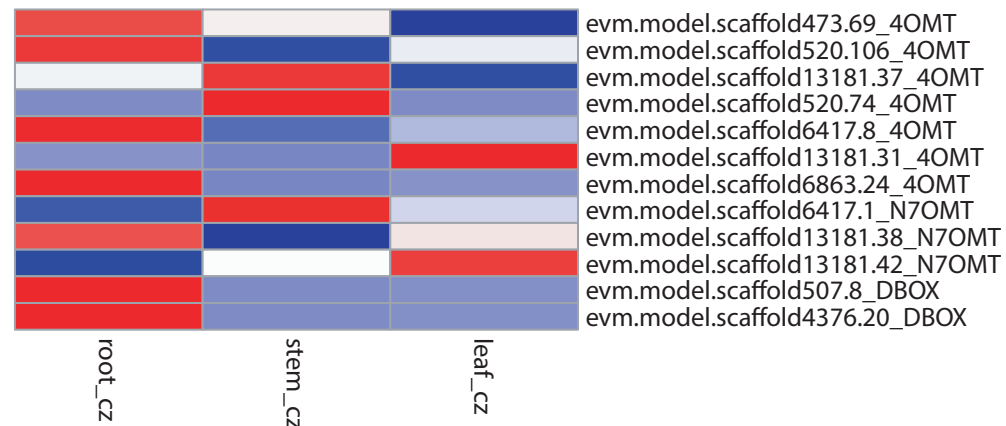

A

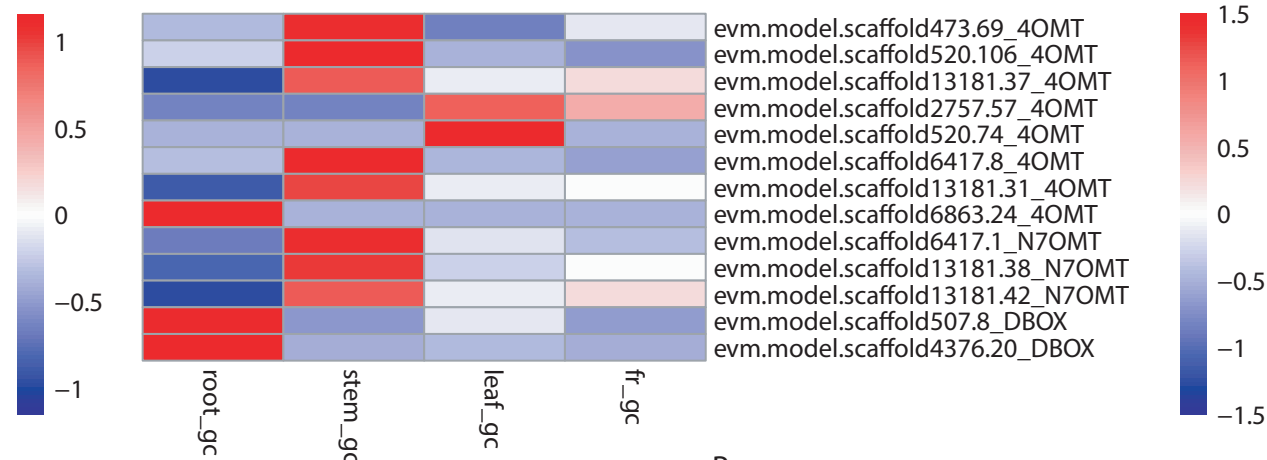

B

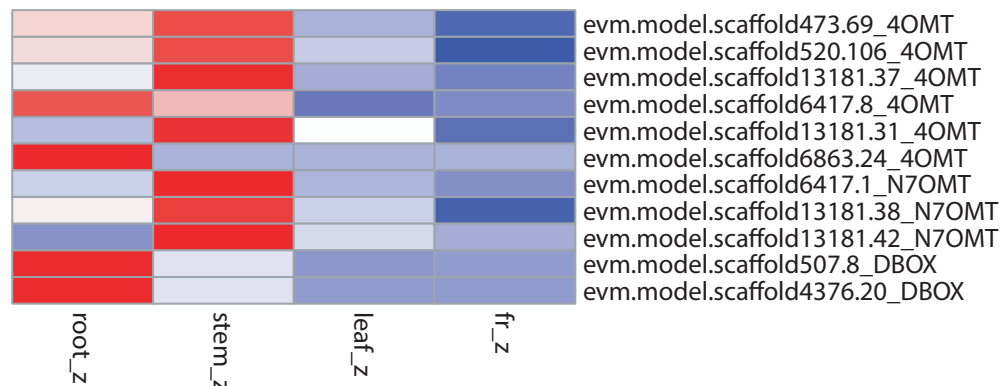

C

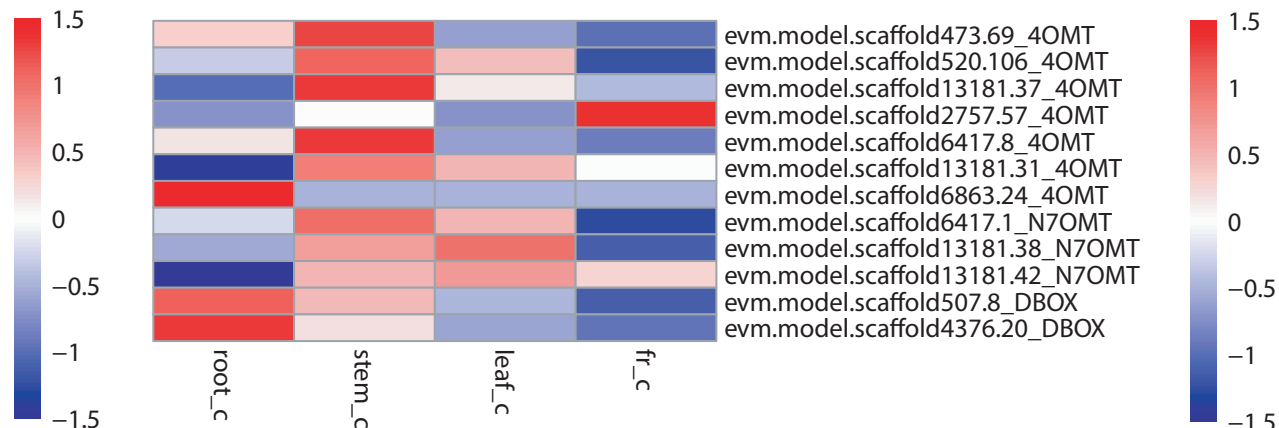

D

Supplement: Supplementary file 13 — Figure S11 [file 41438_2020_435_MOESM13_ESM.pdf]

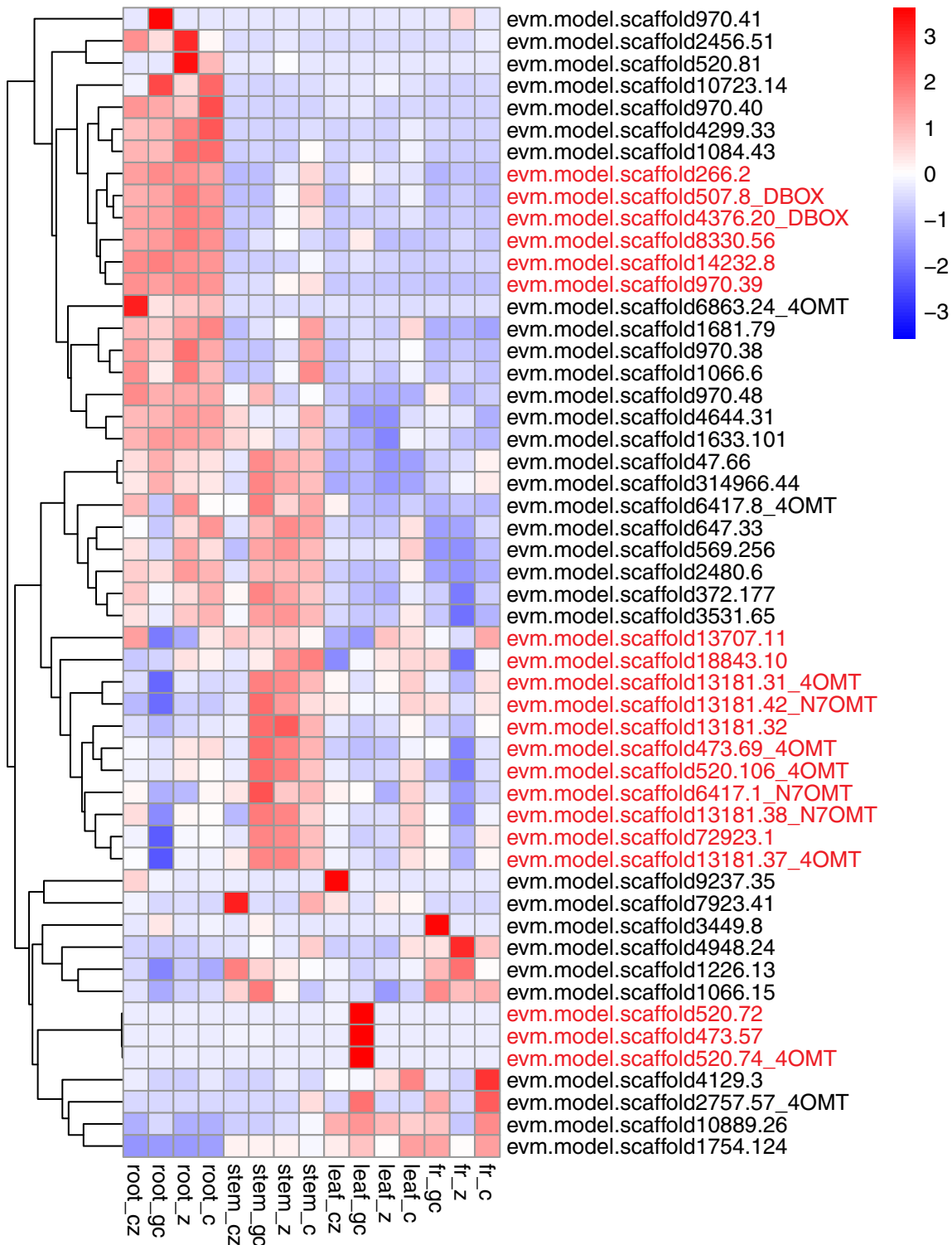

Supplement: Supplementary file 14 — Figure S12 [file 41438_2020_435_MOESM14_ESM.pdf]

A

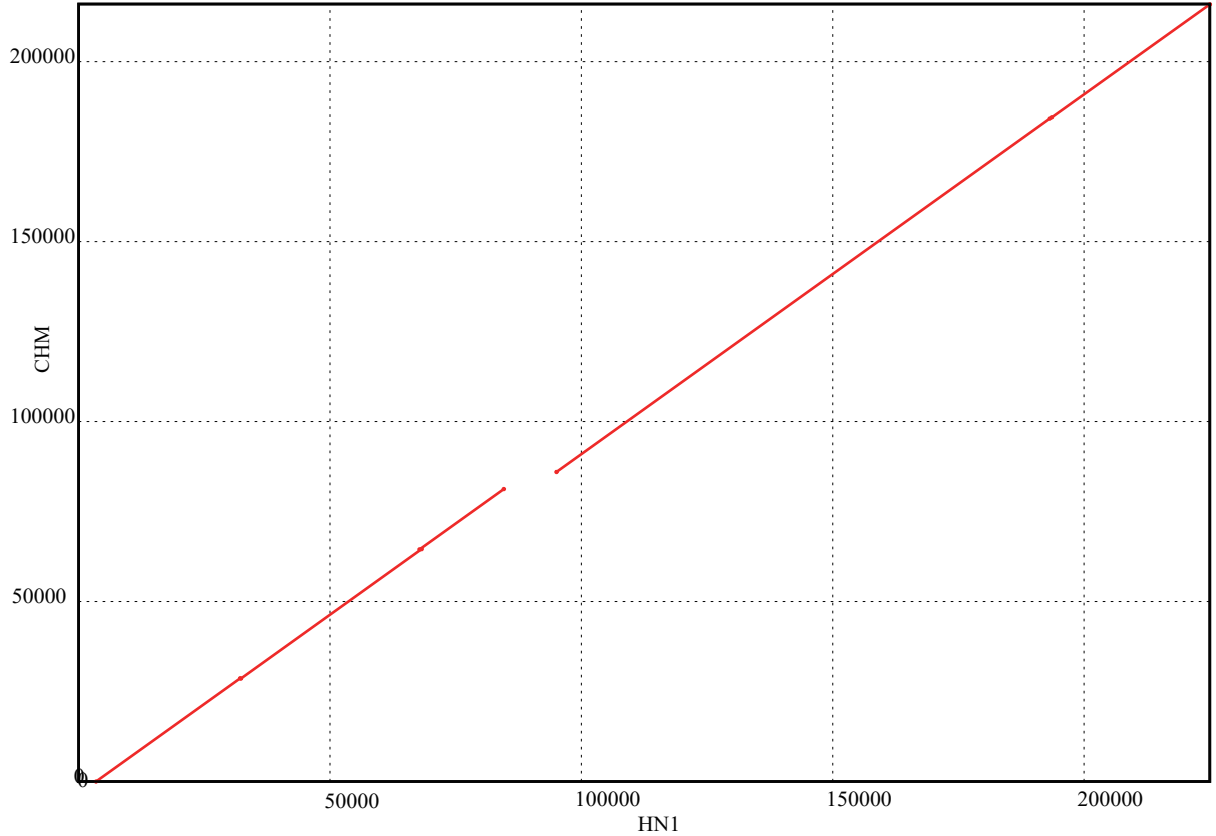

B

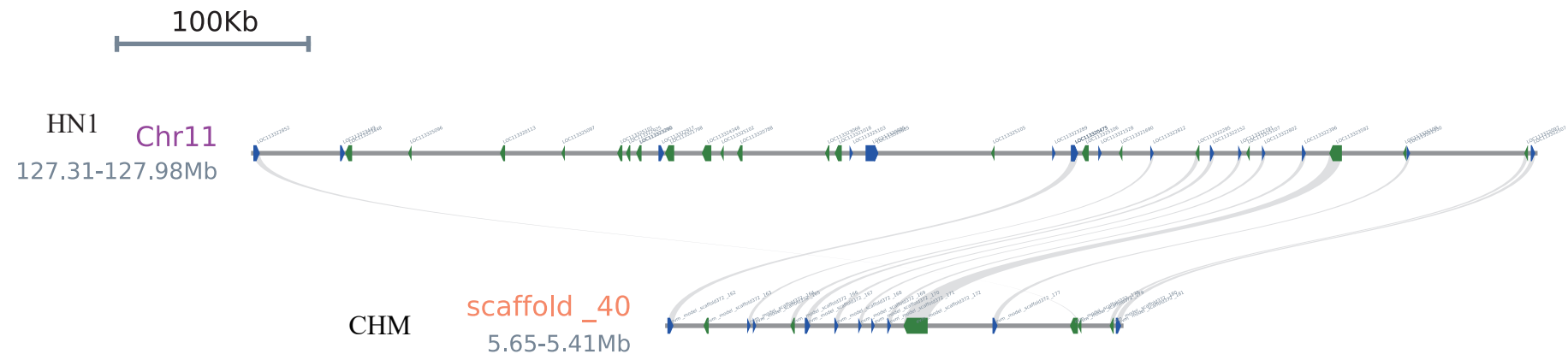

Supplement: Supplementary file 15 — Figure S13 [file 41438_2020_435_MOESM15_ESM.pdf]

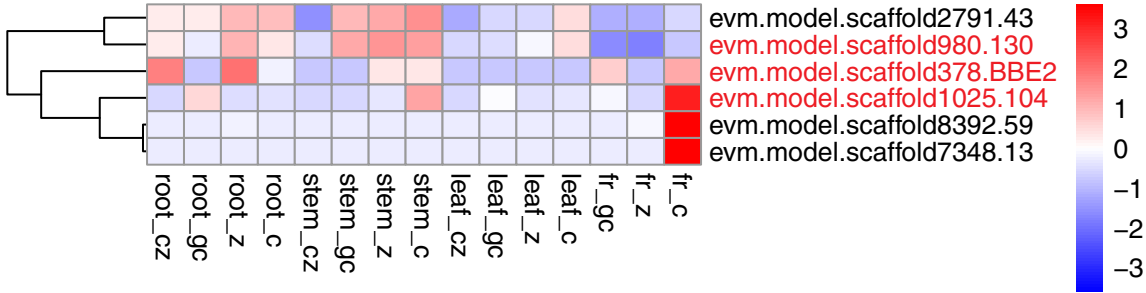

Supplement: Supplementary file 16 — Figure S14 [file 41438_2020_435_MOESM16_ESM.pdf]

Cluster analysis of differentially expressed genes

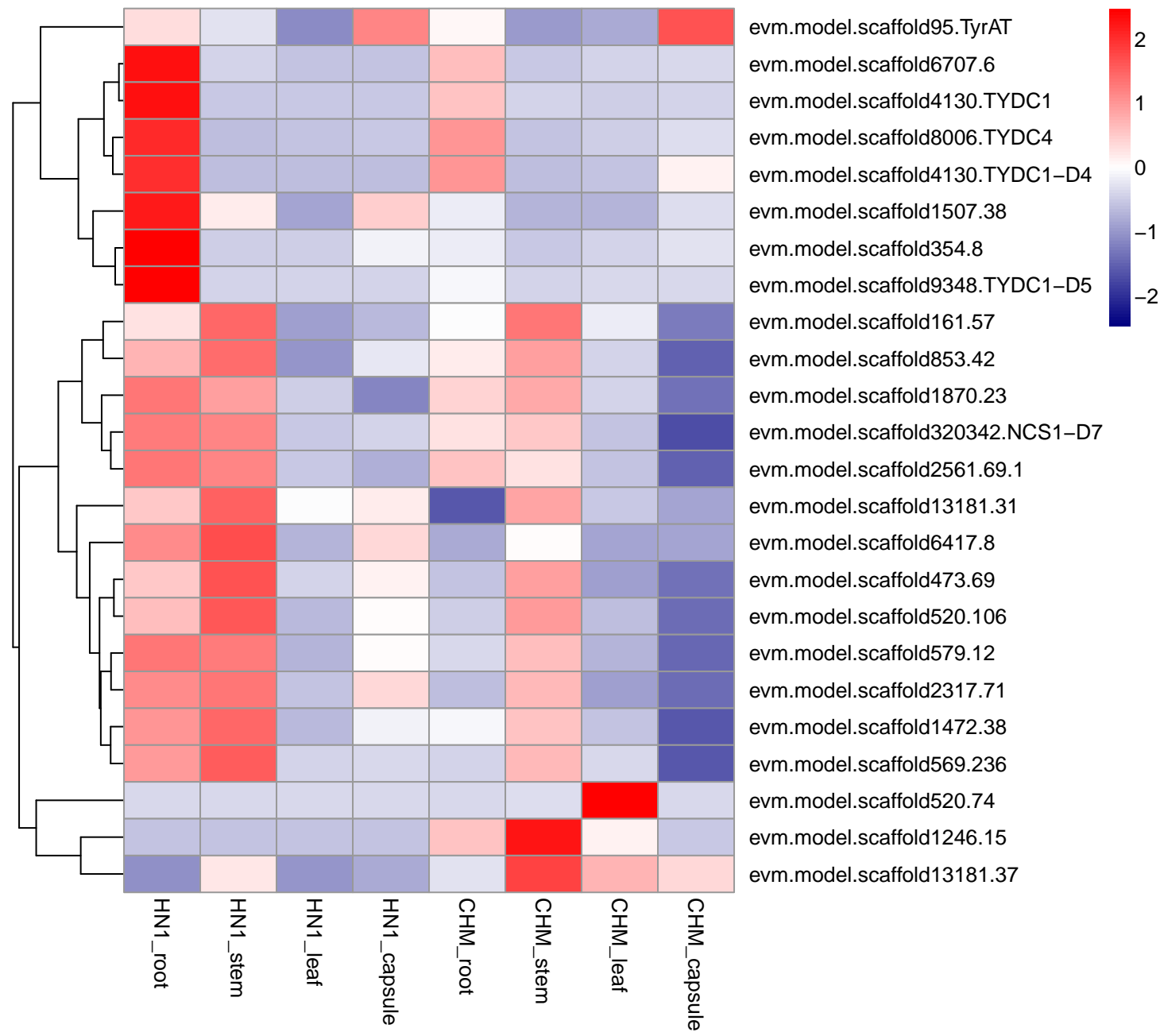

Supplement: Supplementary file 17 — Figure S15 [file 41438_2020_435_MOESM17_ESM.pdf]

Cluster analysis of differentially expressed genes

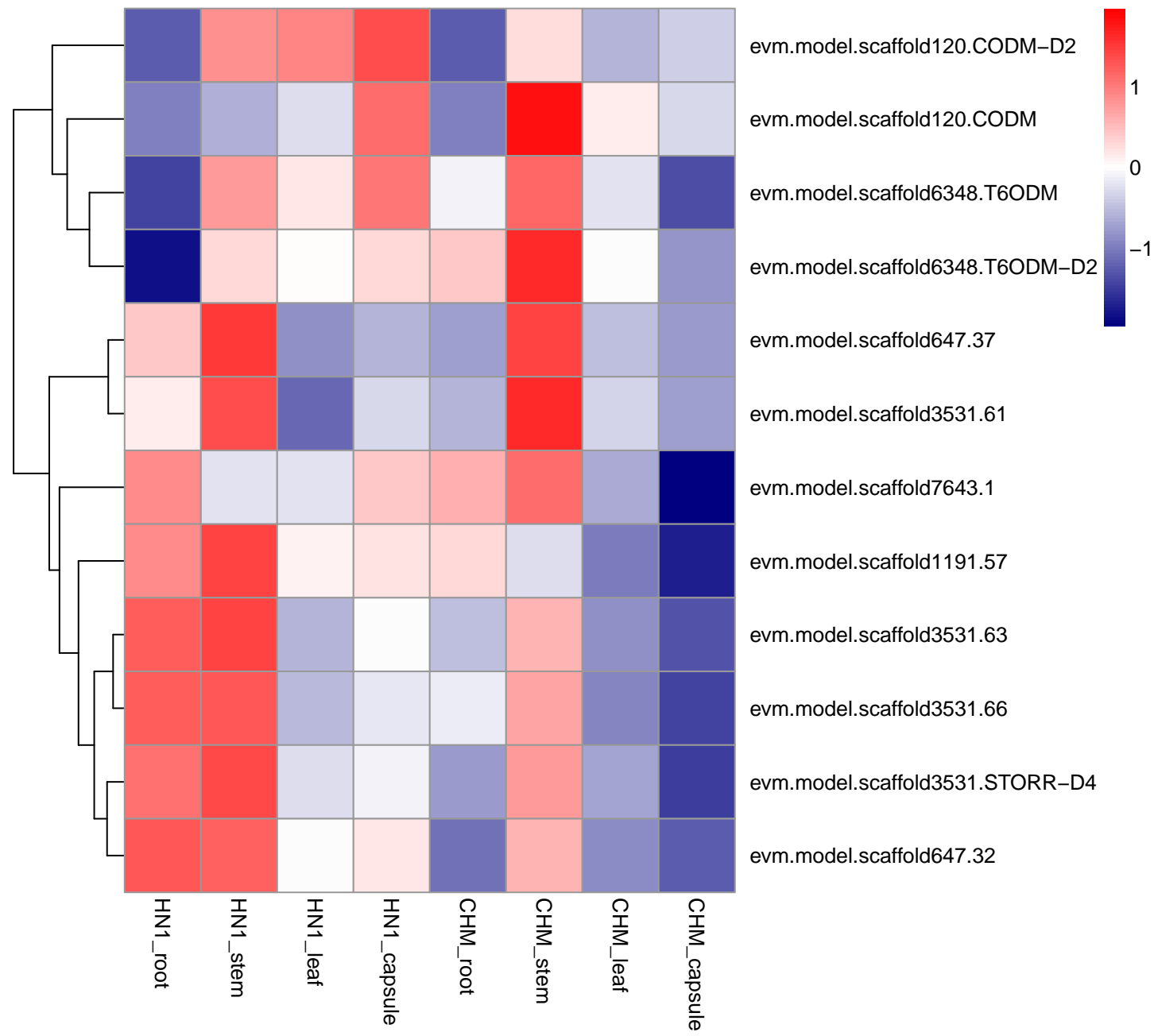

Supplement: Supplementary file 18 — Figure S16 [file 41438_2020_435_MOESM18_ESM.pdf]

Cluster analysis of differentially expressed genes

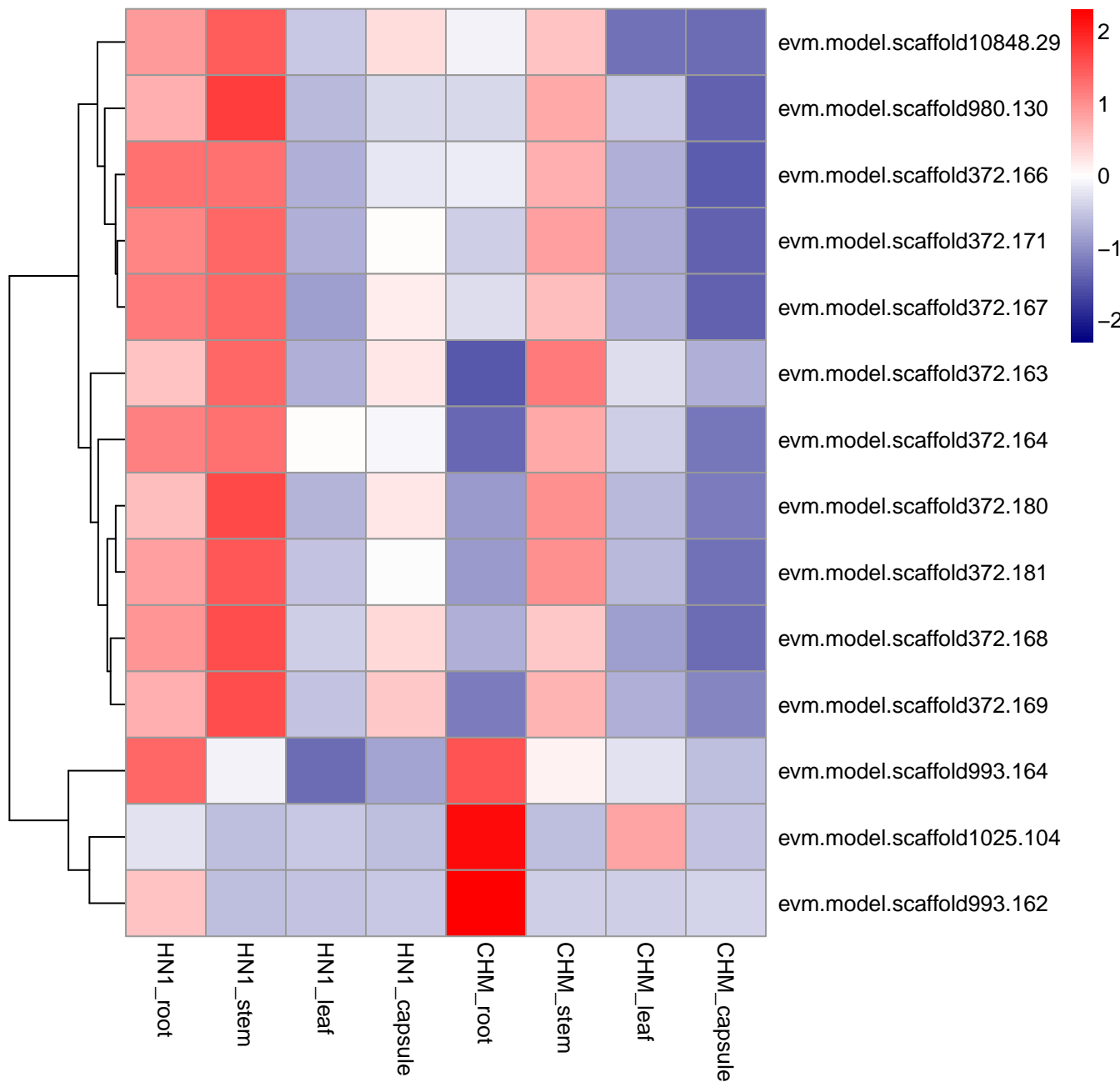

Supplement: Supplementary file 19 — Figure S17 [file 41438_2020_435_MOESM19_ESM.pdf]

Cluster analysis of differentially expressed genes

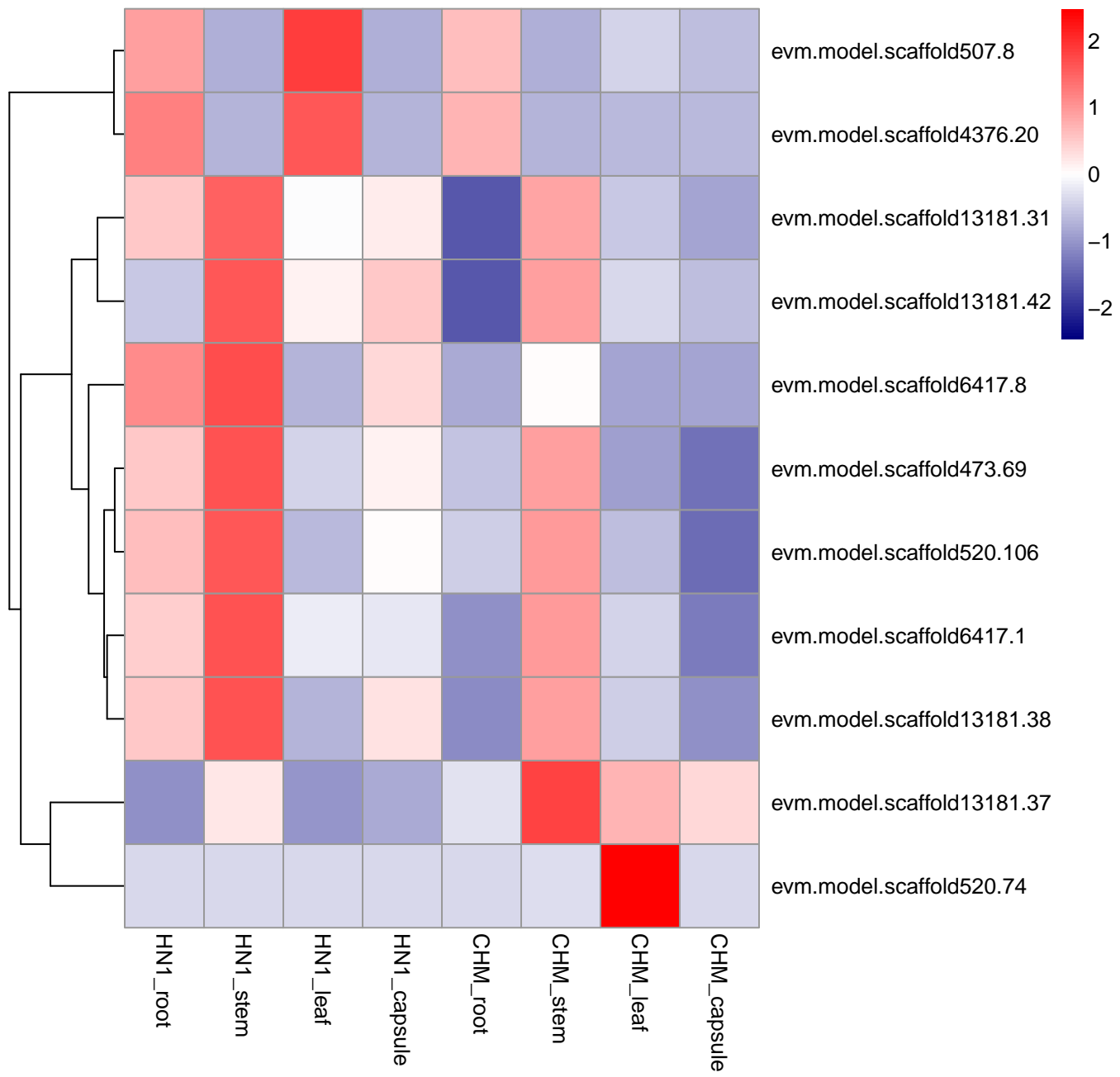

Supplement: Supplementary file 20 — Figure S18 [file 41438_2020_435_MOESM20_ESM.pdf]

Cluster analysis of differentially expressed genes

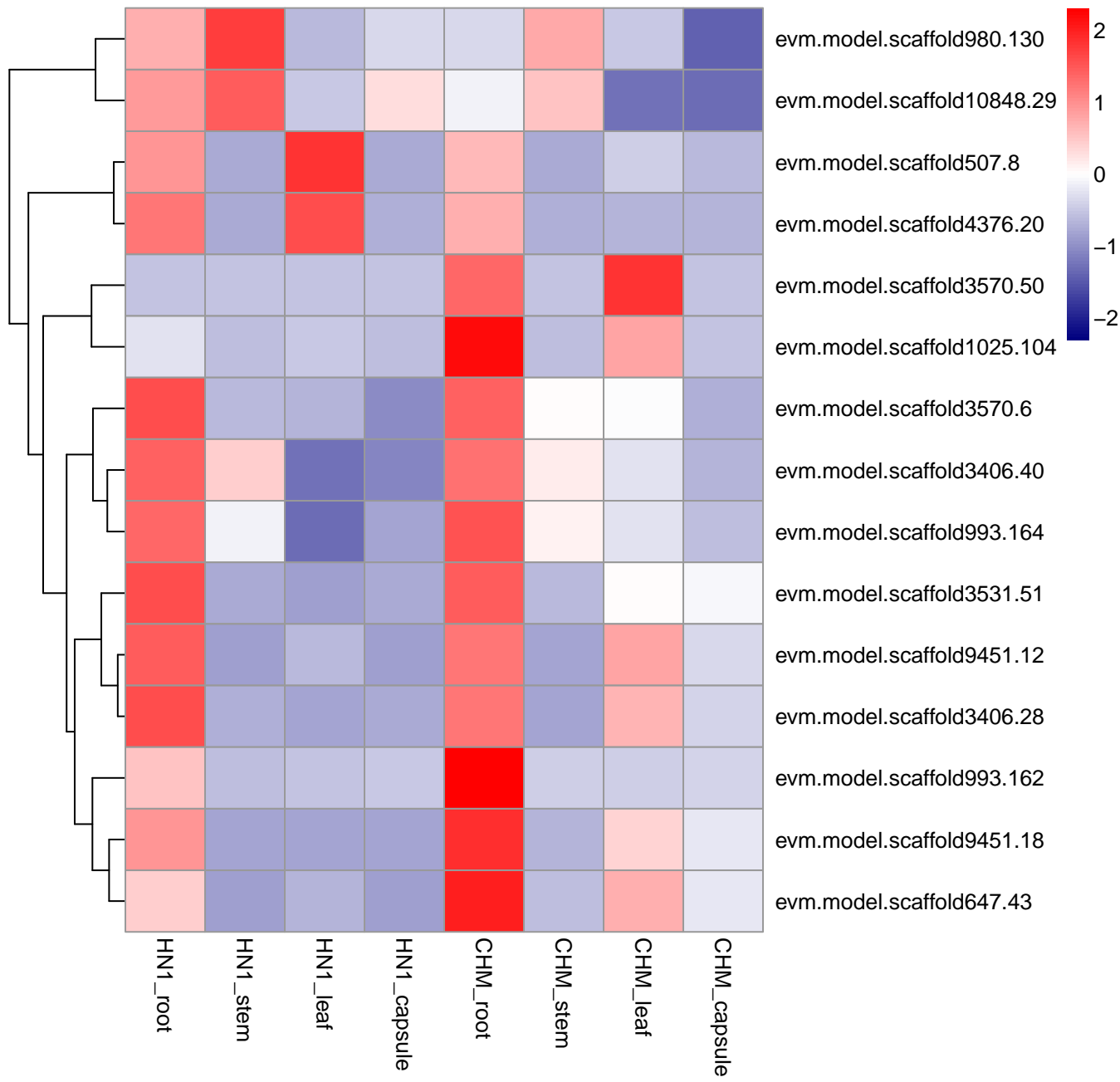

Supplement: Supplementary file 21 — Figure S19 [file 41438_2020_435_MOESM21_ESM.pdf]

Chromosome

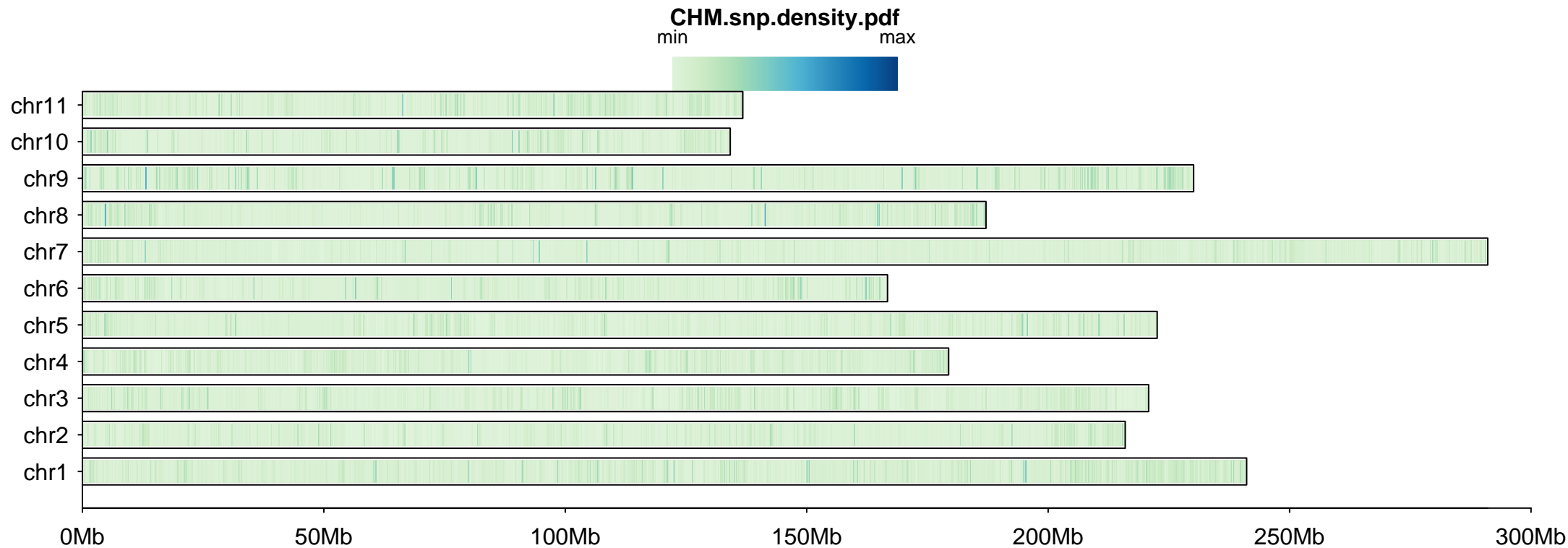

Supplement: Supplementary file 22 — Figure S20 [file 41438_2020_435_MOESM22_ESM.pdf]
