# Supplementary material for: Genome and transcriptome of Papaver somniferum Chinese landrace CHM indicates that massive genome expansion contributes to high benzylisoquinoline alkaloid biosynthesis
Source: Hortic Res. 2021 Jan 1;8:5. doi: 10.1038/s41438-020-00435-5 (PMC7775465; doi:10.1038/s41438-020-00435-5)
Supplement: Supplementary file 7 — Figure S5 [file 41438_2020_435_MOESM7_ESM.pdf]

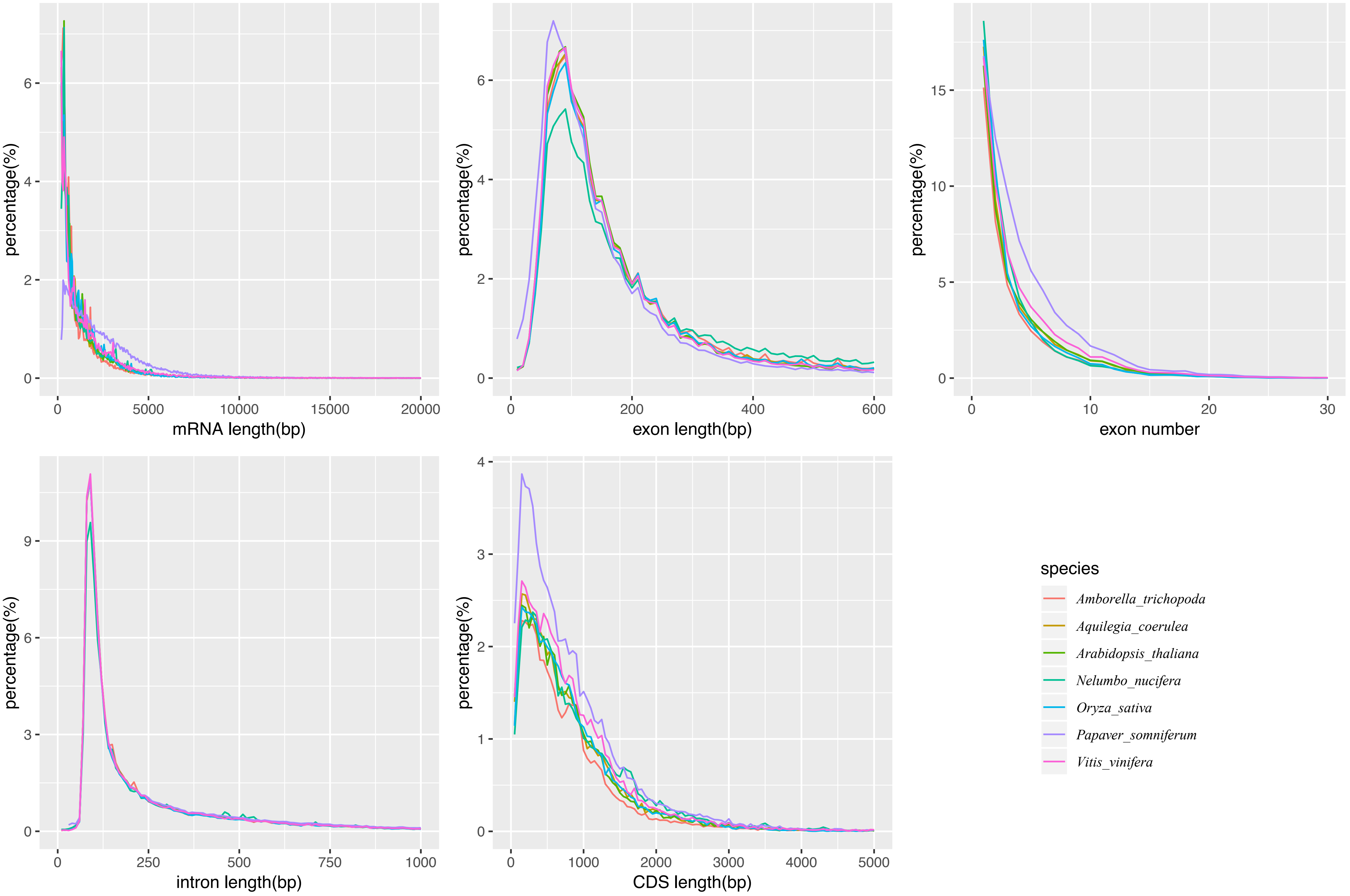

percentage(%)
mRNA length(bp)
exon length(bp)
exon number
intron length(bp)
CDS length(bp)
species
Amborella_trichopoda
Aquilegia_coerulea
Arabidopsis_thaliana
Nelumbo_nucifera
Oryza_sativa
Papaver_somniferum
Vitis_vinifera
