# Supplementary material for: Genome and transcriptome of Papaver somniferum Chinese landrace CHM indicates that massive genome expansion contributes to high benzylisoquinoline alkaloid biosynthesis
Source: Hortic Res. 2021 Jan 1;8:5. doi: 10.1038/s41438-020-00435-5 (PMC7775465; doi:10.1038/s41438-020-00435-5)
Supplement: Supplementary file 23 — Table S1 [file 41438_2020_435_MOESM23_ESM.pdf]

Table S1. Summary of sequencing data

Insert size(bp)	Read length (bp)	Raw data (Gb)	Coverage (×)
**250**	PE150	154	45.29
**350**	PE150	158	46.53
**450**	PE150	150	44.17
**2000**	PE150	120	32.95
**5000**	PE150	106	31.32
**10000**	PE150	204	60.02
**20000**	PE150	64	18.82
Total	-	956.96	279.10
