# Supplementary material for: Genome and transcriptome of Papaver somniferum Chinese landrace CHM indicates that massive genome expansion contributes to high benzylisoquinoline alkaloid biosynthesis
Source: Hortic Res. 2021 Jan 1;8:5. doi: 10.1038/s41438-020-00435-5 (PMC7775465; doi:10.1038/s41438-020-00435-5)
Supplement: Supplementary file 24 — Table S2 [file 41438_2020_435_MOESM24_ESM.pdf]

Table S2. Estimation of genome size of *P. somniferum* using 17 *K*-mer analysis.

Sample	K-mer number	K-mer Depth	Genome Size （Mbp）	Revised Genome Size (Mbp)	Heterozygous Ratio(%)	Repeat (%)
**PSO**	109,448,029,560	32	3,420.25	3,374.77	0.72	78.78
