# Supplementary material for: Genome and transcriptome of Papaver somniferum Chinese landrace CHM indicates that massive genome expansion contributes to high benzylisoquinoline alkaloid biosynthesis
Source: Hortic Res. 2021 Jan 1;8:5. doi: 10.1038/s41438-020-00435-5 (PMC7775465; doi:10.1038/s41438-020-00435-5)
Supplement: Supplementary file 25 — Table S3 [file 41438_2020_435_MOESM25_ESM.pdf]

Table S3. Summary of Hi-C sequencing data.

Type	Read length (bp)	Raw data (Gb)	Clean data (Gb)	Coverage (×)
**Hi-C**	PE150	413.34	413.30	121
