# Supplementary material for: Genome and transcriptome of Papaver somniferum Chinese landrace CHM indicates that massive genome expansion contributes to high benzylisoquinoline alkaloid biosynthesis
Source: Hortic Res. 2021 Jan 1;8:5. doi: 10.1038/s41438-020-00435-5 (PMC7775465; doi:10.1038/s41438-020-00435-5)
Supplement: Supplementary file 26 — Table S4 [file 41438_2020_435_MOESM26_ESM.pdf]

Table S4. Statistic of *P. somniferum* genome assembly.

Sample ID	Length		Number	
	Contig(bp)	Scaffold(bp)	Contig	Scaffold
**Total**	2540151652	2622282926	473482	354855
**Max**	626332	31363288	-	-
**Number ≥100**	-	-	472010	354855
**Number ≥2000**	-	-	52663	8952
**N50**	86036	6863289	8869	118
**N60**	68220	5294967	12188	162
**N70**	51589	4188743	16,463	218
**N80**	35195	2398378	22,390	301
**N90**	15996	529854	32,597	501
