# Supplementary material for: Genome and transcriptome of Papaver somniferum Chinese landrace CHM indicates that massive genome expansion contributes to high benzylisoquinoline alkaloid biosynthesis
Source: Hortic Res. 2021 Jan 1;8:5. doi: 10.1038/s41438-020-00435-5 (PMC7775465; doi:10.1038/s41438-020-00435-5)
Supplement: Supplementary file 27 — Table S5 [file 41438_2020_435_MOESM27_ESM.pdf]

Table S5. Statistic of P. somniferum genome chromosome anchoring.

chromosome	Anchored scaffolds	Length(bp)
chr1	707	241,038,189
chr2	559	215,906,516
chr3	617	220,754,802
chr4	455	179,343,961
chr5	585	222,515,483
chr6	443	166,690,438
chr7	949	290,947,881
chr8	497	187,076,360
chr9	715	230,058,263
chr10	505	134,115,430
chr11	299	136,729,732
Total	6331	2,225,177,055
