# Supplementary material for: Genome and transcriptome of Papaver somniferum Chinese landrace CHM indicates that massive genome expansion contributes to high benzylisoquinoline alkaloid biosynthesis
Source: Hortic Res. 2021 Jan 1;8:5. doi: 10.1038/s41438-020-00435-5 (PMC7775465; doi:10.1038/s41438-020-00435-5)
Supplement: Supplementary file 28 — Table S6 [file 41438_2020_435_MOESM28_ESM.pdf]

Table S8. CEGMA results of P. somniferum genome.

Species	Complete		Complete + partial	
	Prots	% completeness	Prots	% completeness
CHM	234	94.35%	240	96.77%
HN1	238	95.97	241	97.18

*CEGMA defined the number of 248 ultra-conserved CEGs that occur in a wide range of eukaryotes. A protein is classified as complete if the alignment of the predicted protein to the HMM profile represents at least 70% of the original KOG domain, otherwise is classified as partial.
