# Supplementary material for: Genome and transcriptome of Papaver somniferum Chinese landrace CHM indicates that massive genome expansion contributes to high benzylisoquinoline alkaloid biosynthesis
Source: Hortic Res. 2021 Jan 1;8:5. doi: 10.1038/s41438-020-00435-5 (PMC7775465; doi:10.1038/s41438-020-00435-5)
Supplement: Supplementary file 29 — Table S7 [file 41438_2020_435_MOESM29_ESM.pdf]

Table S14. Categories of TEs predicted in *P. somniferum* genome.

Type	Repeatmasker		TE Proteins		Combined TEs	
	**Length (bp)**	**% in Genome**	**Length (bp)**	**% in Genome**	**Length (bp)**	**% in Genome**
**DNA**	101,130,253	3.86	35,538,750	1.36	126,467,416	4.83
**LINE**	70,659,534	2.70	92,100,067	3.51	143,916,994	5.49
**LTR**	1,392,885,887	53.16	262,378,928	10.01	1,425,376,465	54.40
**SINE**	405,091	0.02	0	0	405,091	0.02
**Unknown**	4,344,790	0.17	0	0	4,344,790	0.17
**Total**	1,578,602,532	60.24	388,275,695	14.82	1,690,806,366	64.53
