# Supplementary material for: Genome and transcriptome of Papaver somniferum Chinese landrace CHM indicates that massive genome expansion contributes to high benzylisoquinoline alkaloid biosynthesis
Source: Hortic Res. 2021 Jan 1;8:5. doi: 10.1038/s41438-020-00435-5 (PMC7775465; doi:10.1038/s41438-020-00435-5)
Supplement: Supplementary file 30 — Table S8 [file 41438_2020_435_MOESM30_ESM.pdf]

Table S15. The classification and statistics of LTR in PSO and Mco

	Pso	Mco
Ty3/Gypsy	25021	1632
Ty1/Copia	13732	1806
Caulimoviridae	25	0
pepsins_A1b	9	2
Retroviridae	8	6
ddi	5	0
caulimoviridae_dom2	1	0
errantiviridae	1	0
superfamily	1	1
