# Supplementary material for: Genome and transcriptome of Papaver somniferum Chinese landrace CHM indicates that massive genome expansion contributes to high benzylisoquinoline alkaloid biosynthesis
Source: Hortic Res. 2021 Jan 1;8:5. doi: 10.1038/s41438-020-00435-5 (PMC7775465; doi:10.1038/s41438-020-00435-5)
Supplement: Supplementary file 32 — Table S10 [file 41438_2020_435_MOESM32_ESM.pdf]

Table S10. General statistics of protein-coding genes of *P. somniferum* and homolog species.

Species	Numbe r	Average transcript length (bp)	Average CDS length (bp)	Average exons per gene	Average exon length (bp)	Average intron length (bp)
Papaver somniferum	63,777	2,552.34	1,037.84	4.47	231.97	435.96
Vitis vinifera	29,927	4,728.63	1,095.81	4.75	230.72	968.88
Arabidopsis thaliana	27,416	1,870.34	1,218.40	5.13	237.58	157.91
Oryza sativa	35,679	2,165.58	991.55	3.78	262.57	422.87
Nelumbo nucifera	24,613	10,542.46	1,334.94	5.50	242.68	2,045.70
Aquilegia coerulea	30,023	2,833.22	1,141.60	4.66	245.19	462.69
Amborella trichopoda	27,313	5,607.06	944.87	4.06	232.72	1,523.51
