# Supplementary material for: Genome and transcriptome of Papaver somniferum Chinese landrace CHM indicates that massive genome expansion contributes to high benzylisoquinoline alkaloid biosynthesis
Source: Hortic Res. 2021 Jan 1;8:5. doi: 10.1038/s41438-020-00435-5 (PMC7775465; doi:10.1038/s41438-020-00435-5)
Supplement: Supplementary file 34 — Table S12 [file 41438_2020_435_MOESM34_ESM.pdf]

Table S12. General statistics of non-coding RNA of the genome.

Type		Number	Average length (bp)	Total length (bp)	% of genome
miRNA		1,404	109.52	153,771	0.0059
tRNA		5,226	73.72	385,279	0.0147
rRNA	rRNA	1,129	202.86	229,033	0.0087
	18S	319	444.11	141,671	0.0054
	28S	153	136.41	20,870	0.0008
	5.8S	56	124.73	6,985	0.0003
	5S	601	99.01	59,507	0.0023
snRNA	snRNA	2,076	114.46	237,609	0.0091
	CD-box	1,192	96.92	115,532	0.0044
	HACA-box	218	130.73	28,499	0.0011
	splicing	666	140.51	93,578	0.0036
