# Supplementary material for: Genome and transcriptome of Papaver somniferum Chinese landrace CHM indicates that massive genome expansion contributes to high benzylisoquinoline alkaloid biosynthesis
Source: Hortic Res. 2021 Jan 1;8:5. doi: 10.1038/s41438-020-00435-5 (PMC7775465; doi:10.1038/s41438-020-00435-5)
Supplement: Supplementary file 35 — Table S13 [file 41438_2020_435_MOESM35_ESM.pdf]

Table S13. The prediction of repeat elements in Papaver somniferum genome. The TRF was used to discover tandem repeats.

Type	Repeat Size (bp)	Percent (%)
TRF	123,816,918	4.73
RepeatMasker	1,578,602,532	60.24
RepeatProteinMask	388,275,695	14.82
Total	1,723,836,701	65.79

The RepeatMasker and RepeatProteinMask were used to identify TEs. The Total were integrated of all repeat elements without redundancy.
