# Supplementary material for: Genome and transcriptome of Papaver somniferum Chinese landrace CHM indicates that massive genome expansion contributes to high benzylisoquinoline alkaloid biosynthesis
Source: Hortic Res. 2021 Jan 1;8:5. doi: 10.1038/s41438-020-00435-5 (PMC7775465; doi:10.1038/s41438-020-00435-5)
Supplement: Supplementary file 37 — Table S15 [file 41438_2020_435_MOESM37_ESM.pdf]

Table S15. The KEGG enrichment result of uniq gene families in CHM

MapID	MapTitle	Pvalue	AdjustedPv
map00190	Oxidative phosphorylation	3.11E-46	8.99E-44
map00195	Photosynthesis	4.68E-39	6.77E-37
map00260	Glycine, serine and threonine metabolism	3.65E-17	3.52E-15
map03010	Ribosome	2.21E-15	1.60E-13
map00670	One carbon pool by folate	3.58E-13	2.07E-11
map00630	Glyoxylate and dicarboxylate metabolism	6.15E-11	2.96E-09
map00460	Cyanoamino acid metabolism	6.59E-08	2.12E-06
map02010	ABC transporters	2.45E-07	6.43E-06
map01200	Carbon metabolism	8.04E-05	0.001348845
map03050	Proteasome	0.0002794	0.003927993
map00520	Amino sugar and nucleotide sugar metabolism	0.00028543	0.003927993
map00350	Tyrosine metabolism	0.00060317	0.007263141
map04070	Phosphatidylinositol signaling system	0.00306934	0.030587595
map00950	Isoquinoline alkaloid biosynthesis	0.0038006	0.035431439
map00960	Tropane, piperidine and pyridine alkaloid biosynthesis	0.00639308	0.056167946
map04145	Phagosome	0.01008192	0.078747947
map03440	Homologous recombination	0.01189267	0.088127735
map00240	Pyrimidine metabolism	0.02514855	0.151415238
map00410	beta-Alanine metabolism	0.06156489	0.300187735
map00010	Glycolysis / Gluconeogenesis	0.0706683	0.329405462
map00220	Arginine biosynthesis	0.08135948	0.361736752
map01230	Biosynthesis of amino acids	0.09138969	0.371524546
map03450	Non-homologous end-joining	0.09238499	0.371524546
map03020	RNA polymerase	0.12984395	0.441469418
map00603	Glycosphingolipid biosynthesis - globo and isoglobo ser	0.13800058	0.443135181
map00100	Steroid biosynthesis	0.14635391	0.458140028
map04120	Ubiquitin mediated proteolysis	0.16094499	0.460525767
map00562	Inositol phosphate metabolism	0.20841507	0.506150875
map00780	Biotin metabolism	0.23185883	0.536162653
map00944	Flavone and flavonol biosynthesis	0.2337595	0.536162653
map00740	Riboflavin metabolism	0.23747295	0.536169405
map00785	Lipoic acid metabolism	0.2798458	0.569545328
map00640	Propanoate metabolism	0.29317007	0.584043661
map00196	Photosynthesis - antenna proteins	0.29686142	0.584043661
map00360	Phenylalanine metabolism	0.32604042	0.6116768
map03022	Basal transcription factors	0.3518722	0.641366764
map00072	Synthesis and degradation of ketone bodies	0.38626268	0.664463777
map00480	Glutathione metabolism	0.43707639	0.717699302
map00020	Citrate cycle (TCA cycle)	0.53249231	0.780451164
map00250	Alanine, aspartate and glutamate metabolism	0.59524216	0.823086043
map00910	Nitrogen metabolism	0.6067507	0.831382802
map04712	Circadian rhythm - plant	0.60699575	0.831382802
map00261	Monobactam biosynthesis	0.65864528	0.883768331
map00966	Glucosinolate biosynthesis	0.66927169	0.887245497
map00908	Zeatin biosynthesis	0.69832134	0.905573228
map00604	Glycosphingolipid biosynthesis - ganglio series	0.77789647	0.947926392
map00620	Pyruvate metabolism	0.78124373	0.947926392
map04075	Plant hormone signal transduction	0.82942901	0.978387694

number
80
49
44
105
27
33
33
37
69
22
40
23
23
18
13
24
19
33
15
30
11
50
3
12
4
9
38
16
5
1
5
2
6
6
12
11
1
15
9
9
5
13
2
2
2
4
14
45
