# Supplementary material for: Genome and transcriptome of Papaver somniferum Chinese landrace CHM indicates that massive genome expansion contributes to high benzylisoquinoline alkaloid biosynthesis
Source: Hortic Res. 2021 Jan 1;8:5. doi: 10.1038/s41438-020-00435-5 (PMC7775465; doi:10.1038/s41438-020-00435-5)
Supplement: Supplementary file 38 — Table S16 [file 41438_2020_435_MOESM38_ESM.pdf]

Table S16. The KEGG enrichment result of uniq gene families in HN1

MapID	MapTitle	AdjustedP\	number
map00966	Glucosinolate biosynthesis	0	319
map00460	Cyanoamino acid metabolism	9.89E-293	370
map01210	2-Oxocarboxylic acid metabolism	9.95E-254	345
map00591	Linoleic acid metabolism	5.62E-253	178
map00053	Ascorbate and aldarate metabolism	4.94E-149	177
map00710	Carbon fixation in photosynthetic organisms	1.44E-134	219
map00040	Pentose and glucuronate interconversions	3.14E-113	233
map00052	Galactose metabolism	2.45E-107	184
map00030	Pentose phosphate pathway	2.43E-92	192
map00520	Amino sugar and nucleotide sugar metabolism	3.14E-80	209
map00051	Fructose and mannose metabolism	1.38E-65	142
map00190	Oxidative phosphorylation	8.90E-61	132
map01200	Carbon metabolism	1.72E-60	290
map00010	Glycolysis / Gluconeogenesis	2.34E-49	154
map03040	Spliceosome	5.51E-42	222
map00630	Glyoxylate and dicarboxylate metabolism	4.30E-21	77
map01230	Biosynthesis of amino acids	2.38E-18	213
map04070	Phosphatidylinositol signaling system	6.73E-18	104
map02010	ABC transporters	8.56E-15	96
map03410	Base excision repair	1.47E-07	43
map00480	Glutathione metabolism	4.70E-06	77
map01040	Biosynthesis of unsaturated fatty acids	2.38E-05	43
map00061	Fatty acid biosynthesis	0.000811	44
map00130	Ubiquinone and other terpenoid-quinone biosynthesis	0.000902	25
map00073	Cutin, suberine and wax biosynthesis	0.057563	42
map04146	Peroxisome	0.092321	44
map01212	Fatty acid metabolism	0.113483	46
map00450	Selenocompound metabolism	0.119866	11
map00300	Lysine biosynthesis	0.184362	17
map03430	Mismatch repair	0.329052	22
map03015	mRNA surveillance pathway	0.337079	61
map00020	Citrate cycle (TCA cycle)	0.400935	17
map00563	Glycosylphosphatidylinositol (GPI)-anchor biosynthesis	0.443802	12
map00310	Lysine degradation	0.587297	27
map03020	RNA polymerase	0.639169	20
map00500	Starch and sucrose metabolism	0.67403	125
