# Supplementary material for: Genome and transcriptome of Papaver somniferum Chinese landrace CHM indicates that massive genome expansion contributes to high benzylisoquinoline alkaloid biosynthesis
Source: Hortic Res. 2021 Jan 1;8:5. doi: 10.1038/s41438-020-00435-5 (PMC7775465; doi:10.1038/s41438-020-00435-5)
Supplement: Supplementary file 39 — Table S17 [file 41438_2020_435_MOESM39_ESM.pdf]

Table S17.The KEGG enrichment result of common gene families in CHM and HN1

MapID	MapTitle	AdjustedP\	number
map02010	ABC transporters	1.76E-74	191
map00073	Cutin, suberine and wax biosynthesis	1.53E-42	129
map00460	Cyanoamino acid metabolism	2.47E-39	135
map00945	Stilbenoid, diarylheptanoid and gingerol biosynthesis	3.92E-28	119
map04626	Plant-pathogen interaction	8.32E-24	282
map04122	Sulfur relay system	3.62E-21	50
map00591	Linoleic acid metabolism	3.56E-20	45
map00950	Isoquinoline alkaloid biosynthesis	6.57E-19	82
map00903	Limonene and pinene degradation	4.80E-17	67
map00730	Thiamine metabolism	3.20E-15	50
map00940	Phenylpropanoid biosynthesis	5.81E-14	175
map00350	Tyrosine metabolism	2.54E-12	85
map04144	Endocytosis	1.13E-10	244
map00052	Galactose metabolism	1.55E-10	91
map00592	alpha-Linolenic acid metabolism	2.08E-10	70
map00196	Photosynthesis - antenna proteins	3.27E-09	37
map00909	Sesquiterpenoid and triterpenoid biosynthesis	7.77E-08	21
map00941	Flavonoid biosynthesis	5.78E-07	75
map00500	Starch and sucrose metabolism	5.54E-06	227
map00670	One carbon pool by folate	0.000106	35
map00402	Benzoxazinoid biosynthesis	0.000507	19
map00943	Isoflavonoid biosynthesis	0.000684	22
map00062	Fatty acid elongation	0.000752	47
map00430	Taurine and hypotaurine metabolism	0.004563	11
map00531	Glycosaminoglycan degradation	0.009395	31
map00966	Glucosinolate biosynthesis	0.019355	12
map00604	Glycosphingolipid biosynthesis - ganglio series	0.031667	21
map00380	Tryptophan metabolism	0.049824	35
map00910	Nitrogen metabolism	0.063542	23
map00053	Ascorbate and aldarate metabolism	0.071141	39
map00514	Other types of O-glycan biosynthesis	0.072196	21
map00310	Lysine degradation	0.098708	35
map00410	beta-Alanine metabolism	0.126412	43
map00270	Cysteine and methionine metabolism	0.126729	67
map00232	Caffeine metabolism	0.191159	8
map00340	Histidine metabolism	0.193456	19
map04933	AGE-RAGE signaling pathway in diabetic complications	0.430253	20
map00590	Arachidonic acid metabolism	0.430253	23
map00330	Arginine and proline metabolism	0.521619	36
map00942	Anthocyanin biosynthesis	0.55163	6
map00565	Ether lipid metabolism	0.712191	19
map00511	Other glycan degradation	0.765004	36
map00920	Sulfur metabolism	0.859004	20
map00650	Butanoate metabolism	0.907644	11
