# Supplementary material for: Genome and transcriptome of Papaver somniferum Chinese landrace CHM indicates that massive genome expansion contributes to high benzylisoquinoline alkaloid biosynthesis
Source: Hortic Res. 2021 Jan 1;8:5. doi: 10.1038/s41438-020-00435-5 (PMC7775465; doi:10.1038/s41438-020-00435-5)
Supplement: Supplementary file 40 — Table S18 [file 41438_2020_435_MOESM40_ESM.pdf]

Table S18.The GO enrichment result of common gene families in CHM and HN1

GO_ID	GO_Term	GO_Class	AdjustedP\	number
GO:0032559	adenyl ribonucleotide binding	MF	3.63E-185	1326
GO:0043168	anion binding	MF	3.19E-182	1593
GO:0032549	ribonucleoside binding	MF	4.45E-168	1418
GO:0032553	ribonucleotide binding	MF	2.54E-167	1423
GO:0032550	purine ribonucleoside binding	MF	1.65E-166	1409
GO:0032555	purine ribonucleotide binding	MF	1.65E-166	1409
GO:0097367	carbohydrate derivative binding	MF	4.26E-165	1432
GO:0000166	nucleotide binding	MF	8.41E-165	1535
GO:0004713	protein tyrosine kinase activity	MF	1.26E-161	635
GO:0036094	small molecule binding	MF	1.69E-161	1557
GO:0003824	catalytic activity	MF	1.75E-152	3427
GO:0005524	ATP binding	MF	4.44E-136	1182
GO:0043167	ion binding	MF	2.10E-133	2264
GO:0035639	purine ribonucleoside triphosphate binding	MF	8.83E-121	1265
GO:0004672	protein kinase activity	MF	2.30E-120	669
GO:0006468	protein phosphorylation	BP	1.53E-114	656
GO:0005215	transporter activity	MF	1.41E-104	612
GO:0016310	phosphorylation	BP	6.00E-99	658
GO:0022857	transmembrane transporter activity	MF	2.84E-98	497
GO:0016773	phosphotransferase activity, alcohol group as acceptor	MF	1.42E-97	704
GO:1901363	heterocyclic compound binding	MF	1.08E-94	2237
GO:0097159	organic cyclic compound binding	MF	1.18E-94	2237
GO:0016740	transferase activity	MF	2.52E-88	1372
GO:0055085	transmembrane transport	BP	5.50E-87	506
GO:0043531	ADP binding	MF	1.36E-83	144
GO:0016301	kinase activity	MF	6.22E-83	689
GO:0022804	active transmembrane transporter activity	MF	4.04E-78	273
GO:0005506	iron ion binding	MF	2.39E-77	365
GO:0006464	cellular protein modification process	BP	2.15E-74	688
GO:0044699	single-organism process	BP	1.52E-71	2020
GO:0016020	membrane	CC	5.43E-70	1060
GO:0044765	single-organism transport	BP	4.59E-69	655
GO:0020037	heme binding	MF	6.05E-69	376
GO:0016705	oxidoreductase activity, acting on paired donors, with i	MF	8.71E-69	394
GO:0043412	macromolecule modification	BP	3.84E-68	703
GO:0043492	ATPase activity, coupled to movement of substances	MF	3.28E-64	168
GO:0042626	ATPase activity, coupled to transmembrane movement	MF	6.45E-61	163
GO:0016772	transferase activity, transferring phosphorus-containing	MF	1.50E-59	738
GO:0043086	negative regulation of catalytic activity	BP	8.40E-58	80
GO:0016021	integral component of membrane	CC	3.42E-57	507
GO:0051274	beta-glucan biosynthetic process	BP	3.07E-51	75
GO:0016887	ATPase activity	MF	4.28E-51	309
GO:0022891	substrate-specific transmembrane transporter activity	MF	7.12E-50	319
GO:0055114	oxidation-reduction process	BP	8.65E-50	747
GO:0035251	UDP-glucosyltransferase activity	MF	1.13E-49	80
GO:0042802	identical protein binding	MF	2.35E-48	80
GO:0017111	nucleoside-triphosphatase activity	MF	6.76E-48	507
GO:0022892	substrate-specific transporter activity	MF	1.29E-47	332
GO:0006810	transport	BP	2.68E-45	784
GO:0016760	cellulose synthase (UDP-forming) activity	MF	3.26E-42	52
GO:0030246	carbohydrate binding	MF	1.40E-41	125
GO:0016491	oxidoreductase activity	MF	2.57E-41	814
GO:0006796	phosphate-containing compound metabolic process	BP	1.15E-40	691
GO:0008762	UDP-N-acetylmuramate dehydrogenase activity	MF	5.59E-38	64
GO:0030247	polysaccharide binding	MF	1.41E-34	66
GO:0042623	ATPase activity, coupled	MF	3.89E-34	226

GO:0015075	ion transmembrane transporter activity	MF	1.02E-33	253
GO:0015299	solute:proton antiporter activity	MF	1.61E-33	61
GO:0044425	membrane part	CC	1.69E-31	537
GO:0015297	antiporter activity	MF	4.61E-31	110
GO:0071805	potassium ion transmembrane transport	BP	4.94E-31	42
GO:0030244	cellulose biosynthetic process	BP	1.03E-30	52
GO:0016758	transferase activity, transferring hexosyl groups	MF	2.44E-30	220
GO:0009055	electron carrier activity	MF	3.43E-29	328
GO:0008194	UDP-glycosyltransferase activity	MF	8.50E-26	90
GO:0004097	catechol oxidase activity	MF	8.57E-24	34
GO:0016051	carbohydrate biosynthetic process	BP	1.16E-23	81
GO:0006820	anion transport	BP	1.72E-22	89
GO:0044710	single-organism metabolic process	BP	6.57E-22	1211
GO:0006952	defense response	BP	1.30E-21	148
GO:0015077	monovalent inorganic cation transmembrane transport	MF	1.68E-21	125
GO:0004970	ionotropic glutamate receptor activity	MF	1.79E-21	29
GO:0005234	extracellular-glutamate-gated ion channel activity	MF	1.79E-21	29
GO:0016747	transferase activity, transferring acyl groups other than	MF	7.02E-21	198
GO:0016787	hydrolase activity	MF	1.12E-20	1090
GO:0016757	transferase activity, transferring glycosyl groups	MF	2.34E-20	232
GO:0008324	cation transmembrane transporter activity	MF	4.19E-20	188
GO:0016746	transferase activity, transferring acyl groups	MF	4.86E-20	223
GO:0015079	potassium ion transmembrane transporter activity	MF	1.32E-19	45
GO:0003843	1,3-beta-D-glucan synthase activity	MF	2.08E-19	28
GO:0038023	signaling receptor activity	MF	2.16E-19	42
GO:0015103	inorganic anion transmembrane transporter activity	MF	6.35E-19	38
GO:0006811	ion transport	BP	1.01E-18	250
GO:0004252	serine-type endopeptidase activity	MF	1.24E-18	96
GO:0006813	potassium ion transport	BP	2.04E-18	45
GO:0016701	oxidoreductase activity, acting on single donors with in	MF	2.59E-17	46
GO:0008272	sulfate transport	BP	3.10E-17	26
GO:0015116	sulfate transmembrane transporter activity	MF	3.10E-17	26
GO:0016831	carboxy-lyase activity	MF	9.19E-17	49
GO:0016165	linoleate 13S-lipoxygenase activity	MF	1.23E-16	24
GO:0016702	oxidoreductase activity, acting on single donors with in	MF	4.16E-16	34
GO:0050660	flavin adenine dinucleotide binding	MF	4.64E-16	81
GO:0000148	1,3-beta-D-glucan synthase complex	CC	6.86E-16	23
GO:0006075	(1->3)-beta-D-glucan biosynthetic process	BP	6.86E-16	23
GO:0022890	inorganic cation transmembrane transporter activity	MF	1.79E-15	144
GO:0004012	phospholipid-translocating ATPase activity	MF	2.20E-15	21
GO:0015914	phospholipid transport	BP	2.20E-15	21
GO:0015698	inorganic anion transport	BP	4.05E-15	38
GO:0004871	signal transducer activity	MF	1.49E-14	75
GO:0004372	glycine hydroxymethyltransferase activity	MF	1.85E-14	33
GO:0080019	fatty-acyl-CoA reductase (alcohol-forming) activity	MF	1.05E-13	24
GO:0030170	pyridoxal phosphate binding	MF	1.38E-13	68
GO:0004144	diacylglycerol O-acyltransferase activity	MF	5.85E-13	25
GO:0018298	protein-chromophore linkage	BP	2.42E-12	17
GO:0005488	binding	MF	2.57E-12	3206
22,evm.model.scaffold6856.4,evm.model.scaffold769.41,evm.model.scaffold7923.42,evm.model.scaffolc				
affold2491.21,evm.model.scaffold6597.1,evm.model.scaffold7227.11,evm.model.scaffold1038.38,evm.m				
GO:0050664	oxidoreductase activity, acting on NAD(P)H, oxygen as	MF	5.88E-12	15
GO:0044763	single-organism cellular process	BP	1.35E-11	1063
GO:0003885	D-arabinono-1,4-lactone oxidase activity	MF	1.86E-11	17
GO:0008152	metabolic process	BP	1.97E-11	2861
ld169.79,evm.model.scaffold1991.52,evm.model.scaffold2095.91,evm.model.scaffold2244.83,evm.mode				
del.scaffold567.50,evm.model.scaffold721.30,evm.model.scaffold721.32,evm.model.scaffold7554.14,evn				
GO:0006812	cation transport	BP	2.18E-11	188

GO:0048037	cofactor binding	MF	2.95E-11	202
GO:0000786	nucleosome	CC	3.49E-11	86
GO:0050896	response to stimulus	BP	5.12E-11	405
GO:0048038	quinone binding	MF	2.31E-10	26
GO:0009725	response to hormone	BP	2.95E-10	20
GO:0009584	detection of visible light	BP	4.21E-10	17
GO:0004497	monooxygenase activity	MF	4.59E-10	47
GO:0004175	endopeptidase activity	MF	5.56E-10	186
GO:0009765	photosynthesis, light harvesting	BP	7.99E-10	37
GO:0005509	calcium ion binding	MF	8.25E-10	115
GO:0006461	protein complex assembly	BP	8.31E-10	120
GO:0015743	malate transport	BP	2.59E-09	21
GO:0044262	cellular carbohydrate metabolic process	BP	3.08E-09	82
GO:0022836	gated channel activity	MF	4.69E-09	32
GO:0019829	cation-transporting ATPase activity	MF	5.42E-09	59
GO:0019538	protein metabolic process	BP	5.71E-09	997
GO:0006563	L-serine metabolic process	BP	6.18E-09	33
GO:0043682	copper-transporting ATPase activity	MF	1.20E-08	14
GO:0072330	monocarboxylic acid biosynthetic process	BP	2.04E-08	70
GO:0006334	nucleosome assembly	BP	2.12E-08	87
GO:0034622	cellular macromolecular complex assembly	BP	2.72E-08	113
GO:0006544	glycine metabolic process	BP	2.80E-08	33
GO:0005886	plasma membrane	CC	3.79E-08	25
GO:0006633	fatty acid biosynthetic process	BP	4.91E-08	60
GO:0004435	phosphatidylinositol phospholipase C activity	MF	6.42E-08	11
GO:0004351	glutamate decarboxylase activity	MF	6.42E-08	11
GO:0051213	dioxygenase activity	MF	9.62E-08	89
GO:0008236	serine-type peptidase activity	MF	1.10E-07	99
GO:0006325	chromatin organization	BP	1.52E-07	102
GO:0004478	methionine adenosyltransferase activity	MF	2.30E-07	9
GO:0006556	S-adenosylmethionine biosynthetic process	BP	2.30E-07	9
GO:0004221	ubiquitin thiolesterase activity	MF	2.61E-07	33
GO:0005884	actin filament	CC	3.37E-07	11
GO:0004930	G-protein coupled receptor activity	MF	3.44E-07	10
GO:0008509	anion transmembrane transporter activity	MF	3.53E-07	47
GO:0016901	oxidoreductase activity, acting on the CH-OH group of	MF	3.66E-07	14
GO:0046872	metal ion binding	MF	5.28E-07	810
GO:0046914	transition metal ion binding	MF	5.78E-07	604
GO:0008610	lipid biosynthetic process	BP	8.28E-07	113
GO:0005337	nucleoside transmembrane transporter activity	MF	9.55E-07	16
GO:0015078	hydrogen ion transmembrane transporter activity	MF	1.47E-06	68
GO:0042221	response to chemical	BP	1.73E-06	60
GO:0004190	aspartic-type endopeptidase activity	MF	1.85E-06	73
GO:0015711	organic anion transport	BP	2.33E-06	42
GO:0046873	metal ion transmembrane transporter activity	MF	2.63E-06	64
GO:0004650	polygalacturonase activity	MF	3.66E-06	35
GO:0044427	chromosomal part	CC	4.06E-06	93
GO:0003868	4-hydroxyphenylpyruvate dioxygenase activity	MF	5.77E-06	10
GO:0005216	ion channel activity	MF	7.06E-06	33
GO:0006629	lipid metabolic process	BP	1.29E-05	213
GO:0005975	carbohydrate metabolic process	BP	1.82E-05	288
GO:0016298	lipase activity	MF	2.52E-05	32
GO:0005452	inorganic anion exchanger activity	MF	2.60E-05	9
GO:0098662	inorganic cation transmembrane transport	BP	2.60E-05	60
GO:0018580	nitronate monooxygenase activity	MF	2.77E-05	12
GO:0031519	PcG protein complex	CC	4.35E-05	6
GO:0006306	DNA methylation	BP	6.84E-05	15
GO:0051258	protein polymerization	BP	6.84E-05	26

GO:0030674	protein binding, bridging	MF	6.84E-05	9
GO:0051015	actin filament binding	MF	6.84E-05	9
GO:0004601	peroxidase activity	MF	7.21E-05	68
GO:0044085	cellular component biogenesis	BP	7.37E-05	152
GO:0000159	protein phosphatase type 2A complex	CC	8.97E-05	13
GO:0008601	protein phosphatase type 2A regulator activity	MF	8.97E-05	13
GO:0008519	ammonium transmembrane transporter activity	MF	9.55E-05	12
GO:0015696	ammonium transport	BP	9.55E-05	12
GO:0044267	cellular protein metabolic process	BP	0.000109	782
GO:0006606	protein import into nucleus	BP	0.00011	8
GO:0006825	copper ion transport	BP	0.000128	14
GO:0008131	primary amine oxidase activity	MF	0.000164	12
GO:0006855	drug transmembrane transport	BP	0.000164	40
GO:0015238	drug transmembrane transporter activity	MF	0.000164	40
GO:0034968	histone lysine methylation	BP	0.000191	15
GO:0004612	phosphoenolpyruvate carboxykinase (ATP) activity	MF	0.000231	6
GO:0010333	terpene synthase activity	MF	0.000234	13
GO:0008081	phosphoric diester hydrolase activity	MF	0.000296	18
GO:0072488	ammonium transmembrane transport	BP	0.0003	11
GO:0007165	signal transduction	BP	0.000499	118
GO:0016614	oxidoreductase activity, acting on CH-OH group of dor	MF	0.00077	85
GO:0045010	actin nucleation	BP	0.000794	11
GO:0004553	hydrolase activity, hydrolyzing O-glycosyl compounds	MF	0.00082	156
GO:0005200	structural constituent of cytoskeleton	MF	0.001131	15
GO:0004499	N,N-dimethylaniline monooxygenase activity	MF	0.001198	16
GO:0004965	G-protein coupled GABA receptor activity	MF	0.001305	4
GO:0030976	thiamine pyrophosphate binding	MF	0.001391	13
GO:0004674	protein serine/threonine kinase activity	MF	0.001503	21
GO:0000287	magnesium ion binding	MF	0.001623	49
GO:0006536	glutamate metabolic process	BP	0.001757	11
GO:0006950	response to stress	BP	0.001867	225
GO:0004664	prephenate dehydratase activity	MF	0.002643	9
GO:0009094	L-phenylalanine biosynthetic process	BP	0.002643	9
GO:0015672	monovalent inorganic cation transport	BP	0.003085	64
GO:0017038	protein import	BP	0.003212	14
GO:0004386	helicase activity	MF	0.003333	88
GO:0009405	pathogenesis	BP	0.003642	6
GO:0070011	peptidase activity, acting on L-amino acid peptides	MF	0.004094	206
GO:0016741	transferase activity, transferring one-carbon groups	MF	0.00517	134
GO:0043414	macromolecule methylation	BP	0.007319	30
GO:0008026	ATP-dependent helicase activity	MF	0.009792	58
GO:0044711	single-organism biosynthetic process	BP	0.010105	240
GO:0016616	oxidoreductase activity, acting on the CH-OH group of	MF	0.010181	70
GO:0003924	GTPase activity	MF	0.010586	72
GO:0006508	proteolysis	BP	0.011619	246
GO:0008171	O-methyltransferase activity	MF	0.01188	27
GO:0000155	phosphorelay sensor kinase activity	MF	0.015577	13
GO:0007154	cell communication	BP	0.015792	126
GO:0045017	glycerolipid biosynthetic process	BP	0.016146	23
GO:0006904	vesicle docking involved in exocytosis	BP	0.016312	12
GO:0004594	pantothenate kinase activity	MF	0.01716	6
GO:0042176	regulation of protein catabolic process	BP	0.020395	8
GO:0015937	coenzyme A biosynthetic process	BP	0.025439	6
GO:0004813	alanine-tRNA ligase activity	MF	0.030715	11
GO:0006419	alanyl-tRNA aminoacylation	BP	0.030715	11
GO:0030328	prenylcysteine catabolic process	BP	0.038444	2
GO:0006200	ATP catabolic process	BP	0.038444	2
GO:0005874	microtubule	CC	0.040453	15

GO:0018024	histone-lysine N-methyltransferase activity	MF	0.040453	15
GO:0042393	histone binding	MF	0.040453	15
GO:0004806	triglyceride lipase activity	MF	0.044814	21
GO:0008889	glycerophosphodiester phosphodiesterase activity	MF	0.046499	7
GO:0016706	oxidoreductase activity, acting on paired donors, with i	MF	0.046499	55
GO:0006979	response to oxidative stress	BP	0.049126	53
GO:0004824	lysine-tRNA ligase activity	MF	0.061265	4
GO:0006430	lysyl-tRNA aminoacylation	BP	0.061265	4
GO:0006094	gluconeogenesis	BP	0.062499	6
GO:0004747	ribokinase activity	MF	0.080017	6
GO:0006014	D-ribose metabolic process	BP	0.080017	6
GO:0051260	protein homooligomerization	BP	0.090058	7
GO:0000808	origin recognition complex	CC	0.090058	7
GO:0050662	coenzyme binding	MF	0.098015	108
GO:0044255	cellular lipid metabolic process	BP	0.099396	100
GO:0010181	FMN binding	MF	0.123816	14
GO:0006071	glycerol metabolic process	BP	0.124874	7
GO:0006887	exocytosis	BP	0.140724	18
GO:0003854	3-beta-hydroxy-delta5-steroid dehydrogenase activity	MF	0.140741	6
GO:0005507	copper ion binding	MF	0.15464	53
GO:0044430	cytoskeletal part	CC	0.197408	53
GO:0030410	nicotianamine synthase activity	MF	0.206973	10
GO:0030418	nicotianamine biosynthetic process	BP	0.206973	10
GO:0000275	mitochondrial proton-transporting ATP synthase comp	CC	0.228752	2
GO:0031047	gene silencing by RNA	BP	0.238171	9
GO:0009072	aromatic amino acid family metabolic process	BP	0.244929	19
GO:0016829	lyase activity	MF	0.293132	71
GO:0016670	oxidoreductase activity, acting on a sulfur group of dor	MF	0.299874	2
GO:0005871	kinesin complex	CC	0.304969	26
GO:0009308	amine metabolic process	BP	0.305223	22
GO:0032403	protein complex binding	MF	0.310715	34
GO:0016884	carbon-nitrogen ligase activity, with glutamine as amid	MF	0.348568	10
GO:0016984	ribulose-bisphosphate carboxylase activity	MF	0.349146	1
GO:0030001	metal ion transport	BP	0.383562	64
GO:0015630	microtubule cytoskeleton	CC	0.387279	42
GO:0006996	organelle organization	BP	0.39661	114
GO:0042254	ribosome biogenesis	BP	0.408366	32
GO:0003777	microtubule motor activity	MF	0.408366	26
GO:0007018	microtubule-based movement	BP	0.408366	26
GO:0005515	protein binding	MF	0.409794	1091
GO:0046394	carboxylic acid biosynthetic process	BP	0.424385	91
GO:0071944	cell periphery	CC	0.429207	50
GO:0050661	NADP binding	MF	0.434328	16
GO:0008168	methyltransferase activity	MF	0.445785	101
GO:0019752	carboxylic acid metabolic process	BP	0.469403	199
GO:0007017	microtubule-based process	BP	0.469818	42
GO:0006694	steroid biosynthetic process	BP	0.490951	6
GO:0004488	methylenetetrahydrofolate dehydrogenase (NADP+) ac	MF	0.539052	3
GO:0004075	biotin carboxylase activity	MF	0.560554	1
GO:0044723	single-organism carbohydrate metabolic process	BP	0.61251	94
GO:0046856	phosphatidylinositol dephosphorylation	BP	0.641803	5
GO:0007186	G-protein coupled receptor signaling pathway	BP	0.650777	10
GO:0046129	purine ribonucleoside biosynthetic process	BP	0.79429	24
GO:0045261	proton-transporting ATP synthase complex, catalytic co	CC	0.801076	3
GO:0003993	acid phosphatase activity	MF	0.814544	4
GO:0046486	glycerolipid metabolic process	BP	0.820036	28
GO:0042398	cellular modified amino acid biosynthetic process	BP	0.840114	12
GO:0045735	nutrient reservoir activity	MF	0.870613	25

GO:0016835	carbon-oxygen lyase activity	MF	0.904379	22
GO:0042545	cell wall modification	BP	0.908764	19
GO:0042578	phosphoric ester hydrolase activity	MF	0.931734	32
GO:0006575	cellular modified amino acid metabolic process	BP	0.948658	14

d7930.64,evm.model.scaffold8620.8,evm.model.scaffold906.31,evm.model.scaffold9237.30,evm.mod
odel.scaffold1433.65,evm.model.scaffold1518.88,evm.model.scaffold182551.112,evm.model.scaffold

l.scaffold2276.6,evm.model.scaffold2443.10,evm.model.scaffold2443.46,evm.model.scaffold2443.8,e
n.model.scaffold8877.7,evm.model.scaffold10356.33,evm.model.scaffold10356.34,evm.model.scaffol

el.scaffold9237.44,evm.model.scaffold931.49,evm.model.scaffold931.52,evm.model.scaffold155.15,e
d1914.28,evm.model.scaffold2059.39,evm.model.scaffold2227.88,evm.model.scaffold320308.25,evm

evm.model.scaffold3046.17,evm.model.scaffold5018.73,evm.model.scaffold5091.35,evm.model.scaff
ld3459.49,evm.model.scaffold4037.14,evm.model.scaffold569.195,evm.model.scaffold571.21,evm.m

evm.model.scaffold1754.50,evm.model.scaffold1754.53,evm.model.scaffold17865.5,evm.model.scaff
.model.scaffold16207.14,evm.model.scaffold2132.43,evm.model.scaffold3953.14,evm.model.scaffol

old5091.9,evm.model.scaffold6279.3,evm.model.scaffold6952.7,evm.model.scaffold7033.47,evm.mc
odel.scaffold66.37,evm.model.scaffold670.36,evm.model.scaffold14521.7,evm.model.scaffold218.25

old1927.28,evm.model.scaffold3273.2,evm.model.scaffold393.12,evm.model.scaffold691.4,evm.moc
d4900.13,evm.model.scaffold5406.1,evm.model.scaffold550.36,evm.model.scaffold1000.23,evm.mo

odel.scaffold769.21,evm.model.scaffold8403.16,evm.model.scaffold8757.27,evm.model.scaffold1163.
,evm.model.scaffold4074.21,evm.model.scaffold8387.2,evm.model.scaffold874.16,evm.model.scaffo

del.scaffold1305.6,evm.model.scaffold1328.25,evm.model.scaffold139890.1,evm.model.scaffold1521[illegible]
del.scaffold1433.27.1,evm.model.scaffold1620.22,evm.model.scaffold320340.10,evm.model.scaffold[illegible]

.5,evm.model.scaffold12234.50,evm.model.scaffold16207.13,evm.model.scaffold3953.11,evm.model
ld14377.3,evm.model.scaffold155.18,evm.model.scaffold1927.26,evm.model.scaffold1985.83,evm.m

9.74,evm.model.scaffold18843.1,evm.model.scaffold1960.1,evm.model.scaffold2032.164,evm.model
4924.14,evm.model.scaffold775.17,evm.model.scaffold1433.32.1,evm.model.scaffold27907.1,evm.mo

.scaffold4281.6,evm.model.scaffold4466.39,evm.model.scaffold4476.75,evm.model.scaffold4870.9,ev
odel.scaffold6038.16,evm.model.scaffold2223.83,evm.model.scaffold1080.22,evm.model.scaffold12

.scaffold2491.60,evm.model.scaffold3010.71,evm.model.scaffold4540.25,evm.model.scaffold4782.32
odel.scaffold3053.19,evm.model.scaffold3448.35,evm.model.scaffold439.54,evm.model.scaffold439.5

vm.model.scaffold52.73,evm.model.scaffold5650.32,evm.model.scaffold7223.3,evm.model.scaffold9
290.3,evm.model.scaffold1755.131,evm.model.scaffold353.103,evm.model.scaffold5793.117,evm.mc

2,evm.model.scaffold5719.10,evm.model.scaffold5804.1,evm.model.scaffold5932.3,evm.model.scaffo
58,evm.model.scaffold5739.2.1,evm.model.scaffold811.70,evm.model.scaffold1339.2,evm.model.sca

43.110,evm.model.scaffold970.69,evm.model.scaffold1279.4.1,evm.model.scaffold1651.20,evm.mod
odel.scaffold7930.8,evm.model.scaffold1813.61,evm.model.scaffold1935.1,evm.model.scaffold32034

ɔld799.38,evm.model.scaffold811.103,evm.model.scaffold811.112,evm.model.scaffold8156.11,evm.m
ffold1716.2,evm.model.scaffold3517.49,evm.model.scaffold3532.20,evm.model.scaffold6997.25,evm

el.scaffold1651.21,evm.model.scaffold3601.101,evm.model.scaffold362.102,evm.model.scaffold372.
1.51.1,evm.model.scaffold4723.2,evm.model.scaffold5553.3.1,evm.model.scaffold1651.52,evm.mode

nodel.scaffold5547.14,evm.model.scaffold10327.28,evm.model.scaffold10526.10,evm.model.scaffold
.model.scaffold1038.67,evm.model.scaffold1718.27,evm.model.scaffold1996.19,evm.model.scaffold

148,evm.model.scaffold4952.21,evm.model.scaffold634.35,evm.model.scaffold647.17,evm.model.sc
el.scaffold2757.42,evm.model.scaffold3406.28,evm.model.scaffold3406.40,evm.model.scaffold3531
