# Supplementary material for: Genome and transcriptome of Papaver somniferum Chinese landrace CHM indicates that massive genome expansion contributes to high benzylisoquinoline alkaloid biosynthesis
Source: Hortic Res. 2021 Jan 1;8:5. doi: 10.1038/s41438-020-00435-5 (PMC7775465; doi:10.1038/s41438-020-00435-5)
Supplement: Supplementary file 41 — Table S19 [file 41438_2020_435_MOESM41_ESM.pdf]

Table S20. 4dTv of genes from Pso and Mco as to their Aco orthologs

	4dtv	
	**Pso**	**Mco**
**Mean**	0.464	0.364
**Stdev**	0.0474	0.0364
