# Supplementary material for: Genome and transcriptome of Papaver somniferum Chinese landrace CHM indicates that massive genome expansion contributes to high benzylisoquinoline alkaloid biosynthesis
Source: Hortic Res. 2021 Jan 1;8:5. doi: 10.1038/s41438-020-00435-5 (PMC7775465; doi:10.1038/s41438-020-00435-5)
Supplement: Supplementary file 45 — Table S23 [file 41438_2020_435_MOESM45_ESM.pdf]

Table S23. Summary of the paralogous gene pairs detected in PSO, MCO, ACO, and NUL

Core pathway: L-Tyrosine → (S)-Reticuline	PSO	MCO	ACO	NUL
TYDC	13	14	3	7
TyrAT	3	4	3	3
NCS	2	10	10	4
6OMT	1	1	1	4
CNMT	2	6	4	1
NMCH	3	1	3	1
4OMT	8	1	1	0
Morphine,Codeine and Thebaine biosynthesis pasthway	PSO	MCO	ACO	NUL
STORR	1	0	0	0
SalSyn/CYP719B1	2	0	0	0
SalR	2	3	3	4
SalAT	1	0	0	0
T6ODM	2	4	10	10
COR	2	2	5	5
CODM	2	3	5	3
Papaverine biosynthesis pasthway	PSO	MCO	ACO	NUL
N7OMT	3	0	0	0
DBOX	2	32	6	8
Noscapine biosynthesis pasthway	PSO	MCO	ACO	NUL
BBE	3	2	4	0
SOMT1	1	1	1	1
CanSyn/CYP719A21	1	1	7	0
TNMT	3	2	4	0
CYP82Y1	1	0	0	0
CYP82X2	1	0	0	0
AT1	1	0	0	0
CYP82X1	1	0	0	0
SOMT2	1	2	0	0
CXE1	1	5	0	0
NOS	1	3	3	0
Sanguinarine biosynthesis pasthway	PSO	MCO	ACO	NUL
CFS	3	1	0	0
SPS	2	1	0	0
MSH	2	1	1	1
P6H	2	6	0	1

*Yellow marked genes mean they are efficacy for more than one pathway.
