# Supplementary material for: Genome and transcriptome of Papaver somniferum Chinese landrace CHM indicates that massive genome expansion contributes to high benzylisoquinoline alkaloid biosynthesis
Source: Hortic Res. 2021 Jan 1;8:5. doi: 10.1038/s41438-020-00435-5 (PMC7775465; doi:10.1038/s41438-020-00435-5)
Supplement: Supplementary file 46 — Table S24 [file 41438_2020_435_MOESM46_ESM.pdf]

Table S24. Summary of identified SNPs compared to HN1

	**Number of SNPs**
**Total**	12,563,129
**Homozygous**	9,213,562
**Heterozygous**	3,349,567
**Intergenic**	10,964,629
**Intronic**	641,193
**CDS**	309,236
**Synonymous**	133,154
**Nonsynonymous**	170,136
**Splicing**	1,567
**Stop gain/loss**	4,686
