# Supplementary material for: Genome and transcriptome of Papaver somniferum Chinese landrace CHM indicates that massive genome expansion contributes to high benzylisoquinoline alkaloid biosynthesis
Source: Hortic Res. 2021 Jan 1;8:5. doi: 10.1038/s41438-020-00435-5 (PMC7775465; doi:10.1038/s41438-020-00435-5)
Supplement: Supplementary file 47 — Table S25 [file 41438_2020_435_MOESM47_ESM.pdf]

Table S25. Summary of identified Indels compared to HN1

	Number of Indels
**Total**	1,612,314
**Intergenic**	1,313,139
**Intronic**	123,435
**CDS**	19,158
**Frameshift**	11,470
**Splicing**	391
**Stop gain/loss**	860
