# Supplementary material for: Genome and transcriptome of Papaver somniferum Chinese landrace CHM indicates that massive genome expansion contributes to high benzylisoquinoline alkaloid biosynthesis
Source: Hortic Res. 2021 Jan 1;8:5. doi: 10.1038/s41438-020-00435-5 (PMC7775465; doi:10.1038/s41438-020-00435-5)
Supplement: Supplementary file 50 — Table S28 [file 41438_2020_435_MOESM50_ESM.pdf]

Table S28. SVs between CHM and NH1 in BIA gene cluster.

Chr	Start	end	detail	Type	Annotation
**chr11**	127950154	127950215	INV:a 62bp inversion	intergenic	PS1126450.1,PS1126460.1
**chr11**	127950252	127950585	INV:a 334bp inversion	intergenic	PS1126450.1,PS1126460.1
**chr11**	127996218	127996284	INV:a 67bp inversion	intergenic	PS1126480.1,PS1126490.1
**chr11**	128003157	128004464	INV:a 1308bp inversion	exonic	PS1126490.1
**chr11**	128004895	128005105	INV:a 211bp inversion	upstream	PS1126490.1
**chr11**	128019730	128020533	INV:a 804bp inversion	intergenic	PS1126490.1,PS1126500.1
**chr11**	128022134	128022255	INV:a 122bp inversion	intergenic	PS1126490.1,PS1126500.1
**chr11**	128022257	128025234	INV:a 2978bp inversion	intergenic	PS1126490.1,PS1126500.1
**chr11**	128022796	128022855	DEL:a 60bp deletion	intergenic	PS1126490.1,PS1126500.1
**chr11**	128028420	128028507	INV:a 88bp inversion	intergenic	PS1126490.1,PS1126500.1
**chr11**	128028509	128028906	INV:a 398bp inversion	intergenic	PS1126490.1,PS1126500.1
**chr11**	128038916	128039177	INV:a 262bp inversion	intergenic	PS1126490.1,PS1126500.1
**chr11**	128040219	128040599	INV:a 381bp inversion	intergenic	PS1126490.1,PS1126500.1
**chr11**	128065321	128065918	INV:a 598bp inversion	intergenic	PS1126490.1,PS1126500.1
**chr11**	128067666	128067801	INV:a 136bp inversion	downstream	PS1126500.1
**chr11**	128142046	128142282	INV:a 237bp inversion	intergenic	PS1126550.1,PS1126555.1
**chr11**	127950154	127950215	INV:a 62bp inversion	intergenic	PS1126450.1,PS1126460.1
