# Supplementary material for: Genome and transcriptome of Papaver somniferum Chinese landrace CHM indicates that massive genome expansion contributes to high benzylisoquinoline alkaloid biosynthesis
Source: Hortic Res. 2021 Jan 1;8:5. doi: 10.1038/s41438-020-00435-5 (PMC7775465; doi:10.1038/s41438-020-00435-5)
Supplement: Supplementary file 51 — Table S29 [file 41438_2020_435_MOESM51_ESM.pdf]

Table S29. SNPs between CHM and HN1 in BIA gene cluster.

Chr	position	HN1	CHM	details	type
**chr11**	127811830	C	T	PS1126400.1:c.C347T:p.T116I,	nonsynonymous
**chr11**	127859689	A	T	PS1126430.1:c.A303T:p.R101S,	nonsynonymous
**chr11**	128003164	G	A	PS1126490.1:c.C1035T:p.F345F,	synonymous
**chr11**	128003170	G	A	PS1126490.1:c.C1029T:p.S343S,	synonymous
**chr11**	128003192	A	T	PS1126490.1:c.T1007A:p.L336X,	stopgain
**chr11**	128003209	C	T	PS1126490.1:c.G990A:p.K330K,	synonymous
**chr11**	128003217	C	G	PS1126490.1:c.G982C:p.G328R,	nonsynonymous
**chr11**	128003225	T	C	PS1126490.1:c.A974G:p.K325R,	nonsynonymous
**chr11**	128003234	A	T	PS1126490.1:c.T965A:p.F322Y,	nonsynonymous
**chr11**	128003245	C	G	PS1126490.1:c.G954C:p.A318A,	synonymous
**chr11**	128003263	C	T	PS1126490.1:c.G936A:p.P312P,	synonymous
**chr11**	128003269	A	G	PS1126490.1:c.T930C:p.L310L,	synonymous
**chr11**	128003272	A	G	PS1126490.1:c.T927C:p.V309V,	synonymous
**chr11**	128003275	G	T	PS1126490.1:c.C924A:p.S308S,	synonymous
**chr11**	128003311	C	T	PS1126490.1:c.G888A:p.L296L,	synonymous
**chr11**	128003388	T	C	PS1126490.1:c.A811G:p.S271G,	nonsynonymous
**chr11**	128003410	T	C	PS1126490.1:c.A789G:p.A263A,	synonymous
**chr11**	128003571	C	T	PS1126490.1:c.G750A:p.M250I,	nonsynonymous
**chr11**	128003583	G	A	PS1126490.1:c.C738T:p.N246N,	synonymous
**chr11**	128003586	G	C	PS1126490.1:c.C735G:p.L245L,	synonymous
**chr11**	128003588	G	A	PS1126490.1:c.C733T:p.L245F,	nonsynonymous
**chr11**	128003589	G	A	PS1126490.1:c.C732T:p.C244C,	synonymous
**chr11**	128003618	G	A	PS1126490.1:c.C703T:p.Q235X,	stopgain
**chr11**	128003636	G	A	PS1126490.1:c.C685T:p.P229S,	nonsynonymous
**chr11**	128003637	A	G	PS1126490.1:c.T684C:p.Y228Y,	synonymous
**chr11**	128003702	G	T	PS1126490.1:c.C619A:p.L207I,	nonsynonymous
**chr11**	128003736	T	C	PS1126490.1:c.A585G:p.L195L,	synonymous
**chr11**	128003761	A	G	PS1126490.1:c.T560C:p.I187T,	nonsynonymous
**chr11**	128003779	G	C	PS1126490.1:c.C542G:p.T181S,	nonsynonymous
**chr11**	128003788	C	T	PS1126490.1:c.G533A:p.G178D,	nonsynonymous
**chr11**	128003798	T	C	PS1126490.1:c.A523G:p.I175V,	nonsynonymous
**chr11**	128003808	T	C	PS1126490.1:c.A513G:p.T171T,	synonymous
**chr11**	128003882	T	C	PS1126490.1:c.A439G:p.T147A,	nonsynonymous
**chr11**	128003914	G	C	PS1126490.1:c.C407G:p.S136W,	nonsynonymous
**chr11**	128003943	C	T	PS1126490.1:c.G378A:p.L126L,	synonymous
**chr11**	128003954	T	C	PS1126490.1:c.A367G:p.M123V,	nonsynonymous
**chr11**	128004012	T	C	PS1126490.1:c.A309G:p.E103E,	synonymous
**chr11**	128004054	C	T	PS1126490.1:c.G267A:p.L89L,	synonymous
**chr11**	128004091	A	G	PS1126490.1:c.T230C:p.I77T,	nonsynonymous
**chr11**	128004117	C	T	PS1126490.1:c.G204A:p.L68L,	synonymous
**chr11**	128004127	C	A	PS1126490.1:c.G194T:p.S65I,	nonsynonymous
**chr11**	128004253	T	C	PS1126490.1:c.A68G:p.K23R,	nonsynonymous
**chr11**	128004265	G	T	PS1126490.1:c.C56A:p.T19N,	nonsynonymous
**chr11**	128004279	A	G	PS1126490.1:c.T42C:p.N14N,	synonymous
**chr11**	128128183	C	G	PS1126550.1:c.C378G:p.F126L,	nonsynonymous
**chr11**	128128472	G	A	PS1126550.1:c.G667A:p.V223I,	nonsynonymous
**chr11**	128247503	A	G	PS1126570.1:c.T82C:p.L28L,	synonymous
**chr11**	128331654	T	C	PS1126620.1:c.T837C:p.Y279Y,	synonymous
**chr11**	128331663	A	C	PS1126620.1:c.A846C:p.E282D,	nonsynonymous
**chr11**	128331673	G	A	PS1126620.1:c.G856A:p.E286K,	nonsynonymous
**chr11**	128341053	C	T	PS1126625.1:c.C12T:p.L4L,	synonymous
**chr11**	128341577	A	G	PS1126625.1:c.A381G:p.E127E,	synonymous
